# Supplementary material for: A CSF-1R-blocking antibody/IL-10 fusion protein increases anti-tumor immunity by effectuating tumor-resident CD8+ T cells
Source: Cell Rep Med. 2023 Aug 15;4(8):101154. doi: 10.1016/j.xcrm.2023.101154 (PMC10439276; doi:10.1016/j.xcrm.2023.101154)
Supplement: Document S1. Figures S1–S11 [file mmc1.pdf]

Cell Reports Medicine, Volume *4*

Supplemental information

A CSF-1R-blocking antibody/IL-10 fusion protein increases anti-tumor immunity by effectuating tumor-resident CD8$^+$ T cells

Yao-Wen Chang, Huey-Wen Hsiao, Ju-Pei Chen, Sheue-Fen Tzeng, Chin-Hsien Tsai, Chun-Yi Wu, Hsin-Hua Hsieh, Santiago J. Carmona, Massimo Andreatta, Giusy Di Conza, Mei-Tzu Su, Pandelakis A. Koni, Ping-Chih Ho, Hung-Kai Chen, and Muh-Hwa Yang

Supplemental information

The file includes:

-Supplementary Figure S1 to S11 with legends

A CSF-1R-blocking antibody/IL-10 fusion protein increases anti-tumor immunity by effectuating tumor-resident CD8$^+$ T cells

Yao-Wen Chang, Huey-Wen Hsiao, Ju-Pei Chen, Sheue-Fen Tzeng, Chin-Hsien Tsai, Chun-Yi Wu, Hsin-Hua Hsieh, Santiago J. Carmona, Massimo Andreatta, Giusy Di Conza, Mei-Tzu Su, Pandelakis A. Koni, Ping-Chih Ho, Hung-Kai Chen, and Muh-Hwa Yang

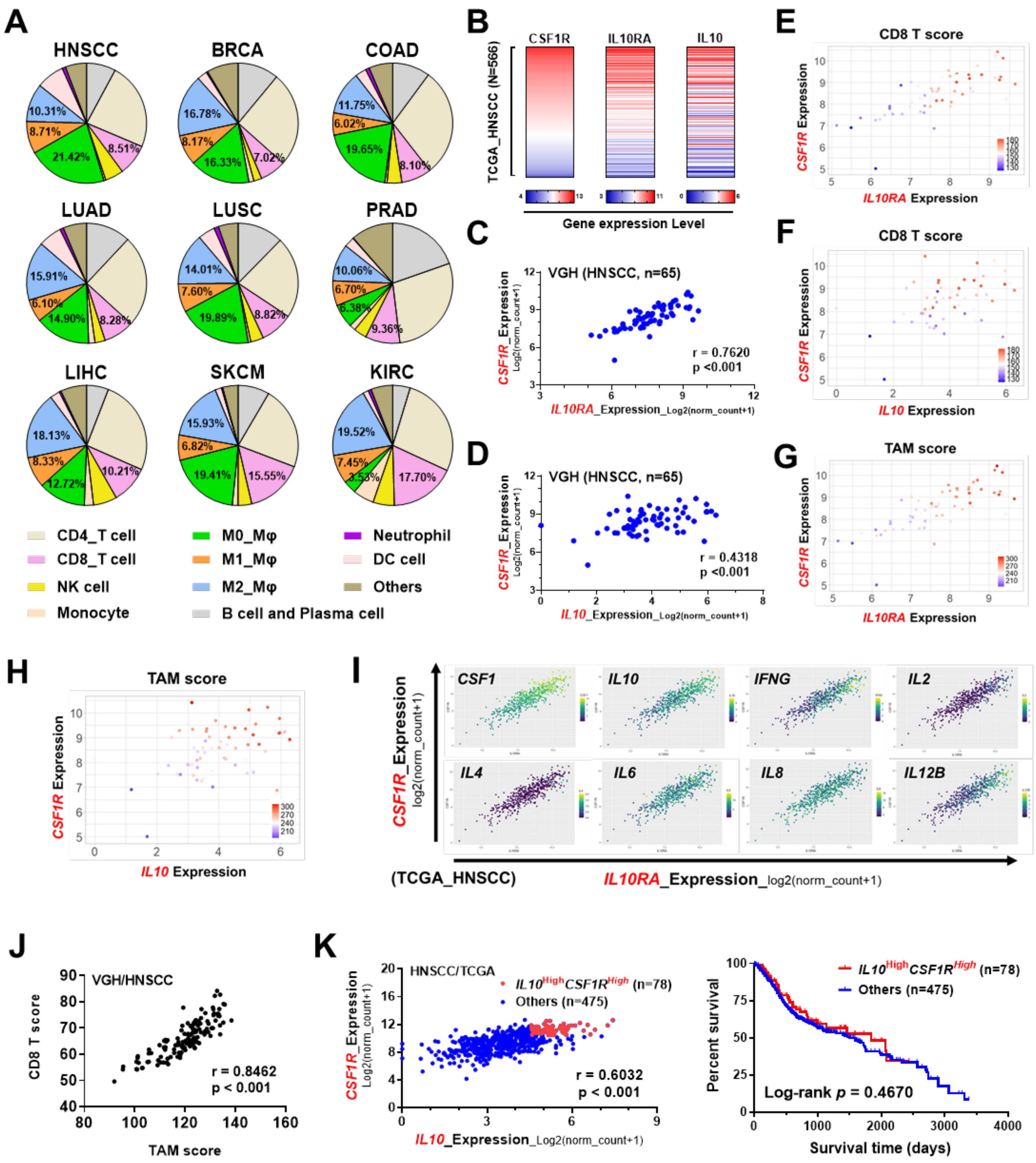

Figure S1. The immune cell composition in different cancers and the relationship between CSF-1R and IL10/IL10-RA axis and immune signature in HNSCC, related to Figure 1. (A) The proportion of infiltrated immune cells within tumor microenvironment. Bulk RNA-sequencing analysis from 9 cancer types of TCGA database. Relative abundance of immune cell types among different cancer types was analyzed by CIBERSORTx with LM22 signature. head and neck squamous cell carcinoma (HNSCC); breast cancer (BRCA); colon adenocarcinoma (COAD); lung adenocarcinoma (LUAD); lung squamous cell carcinoma (LUSC); prostate adenocarcinoma (PRAD); liver cancer (LIHC); skin cutaneous melanoma (SKCM); kidney renal clear cell carcinoma (KIRC). **(B)** The gene expression panels of CSF1R, IL-10RA and IL-10 of the individuals of TCGA HNSCC patients (n= 566). **(C),(D)** The correlation analysis of RNA expression level between IL-10/IL-10-RA axis and CSF-1R of head and neck squamous carcinoma (HNSCC) in Taipei Veterans General Hospital (VGH) database (n=65). The correlation was determined by

Pearson correlation (r). **(E)-(H)** The 3D scatter plots for analyzing the expression of CD8 T score and tumor -associated macrophages (TAM) score related to IL10/IL10RA axis and CSF1R in VGH-HNSC. X-axis represents IL10 or IL10-RA expression. Y-axis means CSF1R expression. CD8 T or TAM scores were expressed from blue to red gradient color dots (low to high). The value expressed by RNA level (log2(norm_count+1)). **(I)** The 3D scatter plots for analyzing the expression of different cytokine genes related to IL10-RA and CSF1R in TCGA-HNSC. **(J)** The correlation analysis of the gene signature scores between CD8 T and TAM of VGH/HNSCC DSP dataset. RNA expression level from 134 ROIs of 6 patients were assessed and Region of interests (ROIs) were categorized as peri-normal tissue (Normal), primary tumor (inner tumor and outer tumor) and metastatic lymph node (LN). The correlation was determined by Pearson correlation (r). **(K)** Left panel: a scatter plot of IL10 and CSF1R gene expression level in TCGA HNSCC patients. Red dots indicate the patients with the top 50% level of IL10 (IL-10^{High}) and top 50% of CSF1R (CSF1R^{High}) gene expression level. Right panel: survival analysis between groups of IL10^{High}CSF1R^{High} (red line) and others (blue line). P value was estimated by log-rank test.

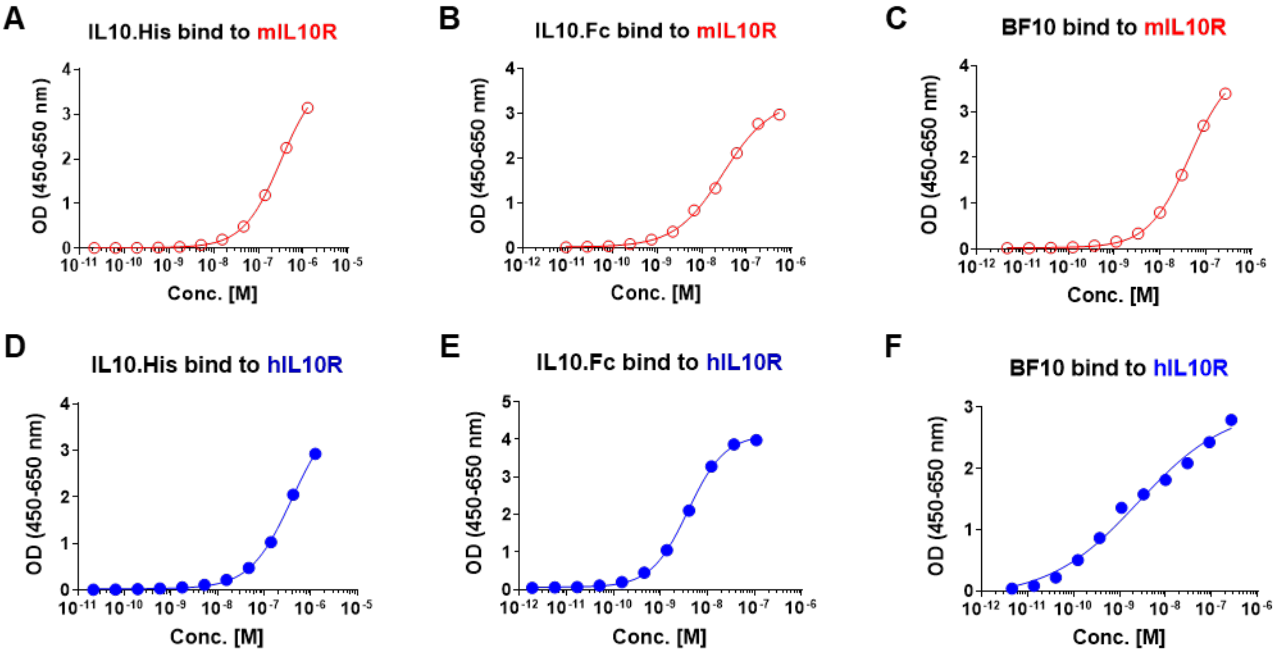

G

ELISA Bnding Assay (EC50 = M)			
Type	IL10	IL10.Fc	BF10
Bind to human IL-10RA	$3.95x10^{-7}$	$3.84x10^{-9}$	$2.55x10^{-9}$
Bind to mouse IL-10RA	$3.16x10^{-7}$	$3.00x10^{-8}$	$4.47x10^{-8}$

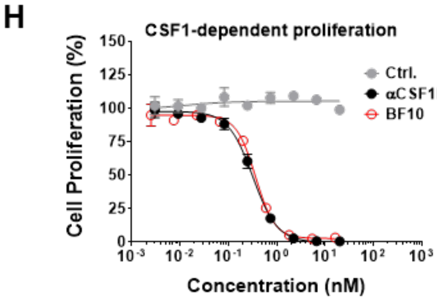

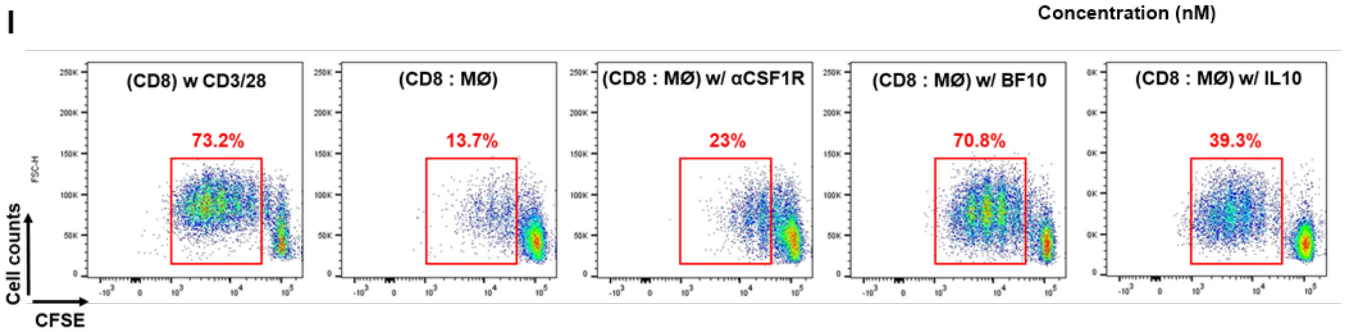

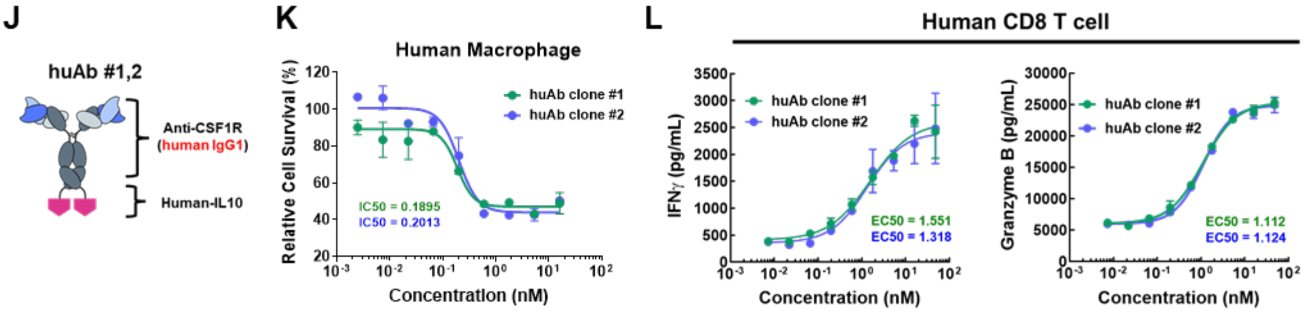

Figure S2. In vitro characterization of the *α*-CSF1R-IL10 fusion proteins, related to Figure 2. (A)-(F) Dose-response binding curve of IL-10-Histag, IL10-Fc and BF10 to recombinant mouse or human IL-10 receptor (a-c for mouse and d-f for human, respectively). The binding assay were performed by ELISA and data are calculated by OD values. **(G)** A table for summarizing the calculated EC50 values of BF10 compared to IL10-histag and IL10-Fc. EC50 values were calculated from three independent experiments. **(H)** Proliferation assay of M-NFS-60 murine myelogenous leukemia cells incubated with CSF-1 together with anti-mCSF-1R or BF10 or control for 3 days. Cell proliferation was measured using CellTiter-Glo assay (Promega). **(I)** Effects of

BF10 on T cell proliferation. CFSE-labeled $CD8^+$ T cells were activated with anti-CD3 and anti-CD28 in absence or presence of IL4-primed BMDMs. The activated cells were treated with 50 ng/ml of anti-CSF-1R, IL-10 or BF10 for 72 hours, and collected for flow cytometry analysis. The number indicates proliferating CD8 T cells percent in the red bracket. **(J)** Schematic presentation of the anti-human CSF-1R-IL10 fusion proteins (named as huAb # clone 1 and 2). **(K)** Cell viability assay**.** Human macrophages were derived from $CD14^+$ monocytes and then incubated with CSF-1 with different concentrations of anti-hCSF-1R-IL-10 fusion proteins for 3 days. Cell survival was measured using CellTiter-Glo assay (Promega). **(L)** Assay for the production of IFN-γ and granzyme B from human activated $CD8^+$ T cells. CD 8^+ T cells were isolated from PBMC and activated with anti-CD3 plus anti-CD 28 (T Cell TransAct Miltenyi Biotec) for 3 days and then treated for 3 days with anti CSF-1R/IL-10 fusion proteins and triggered with anti-CD3 (Biolegend) for 4 hours. The level of IFN-γ and granzyme B were measured by ELISA (Biolegend). The calculated curve of one representative data of three independent experiments was presented as mean ± S.D. and was shown in indicated assays in j and k, respectively.

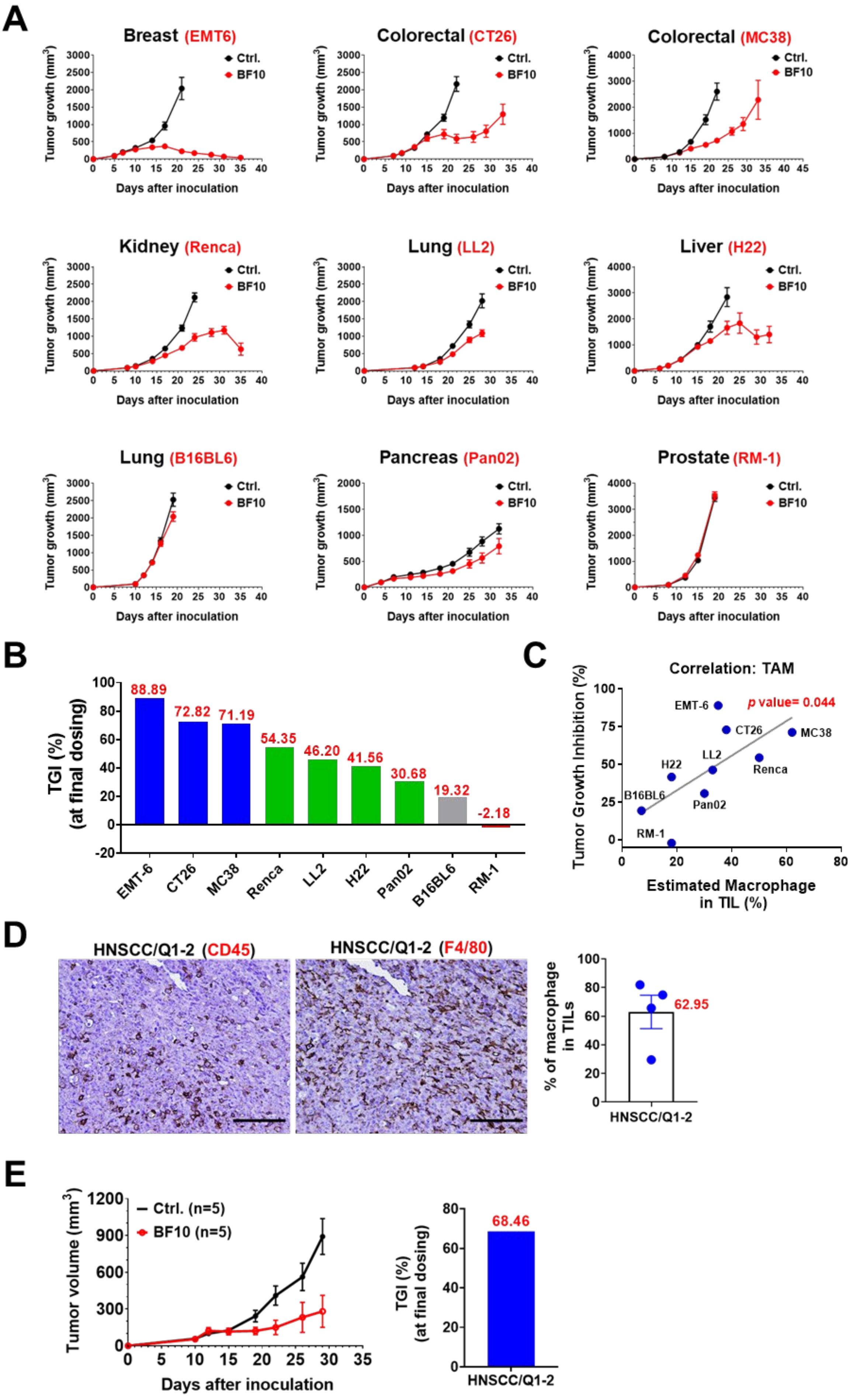
A
Breast (EMT6)
Colorectal (CT26)
Colorectal (MC38)
Kidney (Renca)
Lung (LL2)
Liver (H22)
Lung (B16BL6)
Pancreas (Pan02)
Prostate (RM-1)
Ctrl.
BF10
Tumor growth (mm³)
Days after inoculation
B
TGI (%) (at final dosing)
88.89
72.82
71.19
54.35
46.20
41.56
30.68
19.32
-2.18
EMT-6
CT26
MC38
Renca
LL2
H22
Pan02
B16BL6
RM-1
C
Correlation: TAM
Tumor Growth Inhibition (%)
p value= 0.044
Estimated Macrophage in TIL (%)
D
HNSCC/Q1-2 (CD45)
HNSCC/Q1-2 (F4/80)
% of macrophage in TILs
62.95
HNSCC/Q1-2
E
Ctrl. (n=5)
BF10 (n=5)
Tumor volume (mm³)
Days after inoculation
TGI (%) (at final dosing)
68.46
HNSCC/Q1-2

Figure S3. The antitumor activities of BF10 in mouse syngeneic tumor models, related to Figure 3. (A) The anti-tumor activity of BF10 was further assessed among night cancer cell lines for seven cancer types. The syngeneic mouse cancer platform (MuScreen^{TM}, CrownBio Inc.) was applied and analyzed with their responding macrophage proportion in the same model (CrownBio Inc.). Tumor-bearing mice were received the treatment with either isotype control or BF10 (36 mg/kg) for a total of six doses. All mice received the equivalent dose treatment as described in methods. The tumor growth was measured twice per week until end of the study. **(B)** The tumor growth inhibition (TGI) values (presented in %) among different cancer types. TGI was calculated from the ratio of volume change in treatment and control between Day 0 and the end of the experiment (Day 33). **(C)** The correlation analysis of TGI versus estimated macrophages numbers in tumor-infiltrated lymphocytes (TILs). The relationship was evaluated by linear regression model and shown as p-value. **(D)-(E)** The anti-tumor activity of BF10 for mouse HNSCC syngeneic model. HNSCC/Q1-2 tumor-bearing mice were received treatments of Ctrl-IgG or BF10 for six doses, and then tumor samples were harvested and subjected to determine the infiltration of $CD45^+$ and $F4/80^+$ cells by immunohistochemical staining. Representative images of the $CD45^+$ cells or $F4/80^+$ cells from the control group and BF10 group were calculated for macrophage proportion in TIL. The TGI value was calculated from the ratio of volume change in treatment and control between Day 0 and the end of the experiment (Day 29).

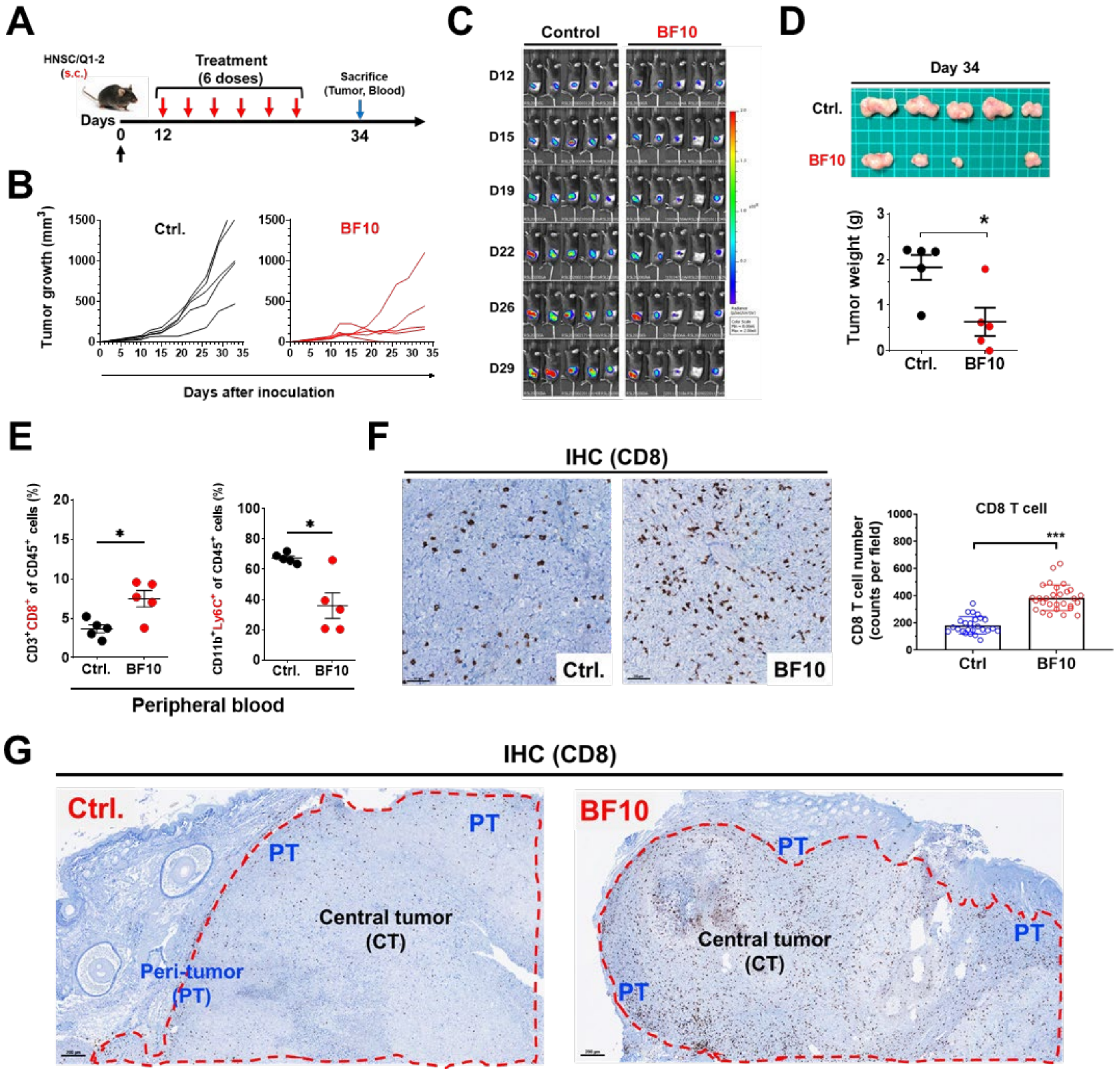

Figure S4. The antitumor activities of BF10 in mouse syngeneic tumor models, related to Figure 3. (A)-(F) Tumor-bearing mice (HNSCC/Q-2^{lucferiease}, subcutaneous) received control IgG (30 mg/kg) or BF10 (an equivalent dose of 30 mg/kg IgG) twice per week for a total of six doses when tumor size reach to 100 mm^3 (Day 12). At endpoint of treatment (Day 34), tumor tissues and serum samples were collected for analysis. **(A)** Experimental scheme and treatment protocol of mouse model of HNSCC cancer cells. **(B)** Tumor volume curves of individual mice. **(C)** Representative bioluminescence images for tumor growth. The bioluminescence intensity of tumor growth (Day 12, 15, 19, 22 26 and Day 29) was measured using Xenogen IVIS 100 imaging system. n=5 for each group. **(D)** Upper: representative photos of the collected tumors. Lower: quantification of tumor weights after treatment of BF10 at Day 34. *P < 0.05. **(E)** Flow cytometric analysis of the immune cells in peripheral blood of the tumor-bearing mice after last treatment (n = 5 per group). **(F)** The representative image of the tumor-infiltrated CD8$^+$ T cells with immunohistochemical (IHC) examination (Upper). Images were captured by a microscope system (Olympus BX51; Olympus Corp.). Scale bar= 50 μm. (Lower) The quantification of the CD8$^+$ T cells per HPF (20X magnification). Images from indicated groups (n= 3 to 4 mice, total images > 25 HPF) were quantified. Data were presented as mean ± S.D. **(G)** Representative images

of the distribution of tumor-infiltrated $CD8^+$ T cells. Tumor tissues (orthotopic murine HNSCC model) from the indicated treatments were examined by using IHC staining. Tumor tissue was separated into outer and inner regions which define as peri-tumor (PT, blue color) and central-tumor (CT, black color). Scale bar, 200 μm.

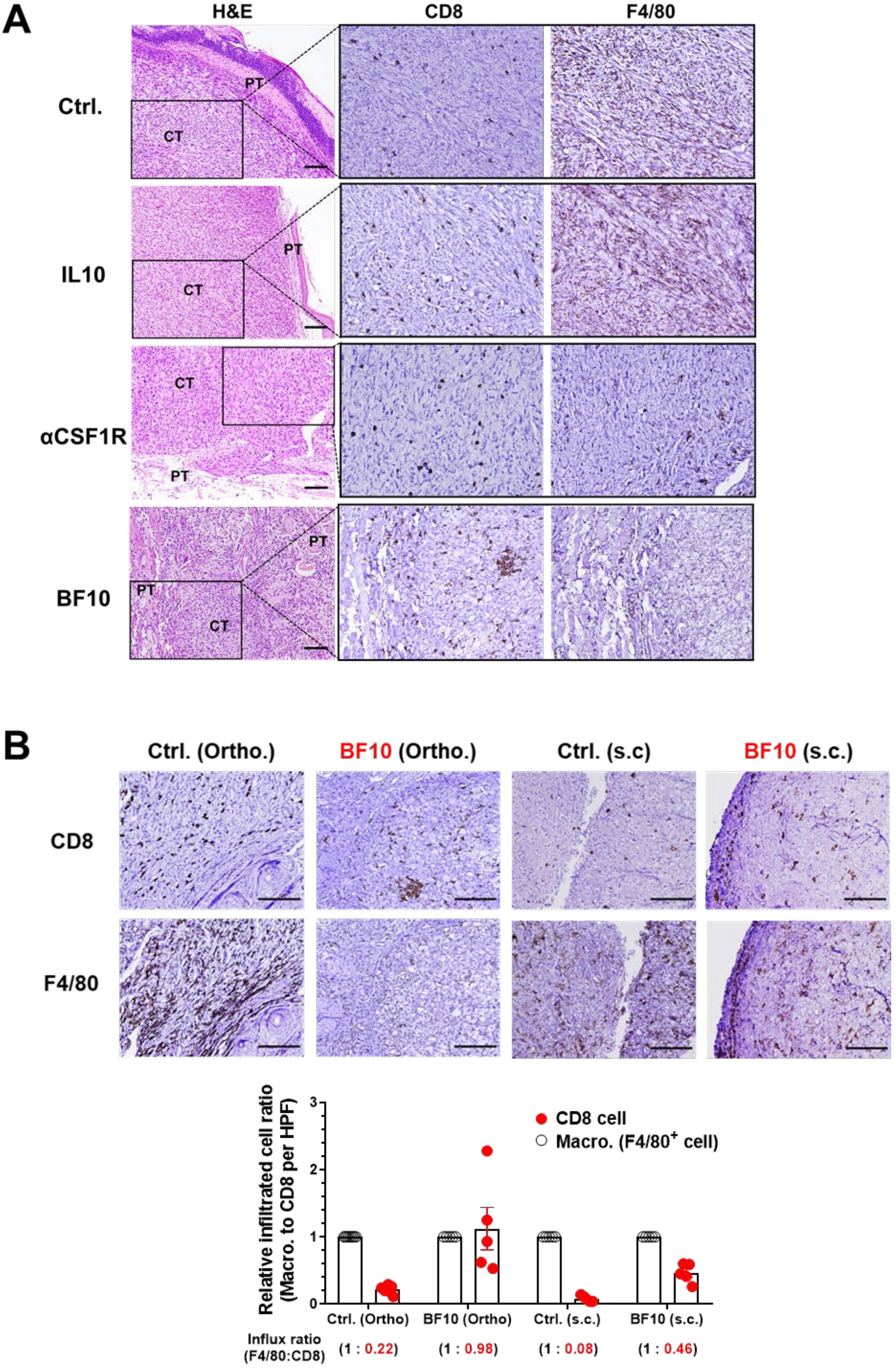

Figure S5. The antitumor activities of BF10 in mouse syngeneic tumor models, related to Figure 3. (A) Tumor-bearing mice (HNSCC/Q-2^{lucferiease}) received treatments of Ctrl-IgG (30 mg/kg), IL10-Fc (20 mg/kg), αCSF1R (30 mg/kg) or BF10 (36 mg/kg)

for six doses. Collected samples were assayed using immunohistochemical staining for CD8 T cells and tumor-resident macrophages. Representative images of H&E and IHC staining of $CD8^{+}$ T cells or $F4/80^{+}$ cells in tumor tissues from the indicated groups. n= 3 per group. Scale bar= 100 μm. **(B)** The ratio comparison of $CD8^{+}$: $F4/80^{+}$ cells between orthotopic and subcutaneous models of HNSCC/Q1-2 tumor cells. Representative image is one of at least two independent experiments. The quantification of the CD8+ T cells or macropahges per HPF (40X magnification). Images from indicated groups (n= 3 to 4 mice) were quantified. Data were presented as mean ± S.D. Scale bar = 100 μm

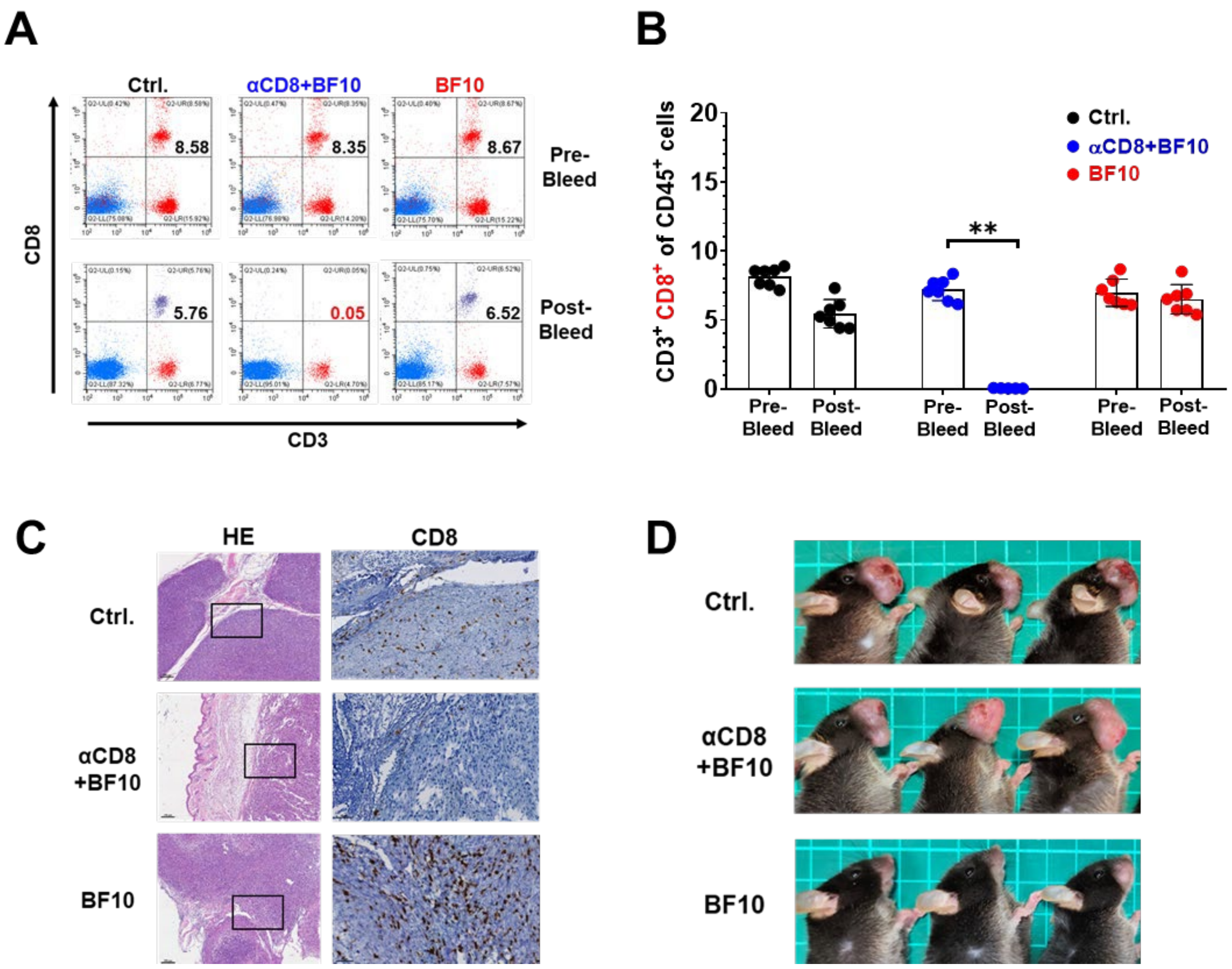

Figure S6. The antitumor activities of BF10 in mouse syngeneic tumor models, related to Figure 3. (A)-(B) $CD8^+$ T cells of peripheral blood of pre-bleed and post-bleed were assessed by flow cytometric analysis. The $CD8^+$ T cell depletion was carried out using 200 µg anti-CD8 antibody (i.p. every 3 days) simultaneously with or without BF10 treatment in tumor-bearing mice and protocols were described in Fig. 3I. **(C)** Representative images of H&E and IHC staining of $CD8^+$ T cells in tumor tissues from the indicated groups. n= 6-7 per group, **(D)** Representative photos of the orthotopic tumor from the indicated groups.

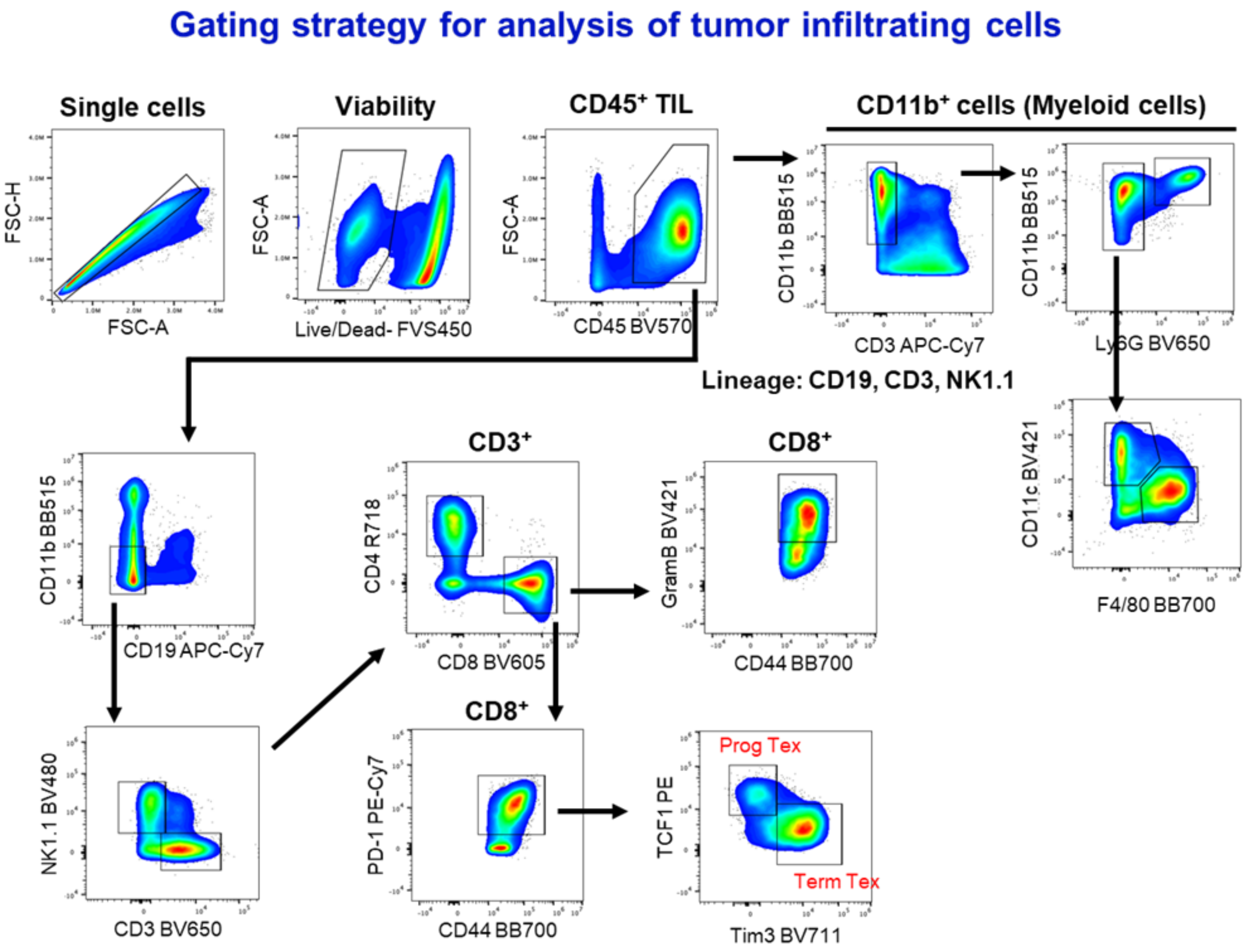

Figure S7. BF10 modulates immune cells in tumor and lymphatic organs, related to Figure 4. Gating strategies to assess immune compartmentalization of T cell subsets within tumor microenvironment**.** FACS gating strategy for immune cell profiling among tumors. The population and activity of infiltrated immune cells in the tumor microenvironment were determined according to the gating strategy.

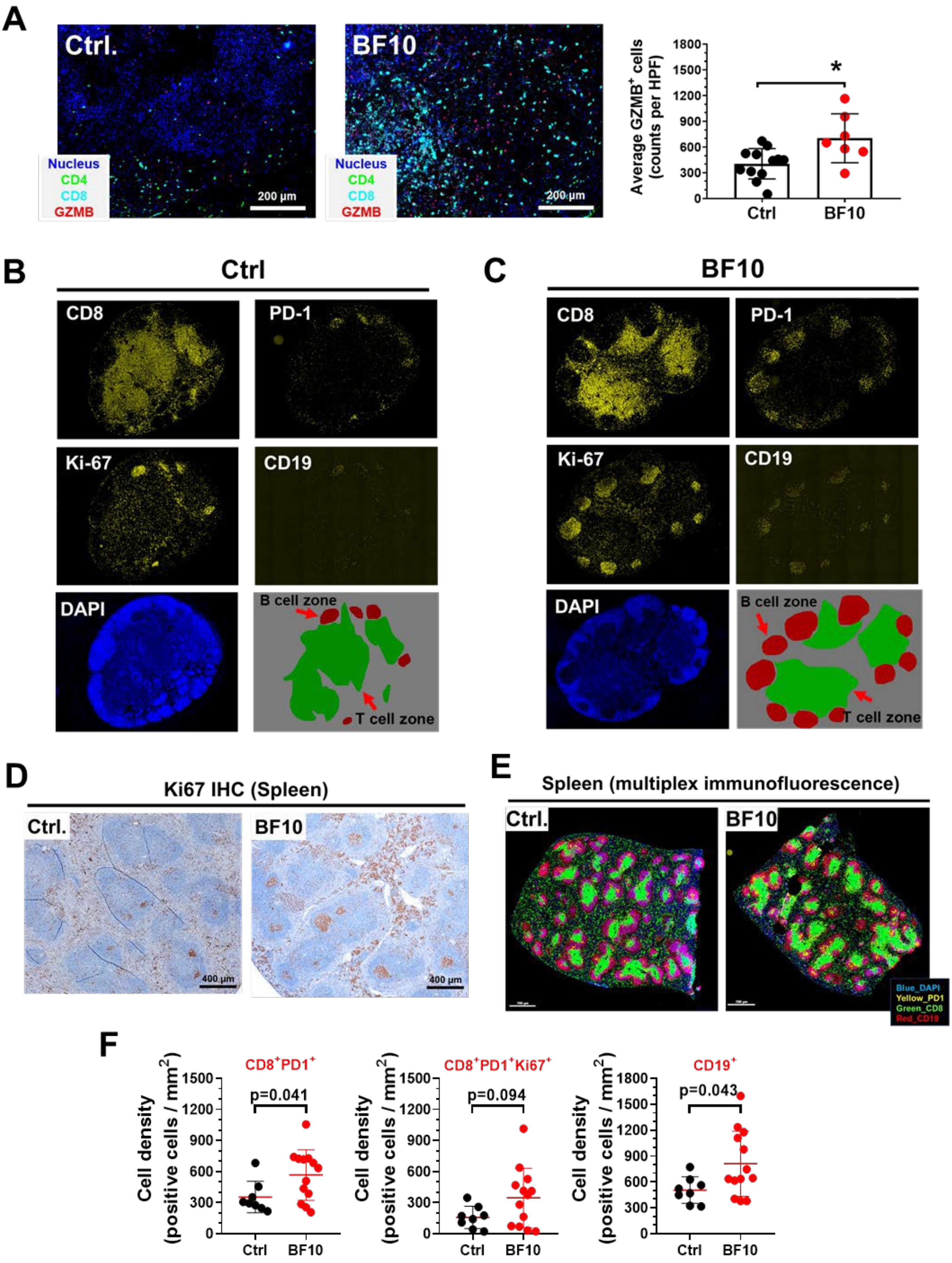

A
Ctrl.
Nucleus
CD4
CD8
GZMB
200 μm
BF10
Nucleus
CD4
CD8
GZMB
200 μm
Average GZMB+ cells (counts per HPF)
1800
1500
1200
900
600
300
0
*
Ctrl
BF10
B
Ctrl
CD8
PD-1
Ki-67
CD19
DAPI
B cell zone
T cell zone
C
BF10
CD8
PD-1
Ki-67
CD19
DAPI
B cell zone
T cell zone
D
Ki67 IHC (Spleen)
Ctrl.
BF10
400 μm
400 μm
E
Spleen (multiplex immunofluorescence)
Ctrl.
BF10
Blue_DAPI
Yellow_PD1
Green_CD8
Red_CD19
F
CD8+PD1+
p=0.041
Cell density (positive cells / mm2)
1500
1200
900
600
300
0
Ctrl
BF10
CD8+PD1+Ki67+
p=0.094
Cell density (positive cells / mm2)
1500
1200
900
600
300
0
Ctrl
BF10
CD19+
p=0.043
Cell density (positive cells / mm2)
1800
1500
1200
900
600
300
0
Ctrl
BF10

Figure S8. BF10 modulates immune cells in tumor and lymphatic organs, related to Figure 4. (A) Representative immunofluorescence images of CD4 (Green), CD8 (sky blue) and $GZMB^+$ cells (Red) within central tumor regions. All images were captured and analyzed by Vectra Polaris Imaging system and Inform AI software. The average numbers of infiltrated $GZMB^+$ T cells were counted using Inform software analysis, Scale bar, 200 µm. **(B)-(C)** Representative multiplex immunofluorescence (mIF) images of tdLN stained with immune cells (see Figure 4J). The separated color for each marker was shown as indicated. **(D)** Representative IHC staining images of Ki67-expressing cells in spleens of indicated groups. Scale bar, 400 µm. **(E)** Representative multiplex immunofluorescence (mIF) images of spleens stained with immune cells. The assayed were performed by using Opal-dye staining kit (Perkin Elmer) which contains DAPI (blue), CD8 (green), PD-1 (yellow), and CD19 (Red). Scale bar, 700 µm. **(F)** Quantification of the immune cell density of tdLN in the BF10-treated vs. control group. Images and cell density were captured and analyzed with Vectra Polaris Imaging system and Inform software. $*P < 0.05$ and $**P < 0.01$. Unpaired student's t test.

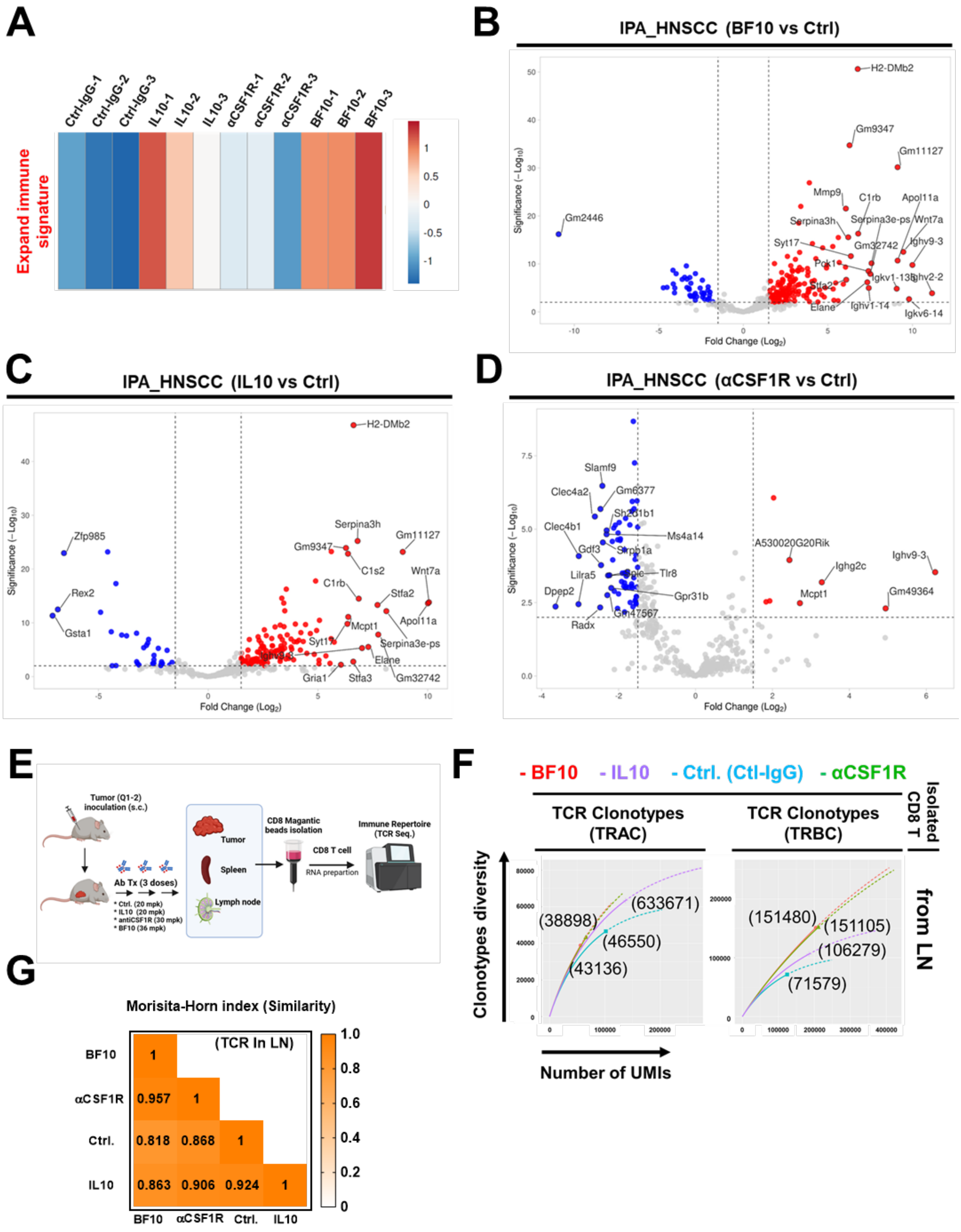

Figure S9. Transcriptomic and TCR repertoire analysis of the tumors or tdLNs treated with BF10, IL10-Fc, αCSF-1R, or control, related to Figure 5. (A) The heatmap of the expanded immune signatures in HNSCC/Q1-2 samples. Isolated tumor tissues of indicated treatment were applied to RNA extraction, followed by bulk RNA-Sequencing for gene expression profiles.

The signatures of expanded immune signature (18 genes) were analyzed by ssGSEA of gene set enrichment score for RNA-seq data (N=3 per group). **(B)-(D)** Volcano plot analysis of differential expressed genes (minus-log p-value) versus magnitude of expression change (log2 Fold Change) between pair comparison of treatments. BF10 vs. Ctrl-IgG **(B)**, IL-10 vs. Ctrl **(C)** and anti-CSF-1R vs. Ctrl **(D)**. **(E)** An illustration of mice received indicated components, followed by RNA extraction from $CD8^+$ T cells for T cell receptor (TCR) immune repertoire analysis. **(F)** T cell receptor (TCR) immune repertoire analysis of tumor-driving lymph nodes (tdLN). The clonotype diversity and distribution of both TRAC and TRBC CDR3 sequencing were assessed**.** The number of the bracket indicates observed diversity in the enrichment metrics of QIAseq-RNA Immune Repertoire Application. **(G)** Examination of the treatment-induced difference in TCR immune repertoire using Morista-Horn index.

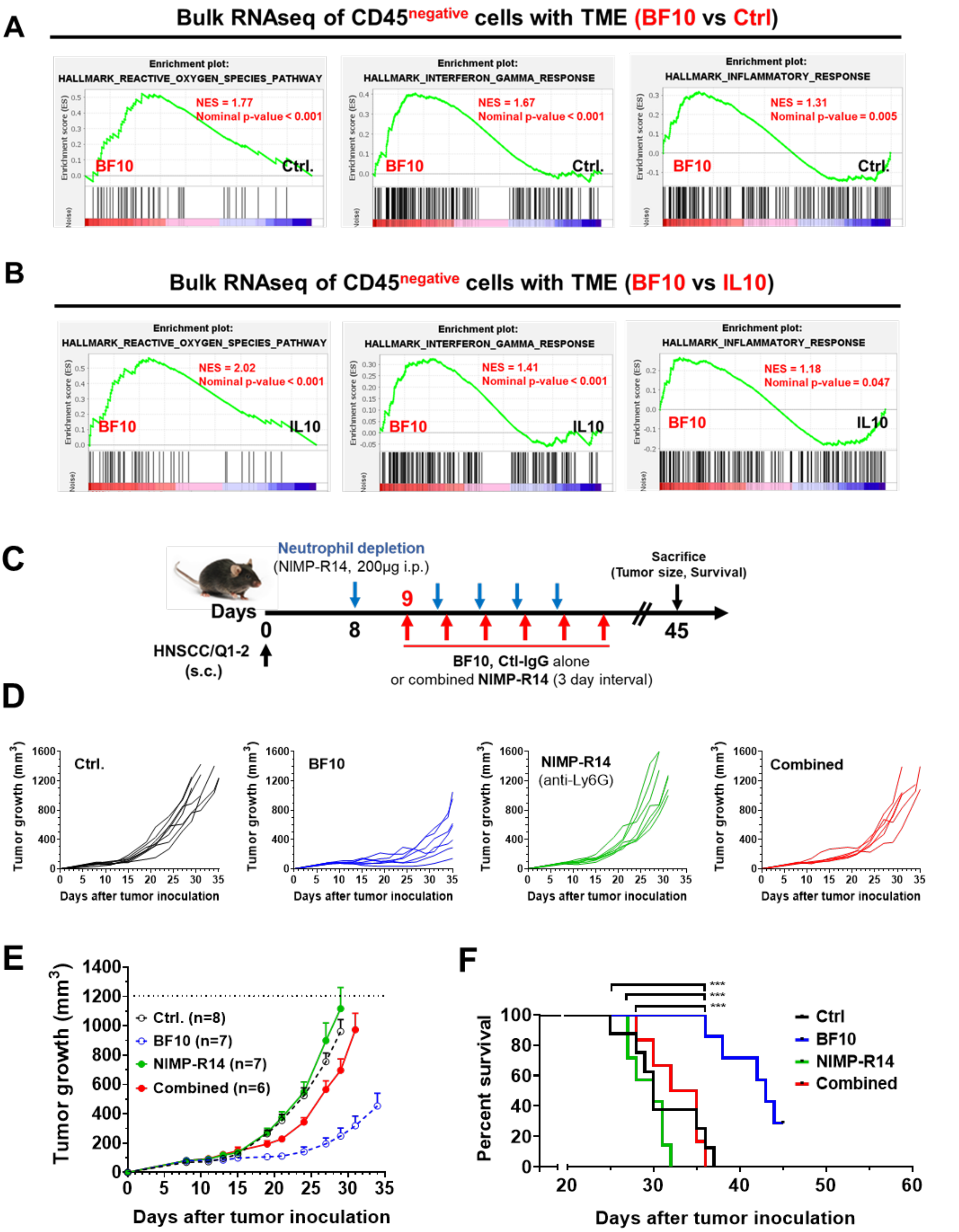

Figure S10. Gene set enrichment analysis of the bulk RNA-seq data of the $CD45^-$ cells from the four groups of mice tumors and examination of the impact of neutrophil depletion on BF10 treatment, related to Figure 6. (A)-(B). GSEA plots of BF10 versus Ctrl **(A)** and BF10 versus IL-10 **(B).** Related pathways were assessed by GSEA gene set of reactive oxygen species pathway, interferon-alpha response, interferon-gamma response, and inflammatory response signaling. The normalized enrichment

score (NES) and p-value are shown in each panel. **(C)** Experimental scheme and treatment protocol of HNSCC/Q1-2 tumor-bearing mice with neutrophil depletion. **(D)** Tumor volume curves of individual mice treated with Ctrl-IgG, BF10, NIMP-R14 or BF10 combined NIMP-R14. **(E)** The monitoring of tumor volume change in HNSCC/Q1-2 tumor-bearing mice treated with Ctrl-IgG, BF10, NIMP-R14 and BF10 combined NIMP-R14. (**F).** Survival analysis between groups of Ctrl-IgG-, BF10-, NIMP-R14- and BF10 combined NIMP-R14-treated HNSCC/Q1-2 tumor-bearing mice. P value was estimated by log-rank test.

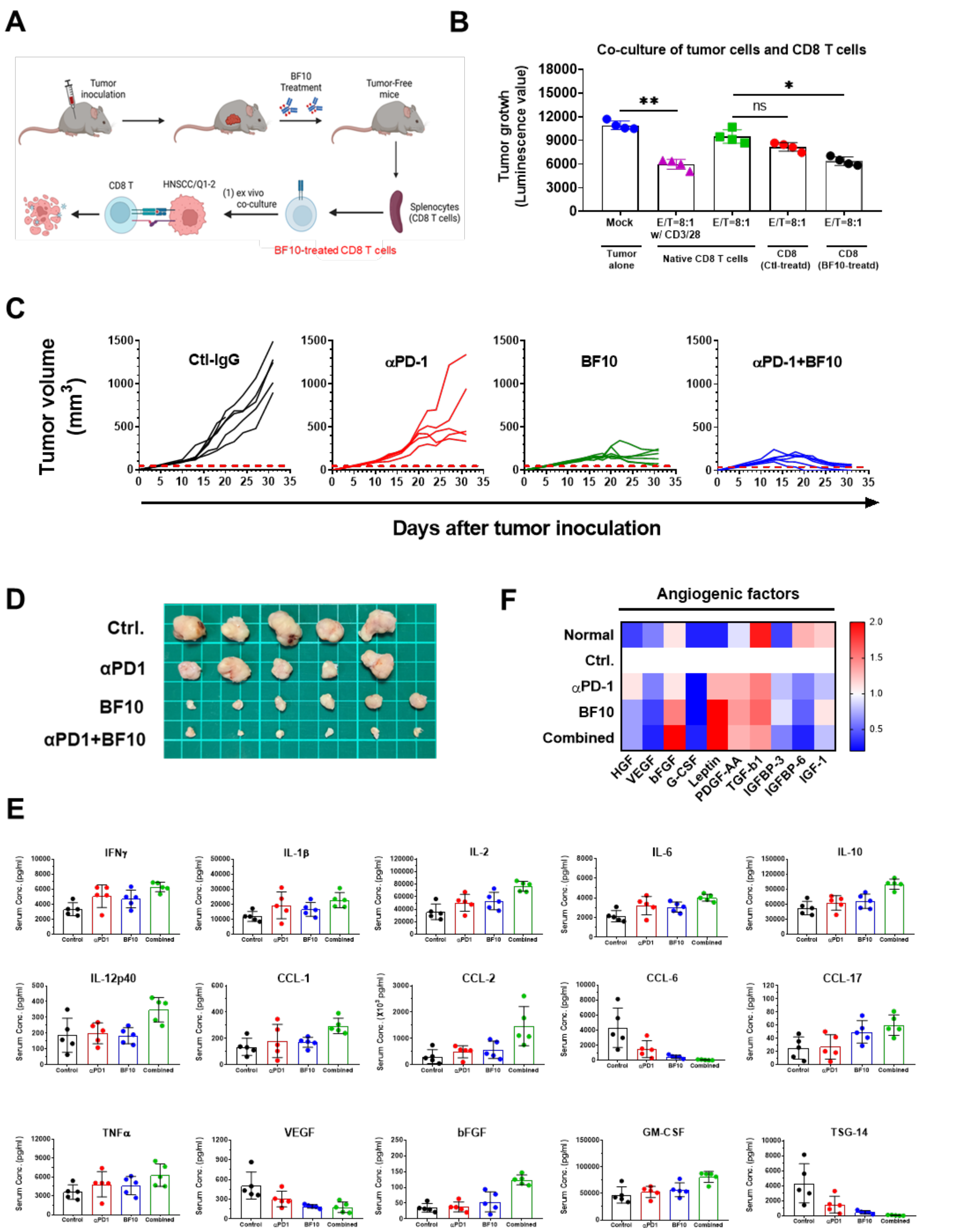

Figure S11. BF10 elicits durable protect effect and potentiates anti-PD1 efficacy, related to Figure 7. (A)-(B) Schema of the co-culture assay of CD8 T cells and tumor cells (a). HNSC/Q1-2 tumor cells (3x10^5 cells) were inoculated subcutaneously into mice, followed by BF10 treatment until tumor regression. CD8 T cells were isolated and co-cultured with tumor cells (E:T ratio = 8:1) in the presence or absence of CD3/CD28 stimulation. After 72 hours, the photos of tumor cell growth were recorded. The quantification of tumor growth inhibition of indicated groups. **(C)-(D)** The combinational efficacy of BF10 with anti-PD1

treatment in HNSC mouse model. Tumor-bearing mice (Q1-2, subcutaneous) received isotype control or anti-PD1 (200 μg) and/or BF10 (36 mg/kg) as indicated (n=5 to 6 per group). Tumor volume curves of individual mice **(C)**. Representative photos on day 31 after tumor inoculations **(D). (E)-(F)** Mouse serum from indicated treatments were analyzed by the Mouse Cytokine Antibody Array. n=4 for each group. Serum concentration of the analyzed cytokines **(E)**. A heatmap for summarizing the level of angiogenic factors **(F)**.
