# Supplementary material for: A CSF-1R-blocking antibody/IL-10 fusion protein increases anti-tumor immunity by effectuating tumor-resident CD8+ T cells
Source: Cell Rep Med. 2023 Aug 15;4(8):101154. doi: 10.1016/j.xcrm.2023.101154 (PMC10439276; doi:10.1016/j.xcrm.2023.101154)
Supplement: Document S2. Article plus supplemental information [file mmc11.pdf]

Cell Reports Medicine

Article

A CSF-1R-blocking antibody/IL-10 fusion protein increases anti-tumor immunity by effectuating tumor-resident CD8$^+$ T cells

Graphical abstract

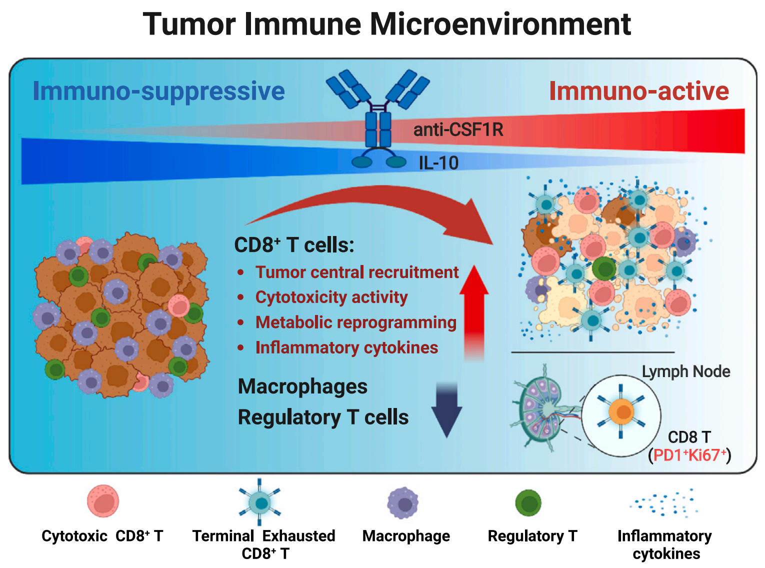

Highlights

- BF10 demonstrates efficacy against macrophage-enriched tumors
- BF10 depletes tumor-associated macrophages
- BF10 enhances cytotoxicity and metabolic reprogramming of CD8$^+$ T cells
- The combination of BF10 and αPD-1 further enhances antitumor activity

Authors

Yao-Wen Chang, Huey-Wen Hsiao, Ju-Pei Chen, ..., Ping-Chih Ho, Hung-Kai Chen, Muh-Hwa Yang

Correspondence

hkchen@elixiron.com (H.-K.C.), mhyang2@nycu.edu.tw (M.-H.Y.)

In brief

Chang et al. demonstrate the multifaceted antitumor effects of the αCSF1R-IL-10 fusion protein BF10 against head and neck cancer via activation and metabolic reprogramming of CD8$^+$ T cells and depletion of immune-suppressive macrophages and regulatory T cells. This finding leads to advancements in microenvironment-guided immunotherapy.

Chang et al., 2023, Cell Reports Medicine *4*, 101154
August 15, 2023 © 2023 The Author(s).
https://doi.org/10.1016/j.xcrm.2023.101154

CellPress

Cell Reports Medicine

CellPress
OPEN ACCESS

Article

A CSF-1R-blocking antibody/IL-10 fusion protein increases anti-tumor immunity by effectuating tumor-resident CD8$^+$ T cells

Yao-Wen Chang,[1] Huey-Wen Hsiao,[2] Ju-Pei Chen,[1] Sheue-Fen Tzeng,[3] Chin-Hsien Tsai,[3] Chun-Yi Wu,[4] Hsin-Hua Hsieh,[4] Santiago J. Carmona,[5,6] Massimo Andreatta,[5,6] Giusy Di Conza,[5,6] Mei-Tzu Su,[7] Pandelakis A. Koni,[2] Ping-Chih Ho,[5,6] Hung-Kai Chen,[2,*] and Muh-Hwa Yang[1,7,8,9,10,11,*]

[1]Cancer and Immunology Research Center, National Yang Ming Chiao Tung University, Taipei 11221, Taiwan
[2]Elixiron Immunotherapeutics (Hong Kong) Ltd., Hong Kong
[3]Graduate Institute of Life Sciences, National Defense Medical Center, Taipei 11221, Taiwan
[4]Department of Biomedical Imaging and Radiological Sciences, National Yang Ming Chiao Tung University, Taipei 11221, Taiwan
[5]Department of Oncology, University of Lausanne, Lausanne, Switzerland
[6]Ludwig Institute for Cancer Research at University of Lausanne, Lausanne, Switzerland
[7]Department of Biotechnology and Laboratory Science in Medicine, National Yang Ming Chiao Tung University, Taipei 11221, Taiwan
[8]Institute of Clinical Medicine, National Yang Ming Chiao Tung University, Taipei 11221, Taiwan
[9]Department of Oncology, Taipei Veterans General Hospital, Taipei 11217, Taiwan
[10]Department of Teaching and Research, Taipei City Hospital, Taipei, Taiwan
[11]Lead contact
*Correspondence: hkchen@elixiron.com (H.-K.C.), mhyang2@nycu.edu.tw (M.-H.Y.)
https://doi.org/10.1016/j.xcrm.2023.101154

SUMMARY

Strategies to increase intratumoral concentrations of an anticancer agent are desirable to optimize its therapeutic potential when said agent is efficacious primarily within a tumor but also have significant systemic side effects. Here, we generate a bifunctional protein by fusing interleukin-10 (IL-10) to a colony-stimulating factor-1 receptor (CSF-1R)-blocking antibody. The fusion protein demonstrates significant antitumor activity in multiple cancer models, especially head and neck cancer. Moreover, this bifunctional protein not only leads to the anticipated reduction in tumor-associated macrophages but also triggers proliferation, activation, and metabolic reprogramming of CD8$^+$ T cells. Furthermore, it extends the clonotype diversity of tumor-infiltrated T cells and shifts the tumor microenvironment (TME) to an immune-active state. This study suggests an efficient strategy for designing immunotherapeutic agents by fusing a potent immunostimulatory molecule to an antibody targeting TME-enriched factors.

INTRODUCTION

Tumor-associated macrophages (TAMs) are one of the most abundant tumor-infiltrated immune cell types that create an immunosuppressive tumor microenvironment (TME) to repress antitumor immunity[1,2] and facilitate metastatic colonization.[3–5] Increased infiltration of TAMs is associated with a worse prognosis of patients with cancer.[6–8] TAMs are therefore considered to be a prime target in the TME over the past decade, with extensive approaches aimed at eliminating or repolarizing TAMs to remodel the TME.[9–12] A major strategy for targeting TAMs is to block the colony-stimulating factor-1 receptor (CSF-1R), either by using monoclonal antibodies that prevent ligand (CSF-1 and IL-34) binding or small-molecule inhibitors that prevent downstream signaling.[13–15] However, the results of CSF-1R inhibitors/antibodies have been relatively disappointing to date, with no significant benefit in most anticancer clinical trials.[16–18] Thus, a means to overcome the hurdles to more effective cancer therapy presented by TAMs remains an urgent but unmet medical need.

Interleukin-10 (IL-10) has long been recognized as an anti-inflammatory mediator through inhibition of antigen-presenting cells (APCs).[19] However, the pleiotropic nature of IL-10 is now well established, especially with regard to its antitumor effects.[20,21] IL-10 exerts antitumor activity by directly activating the IL-10 receptor (IL-10R) on CD8$^+$ T cells and natural killer (NK) cells.[22–25] IL-10 prevents dendritic cell (DC)-mediated apoptosis of tumor-specific CD8$^+$ T cells through IL-10R signaling on DCs.[26] We recently showed that IL-10 stimulates the oxidative phosphorylation of terminally exhausted T cells to reinvigorate their proliferation and antitumor activity.[27,28] In consideration of the therapeutic application of IL-10, a half-life-extended form of IL-10 (pegylated IL-10) demonstrated effective tumor control in mouse models.[29] Pegylated IL-10 was well tolerated in cancer clinical trials, which showed induction of CD8$^+$ T cell immunity.[22,23] However, the short half-life of pegylated IL-10 has limited its application. Another form of half-life-extended IL-10 includes an immunoglobulin Fc domain fusion protein (IL-10-Fc), which acts directly on CD8$^+$ terminally exhausted T cells to reprogram metabolic activities, restoring their

This is an open access article under the CC BY license (http://creativecommons.org/licenses/by/4.0/).

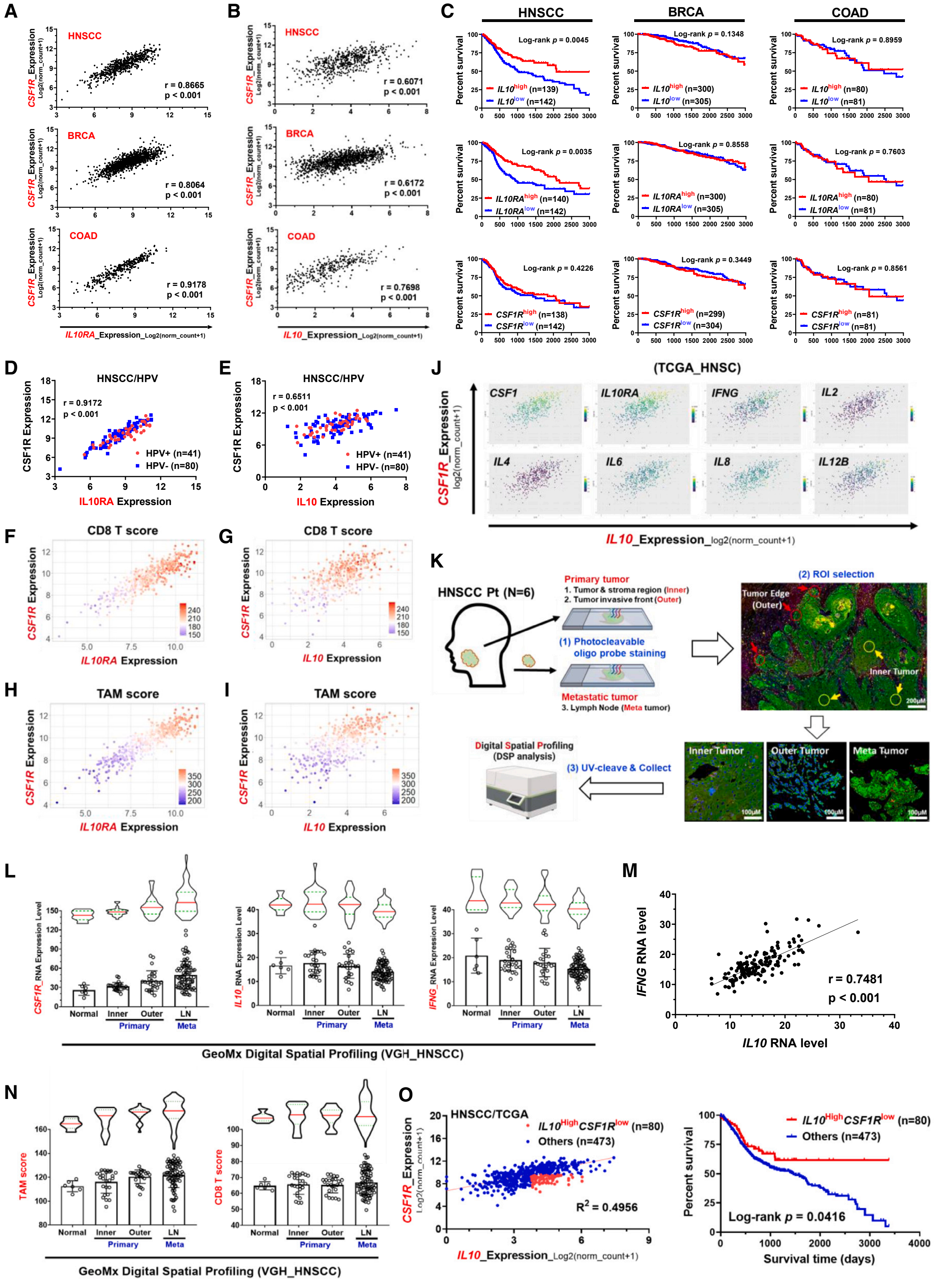

(legend on next page)

CellPress
OPEN ACCESS

proliferation and antitumor activity in mouse tumor models.[28] Despite the extended half-life of IL-10-Fc, peri-tumoral administration of the IL-10-Fc was employed to minimize adverse systemic effects.[28] Developing an optimal form of IL-10 that can be effectively delivered to the TME with minimal systemic side effects is the major challenge of the clinical application of IL-10 therapy.

Recently, emerging evidence has revealed the spatial association between APCs and T cells in the TME. Tumor-specific CD8$^+$ T cells are closely associated with APCs in ovarian cancer.[30] The spatiotemporal codependency between CD8$^+$ exhausted cells and TAMs was also noted, and the depletion of TAMs was found to reinvigorate the effector potential of the exhausted T cells.[31] Furthermore, macrophage-binding proteins can be delivered either though binding to peripheral macrophages first or to already tumor-resident macrophages.[32] In the present study, we generated an anti-CSF1R antibody-IL-10 fusion protein based on the hypothesis that this will result in better delivery of IL-10 to the TME and thereby result in greater rejuvenation of terminally exhausted T cells in TAM-enriched tumors. Our positive results highlight the potential for the development of such an antibody-IL-10 fusion protein as a promising strategy against TAM-enriched cancers.

RESULTS

An *IL-10*^{high}/*CSF1R*^{low} profile correlates with an activated immune signature and favorable prognosis in head and neck cancer

Based on the promising antitumor effects of IL-10,[20,21,28] we aimed to develop an IL-10-based fusion protein for anticancer treatment. The potential of the spatiotemporal correlation between macrophages and T cells[30,31] prompted us to focus on macrophages as a target to deliver IL-10 to intratumoral T cells. Among the different types of tumors, macrophages were most abundant in head and neck squamous cell carcinoma (HNSCC), breast cancer (BRCA), colorectal adenocarcinoma (COAD), lung adenocarcinoma (LUAD), lung squamous cell carcinoma (LUCC), liver hepatocellular cancer (LIHC), and skin cutaneous melanoma (SKCM) (Figure S1A; Table S1). Intriguingly, we observed a significant correlation of the expression of the *CSF1R* and *IL-10-IL-10RA* axis in HNSCC, BRCA, and COAD of The Cancer Genome Atlas (TCGA) cohort (Figures 1A, 1B, and S1B). However, the prognostic impact of the *IL-10-IL-10RA* axis was shown in HNSCC, i.e., a high expression of either *IL-10* or *IL-10RA* correlated with a better outcome in patients with HNSCC but not in patients with BRCA or COAD. The expression of *CSF1R* had no prognostic impact in all three types of patients with cancer (Figure 1C). We herein focused more on HNSCC, where responses to current immunotherapies had potential room for improvement.[33,34] The positive correlation between the expression of the *IL-10-IL-10RA* axis and *CSF1R* was validated in the RNA sequencing data of HNSCC from Taipei Veterans General Hospital (TVGH) (Figures S1C and S1D; Table S2). In HNSCC, human papillomavirus (HPV)$^+$ cases display distinct clinical characteristics compared with non-HPV-infected patients.[35] We categorized TCGA HNSCC cases into HPV$^+$ and HPV$^-$ ones. The results revealed that HPV positivity did not influence the correlation between *CSF1R* and the *IL-10-IL-10RA* axis (Figures 1D and 1E).

We next examined the correlation of *IL-10/IL-10RA/CSF1R* and the expression of cytokine genes and immune cell signatures in HNSCC. First, we analyzed the correlation between *IL-10/IL-10RA/CSF1R* expression and CD8$^+$ T cell score and TAM score in HNSCC. Coexpression of *IL-10/IL-10RA* and *CSF1R* was associated with a higher CD8$^+$ T cell score and TAM score in both TCGA and TVGH cohorts (Figures 1F–1I and S1E–S11H). An increased expression of the cytokine genes *IFNG*, *IL-2*, *IL-6*, and *IL-12B* was noted in the *CSF1R*^{high}*IL-10*^{high}/*CSF1R*^{high}*IL-10RA*^{high} patients (Figures 1J and S1I). We next examined the gene expression profiles from another set of TVGH HNSCC samples analyzed by the spatial transcriptomics technology Digital Spatial Profiler (DSP) (Tables S3A and S3B). The regions of interest (ROIs) were categorized as normal counterparts, inner tumors, outer invasive fronts, and metastatic tumors by a pathologist to represent their different levels of aggressiveness (Figure 1K). A gradually increased *CSF1R* from the inner tumors to the invasive fronts to the metastatic tumors was seen, whereas a trend of a decrease in *IL-10* and *IFNG* was noted from the inner tumors to the invasive fronts to the metastatic tumors (Figure 1L). A significant correlation

Figure 1. Clinical relevance of the expression levels of IL-10 and CSF-1R in patients with cancer

(A and B) The correlation of RNA level between IL-10/IL-10-RA axis and CSF-1R of head and neck squamous carcinoma (HNSCC; N = 566), breast cancer (BRCA; N = 1,218), and colon adenocarcinoma (COAD; N = 329) in The Cancer Genome Atlas (TCGA) database. The correlation was determined by Pearson correlation (r).

(C) Kaplan-Meier survival analysis of HNSCC-, BRCA-, or COAD- patients with different expression profiles of *IL-10*, *IL-10RA*, and *CSF1R*. High, upper quartile; low, lower quartile. p values were estimated by log-rank test.

(D and E) The correlation of RNA level between IL-10/IL-10-RA axis and HPV positivity in TCGA-HNSCC cases. The correlation was determined by Pearson correlation (r).

(F–I) 3D scatterplots for analyzing the expression of CD8$^+$ T score and tumor-associated macrophage (TAM) score related to IL-10/IL-10RA axis and CSF1R in TCGA-HNSCC. CD8$^+$ T or TAM scores were expressed from blue to red gradient color dots (low to high). The value expressed by RNA level (log2(norm_count+1)).

(J) 3D scatterplots for the expression of different cytokine genes related to *IL-10* and *CSF1R* in TCGA-HNSCC.

(K) Schematic outlines represent the approach of digital spatial profiling (DSP) analysis of human HNSCC (14 slides with 171 regions of interest [ROIs] analyzed from 6 patients). ROIs were categorized as peri-normal tissue (normal), primary tumor (inner tumor and outer tumor), and metastatic lymph node (LN). Scale bar, 100 or 200 μm as indicated.

(L) DSP analysis of *CSF1R*, *IL-10*, and *IFNG* in ROIs of HNSCC samples. The ROIs (500 μm in diameter) were analyzed using a DSP CTA (Cancer Transcriptome Atlas) panel with 1825 RNA-binding oligonucleotide probes.

(M) Correlation between expression levels of *IL-10* and *IFNG* from DSP data. The correlation was determined by Pearson correlation (r).

(N) DSP analysis of the TAM score and CD8$^+$ T score related to tumor spatial location in ROIs of HNSCC samples.

(O) Left, a scatterplot of *IL-10* and *CSF1R* gene expression levels in TCGA HNSCC patients. Red dots indicate the patients with the top 50% of *IL-10* (IL-10^{high}) and the bottom 50% of *CSF1R* (CSF1R^{low}). Right, survival analysis between groups of IL-10^{high}CSF1R^{low} (red line) and others (blue line). p value was estimated by a log-rank test.

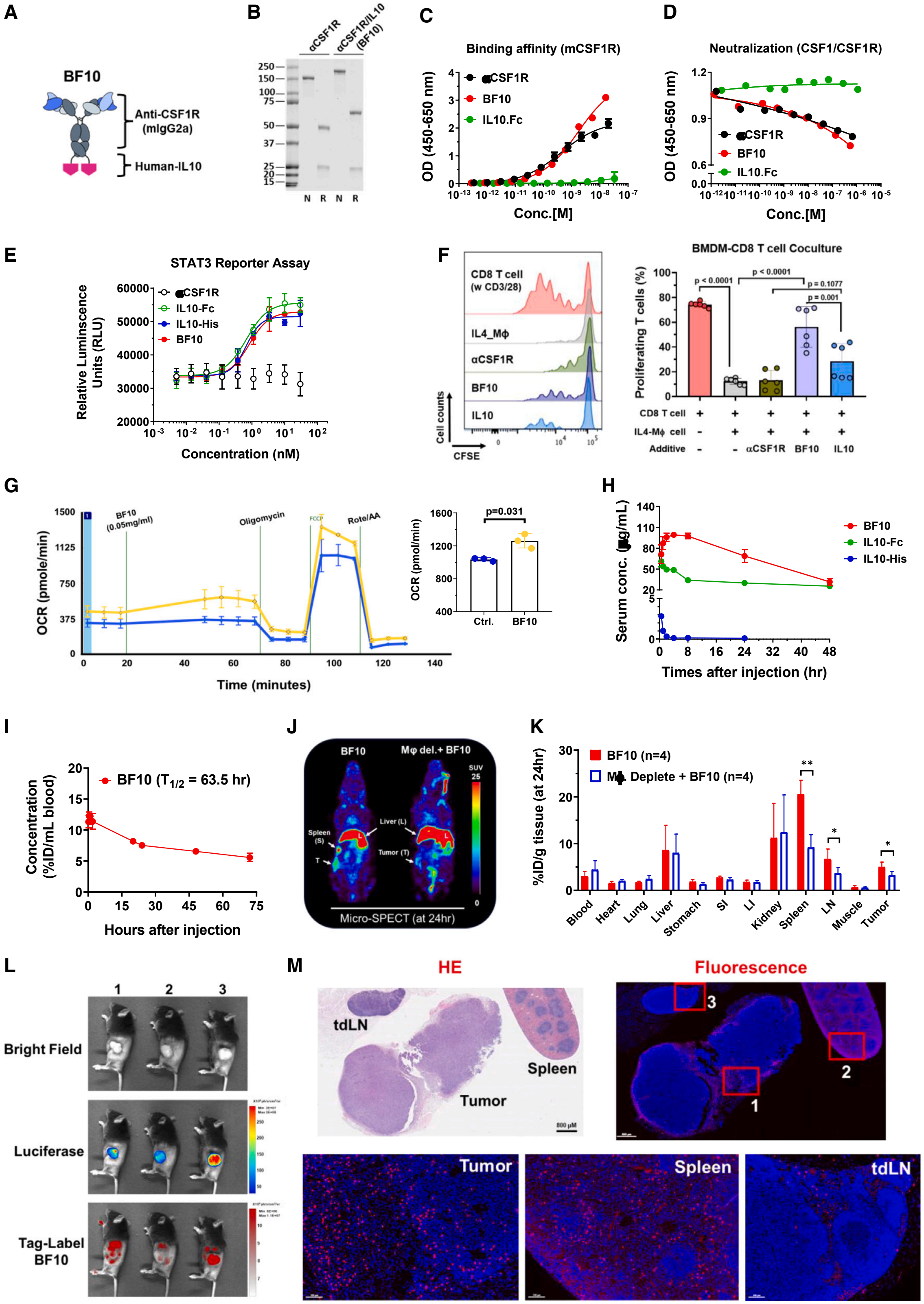

(legend on next page)

CellPress
OPEN ACCESS

between *IFNG* and *IL-10* was demonstrated in the analyzed ROIs (Figure 1M). To investigate the correlation between TAM and CD8$^+$ T cells in DSP data, a gradual increase of the TAM score was noted from non-invasive inner tumors to the invasive/metastatic tumors; nevertheless, the change of CD8$^+$ T cell score among different regions was not significant (Figure 1N). A significant correlation between TAM score and CD8$^+$ T cell score was demonstrated (Figure S1J). The spatial transcriptomics result indicated that expression of *IL-10/IL-10RA* was associated with less invasiveness and an activated immune signature, whereas *CSF1R*/TAM score correlated with aggressiveness in HNSCC. Last, we analyzed the prognostic impact of *IL-10-CSF1R* in patients with HNSCC. A subgroup of patients with HNSCC with the *IL-10*^{high}*CSF1R*^{low} profile had a favorable outcome (Figure 1O); on the contrary, *IL-10*^{high} *CSF1R*^{high} did not have prognostic impact (Figure S1K). Taken together, the clinical data suggest a correlation of the expression of the *IL-10/IL-10RA* axis and *CSF1R* and an association of the *IL-10*^{high}*CSF1R*^{low} profile with an immunostimulatory signature and favorable prognosis of patients with HNSCC. This finding implicates a potential therapeutic impact of the stimulation of the IL-10/IL-10R axis in combination with the blockade of CSF-1R for tumors with abundant macrophages and T cells.

The anti-CSF1R-IL-10 fusion protein BF10 harbors the bioactivity of both IL-10 and anti-CSF1R

Based on the above clinical analysis, we generated anti-CSF1R-IL-10 antibody fusion proteins for subsequently experiments. The bifunctional anti-CSF-1R-IL-10 fusion protein (named BF10) consists of a murine immunoglobulin G2a (IgG2a) isotype anti-mCSF-1R antibody with a human IL-10 monomer fused to the heavy-chain C terminus via a linker sequence (Figure 2A). The fusion protein was generated based on the fact that human IL-10 could cross-react with mouse IL-10R[28,36] and that the bioactivity of the IL-10 IgG fusion (IL-10-Fc) has been approved.[28] SDS-PAGE was performed to validate the molecular weight of BF10. A higher molecular weight of the heavy chain of BF10 (~65 kDa) compared with the original anti-CSF1R antibody (αCSF1R) heavy chain (~50 kDa) was evident, while the molecular weight of light chains was the same (~25 kDa) in BF10 and α-mCSF-1R (Figure 2B). BF10 harbored a similar binding affinity (Figure 2C) and neutralization capacity (Figure 2D) to mCSF-1R compared with α-mCSF-1R. The binding abilities of BF10 to mIL-10Rα and hIL-10-Rα were validated (Figures S2A–S22G).

We next examined the *in vitro* bioactivity of BF10. BF10 had a comparable ability to suppress CSF1-dependent proliferation of murine M-NFS-60 cells (Figure S2H). BF10 stimulated a similar degree of *STAT3* promoter activity in IL-10Rα-expressing HeLa cells as IL-10 did (Figure 2E). Next, we performed macrophage-CD8$^+$ T cell coculture assays to examine whether BF10 treatment could sustain T cell proliferation. CD3/28-stimulated CD8$^+$ T cells were cocultured with IL4-primed macrophages and were treated with either IL-10-Fc, α-mCSF-1R, BF10, or a control IgG. As expected, IL-4-primed macrophages suppressed T cell proliferation, and anti-mCSF1R antibody did not reverse this macrophage-mediated suppression of T cell proliferation. In contrast, both IL-10-Fc and BF10 effectively rescued

Figure 2. Generation and characterization of the bifunctional anti-CSF-1R-IL-10 fusion proteins

(A) Schematic representation of the bifunctional anti-CSF-1R-IL-10 fusion protein (BF10). BF10 was designed by genetically fusing human IL-10 to the C terminus of the CSF-1R fragment (mouse IgG2a) separated by a 14 amino acid linker.

(B) SDS-PAGE of BF10 and the anti-CSF-1R antibody. N, non-reducing; R, reducing.

(C) Dose-dependent binding affinity of anti-CSF-1R Ab, BF10, and IL-10-Fc to recombinant mouse CSF-1R protein by ELISA. Calculated curve of one representative data of three independent experiments. Each point (OD value) was expressed as the average of two repeats and was presented as mean ± SD.

(D) The competition activity of anti-CSF-1R, BF10, and IL-10-Fc. Serial dilutions of the test proteins were preloaded into the well before mouse CSF-1 to CSF-1R binding reaction. The values were measured by absorbance and expressed as an OD value (450–650 nm). Calculated curve of one representative data of three independent experiments was shown.

(E) STAT3 reporter assay. IL-10RA-overexpressed HeLa cells were seeded into a 96-well plate with complete DMEM medium (10% FBS) at 37°C for 4 h. Serial dilutions of IL-10-His tag, IL-10-Fc, anti-CSF1R, and BF10 were added into well for 18 h. Luciferase activity was measured using ONE-Glo Luciferase Assay (Promega). The calculated curve of one representative data of three independent experiments was presented as mean ± SD.

(F) Effects of BF10 on T cell proliferation. CFSE-labeled CD8$^+$ T cells were activated with anti-CD3 and anti-CD28 in absence or presence of IL-4-primed BMDMs. 50 ng/mL anti-CSF-1R, IL-10, or BF10 was added as indicated. Data are cumulative results of two independent experiments (n = 6 per group). The results were shown as mean ± SD, and the statistics were calculated using one-way ANOVA with Tukey test.

(G) Oxygen consumption rate (OCR) of CD8$^+$ T cells with BF10 treatment. Isolated splenic CD8$^+$ T cells from tumor-bearing mice were pretreated with BF10 (0.05 mg/mL) for 30 min and then sequential addition of the Seahorse assay reagents of oligomycin (1 μM), FCCP (0.5 μM), and Rot/antimycin A (1 μM) to examine the effect of BF10 on T cell metabolism. Representative curve is one of three independent experiments and n = 3 repeats per each assay. p value was calculated using Student's t test.

(H) The serum concentration of BF10. Mice received the equivalent dose treatments of BF10 (20 mg/kg), IL-10-Fc (10 mg/kg), or IL-10-tag (5 mg/kg) using intravenous injection. 10 μL blood was diluted and collected as the indicated times (0.25, 1, 2, 4, 8, 24, 48, and 72 h) and was subjected to ELISA to detect the IL-10 concentration.

(I) The biodistribution of ^{111}In-BF10. Tumor-bearing mice with or without clodronate-liposome before BF10 injection. After 24 h, tissue samples were excised and weighted. The radioactivity of the sample was measured, and the value was expressed as a percentage of the injected dose per gram of sample (%ID/g).

(J) The SPECT images of HNSCC/Q1-2 tumor-bearing mice receiving radiolabeled ^{111}In-BF10 injection. Mice were imaged at 24 h after injection. T, tumor; S, spleen; L, liver.

(K) The blood clearance kinetic of ^{111}In-BF10. ^{111}In-BF10 were intravenously (i.v.) injected, and blood samples were collected at the indicated time points. Data were expressed as the percentage injected dose per milliliter (%ID/mL). The half-life ($T_{1/2}$) of the curve was estimated by Prism (v.9.2.0). p values were calculated using Student's t test. *$p < 0.05$ and **$p < 0.01$.

(L and M) Representative images of the biodistribution of BF10. HNSC/Q1-2^{Luciferase} tumor-bearing mice were i.v. injected with Vivo680Tag-labeled BF10 (150μg) for 16 h before *in vivo* bioluminescence detection (L), and subsequently, tissues (tumor, spleen, or tdLNs) were isolated for fluorescence-Vivo680Tag detection (M). Scale bar, 100 or 800 μm as indicated.

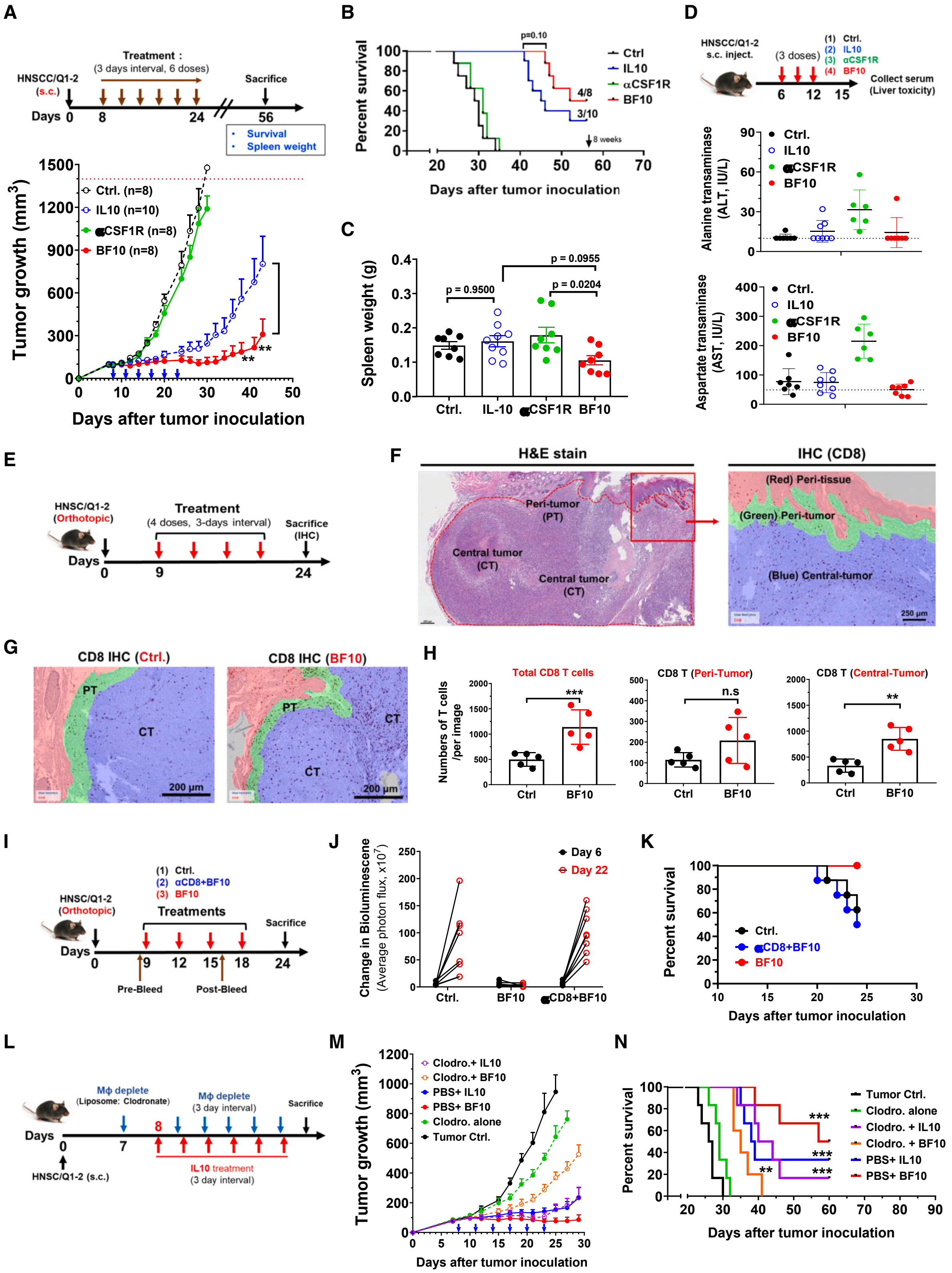

(legend on next page)

T cell proliferation, and BF10 outperformed IL-10-Fc (Figures 2F and S2I). We further generated two clones of fusion proteins consisting of human αCSF-1R (α-hCSF-1R) and IL-10-Fc to validate the efficacy of αCSF-1R-IL-10 on human immune cells (Figure S2J). The fusion protein (huAb) reduced the viability of human macrophages (Figure S2K) and increased the secretion of interferon γ (IFN-γ) and granzyme B by human activated $CD8^+$ T cells (Figure S2L). We examined the metabolic profiling of $CD8^+$ cells treated with the BF10 *ex vivo* by an Agilent Seahorse analyzer because IL-10 induces metabolic reprogramming of T cells.[28] BF10 increased the oxygen consumption rate of the splenic $CD8^+$ T cells (Figure 2G). This result suggests that BF10 elicits a similar effect on metabolic reprogramming on $CD8^+$ T cells as IL-10Fc did in the previous study.

We investigated the pharmacokinetic of BF10, IL-10-Fc, and IL-10-His *in vivo* by examination of the serum concentration of BF10 at different time points by ELISA. Both IL-10-Fc and BF10 had a longer, better half-life than IL-10-His. Interestingly, different patterns of serum concentration changes were noted between BF10 and IL-10-Fc (Figure 2H). We next examined the tumor targeting and tissue biodistribution of BF10 by intravenous injection of radiolabeled BF10 (^{111}In-BF10) into tumor-bearing mice. The serum concentration of BF10 first validated in non-tumor-bearing mice with a half-life ($T_{1/2}$) around 63.5 h (Figure 2I). Micro-single-photon emission computed tomography (SPECT)/CT images demonstrated an obvious uptake of ^{111}In-BF10 in spleen, liver, lymph node, and tumor tissues. Depletion of macrophage significantly reduced BF10 accumulation in spleen, tumor, and lymph nodes (Figures 2J and 2K). We further applied another bioluminescence assay to confirm the binding capability of BF10 *in vivo*. We labeled BF10 with a near-infrared (NIR) fluorochrome, VivoTag. Labeled BF10 was intraperitoneally injected into mice bearing luciferase-expressing syngeneic murine HNSCC tumors, and mice were analyzed by IVIS imaging 16 h later, followed by collection of tumors, tumor-draining lymph nodes (tdLNs), and spleens for analysis. A combination of luciferase bioluminescence and Vivo Tag fluorescence imaging showed that BF10 accumulated in tumors as well as sporadic areas within the peritoneal cavity (Figure 2L). The VivoTag fluorescence images within the tissues confirmed the distribution of BF10 to tumors, tdLNs, and spleens (Figure 2M). In summary, these experiments confirm the bioactivity and specificity of the αCSF1R-IL-10 fusion proteins.

The subcomponents IL-10 and α-mCSF1R both construct the antitumor activity of BF10

We examined the antitumor effect of BF10 in syngeneic murine tumor models. We first applied the mouse syngeneic cancer platform (MuScreen, CrownBio), and a total of 9 cancer cell lines were utilized. Suppression of tumor growth was observed in most types of cancers with the exception of prostate cancer (RM-1), albeit with a somewhat limited effect in Pan02 (pancreatic cancer) and lung cancer (B16BL6) (Figures S3A and S3B). There was a positive correlation between tumor growth inhibition and the percentage of estimated macrophages (Figure S3C), implying the potential of a better efficacy of BF10 on macrophage-enriched tumors. We subsequently focused on the antitumor activities of BF10 in HNSCC owing to the evidence from clinical sample analyses (Figure 1) and the high abundance of TAMs[37–39] and relative immune responsiveness of HNSCC.[40,41] Abundant infiltrated macrophages were found within the tumor microenvironment of the murine HNSCC syngeneic model, and the relative tumor growth inhibition (TGI) by BF10 is about 68.5% (Figures S3D and S3E). This result justified the selection of HNSCC as a candidate type of cancer for BF10 treatment. The antitumor efficacy of BF10 was validated in the subcutaneous tumor model formed

Figure 3. BF10 suppresses tumor growth via increasing tumor-infiltrating $CD8^+$ T cells and TAM depletion

(A–C) Experimental scheme. The HNSCC/Q1-$2^{Luciferase}$ tumor cells (5×10^5 cells) were subcutaneously inoculated into mice and received treatments of control (Ctrl)-IgG (30 mg/kg), IL-10-Fc (20 mg/kg), αCSF1R (30 mg/kg), or BF10 (36 mg/kg) for six doses as indicated. All mice received the equivalent dose treatment according to different molecular weights of antibody components as described in the STAR Methods. After 8 weeks, tumor growth curve (A), survival proportion (B), and spleen weight (C) were recorded and analyzed. The survival analysis was defined as death spontaneously within 1 month or a tumor burden size reaching 1,500 mm^3 or a length >1.5 cm. Statistical analyses were evaluated using one-way ANOVA with post-hoc Tukey test. The survival was analyzed by Kaplan-Meier method and a log rank test. $^{**}p < 0.01$.

(D) Experimental scheme for assessing liver toxicity. Mice received a three-dose treatment as indicated and were sacrificed at day 15. Mouse serum was collected and examined for the levels of ALT and AST. The quantified results were presented as mean ± SD, and these statistics were calculated using unpaired Student's t test (two groups comparison) and one-way ANOVA (more than 3 groups) with Tukey test. $^{**}p < 0.01$.

(E and F) The experimental schema with BF10 treatment (E) and an illustration of different tumor regions (F). The orthotopic HNSCC tumor tissues were collected for staining. Tumor tissue was separated into 3 regions, which are defined as peri-tumor (PT; green), central-tumor (CT; blue), and peri-normal tissue (red). Scale bar, 250 μm.

(G) Representative images of the distribution of tumor-infiltrated $CD8^+$ T cells with BF10 treatment.

(H) Quantitative results of tumor-infiltrated $CD8^+$ T cells within tumor tissues. Images were captured and analyzed by the Vectra Polaris Imaging System and Inform software. The average numbers of the $CD8^+$ T cells were counted and expressed per field (1,397 × 1,048 μm^2) using Inform software analysis. p values were calculated using Student's t test. $^{**}p < 0.01$ and $^{***}p < 0.001$.

(I) Experimental scheme of $CD8^+$ T cell depletion by administration with indicated treatments in orthotopic HNSCC tumor model. $CD8^+$ T cell depletion was carried out using 200 μg anti-CD8 antibody (i.p. every 3 days) simultaneously with or without BF10.

(J) Bioluminescence intensity measured and quantified using Xenogen IVIS 100 imaging system on days 6 and 22 for indicating the tumor growth.

(K) The Kaplan-Meier survival curve of the tumor-bearing mice receiving control (n = 8), BF10 (n = 8), and anti-CD8 plus BF10 (n = 7). The death of mice was recorded when they died spontaneously, had a tumor diameter of 1.5 cm, or had their eating behavior affected at day 24.

(L) Scheme of the macrophage depletion experiment. Tumor-bearing mice were treated with clodronate-liposome (200 μL per 20 g body weight), followed by cotreatment of IL-10-Fc or BF10 with a 3 day interval for following tumor growth and survival.

(M and N) Tumor-bearing mice (N = 6–7 per group) received the treatments as indicated. Tumor size was measured by digital caliper, and tumor volume was calculated using the formula V = (length × width × width)/2. Values represent tumor growth curve in all groups (M). The survival was analyzed by Kaplan-Meier method and a log-rank test (N). $^{**}p < 0.01$ and $^{***}p < 0.001$.

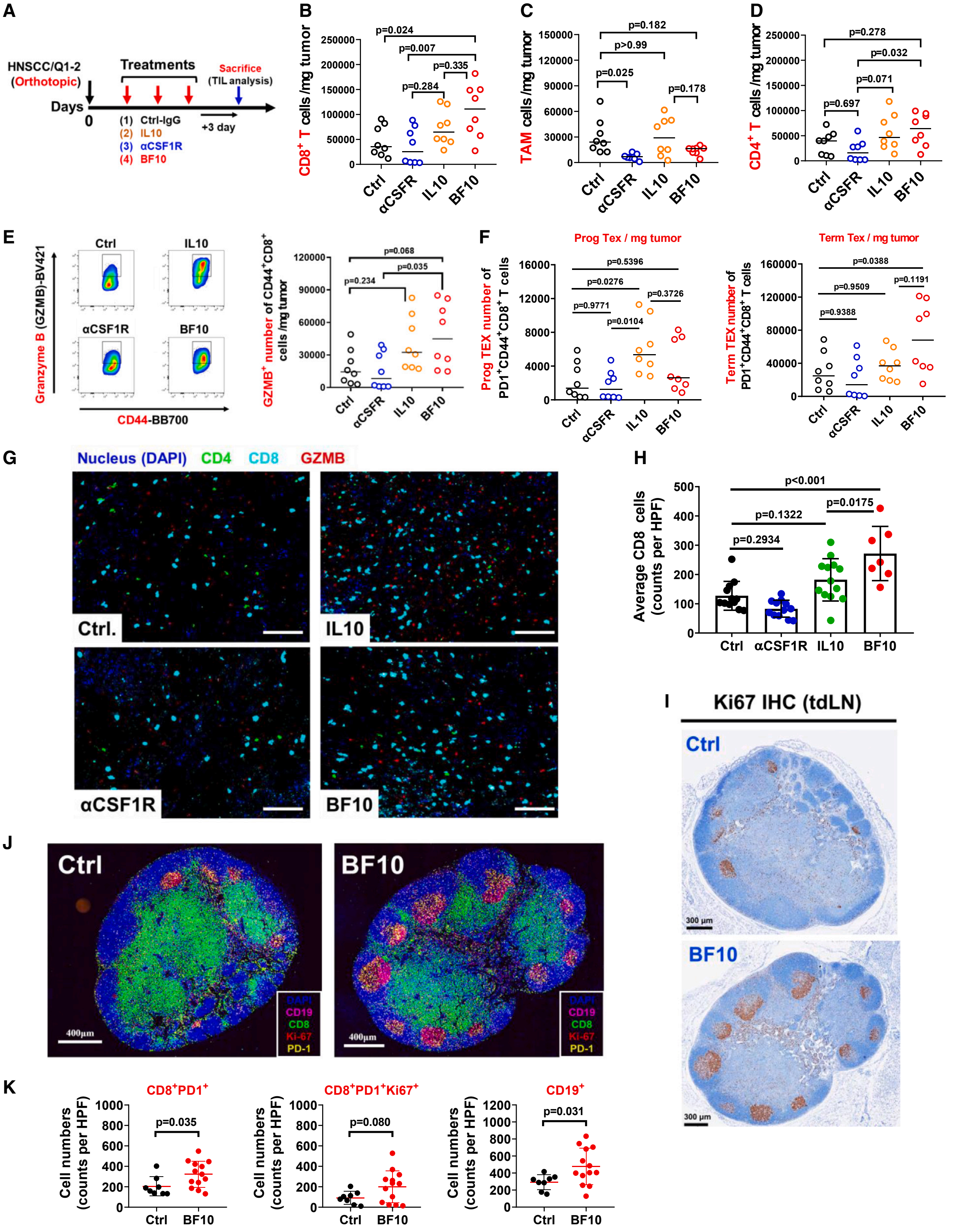

(legend on next page)

from the syngeneic murine HNSCC cell line MTCQ1-2 (Figures S4A–S4D). We next examined whether BF10 harbored a better antitumor efficacy compared with its subcomponents (IL-10 and α-mCSF1R) in the subcutaneous HNSCC model. With the treatment of an equivalent molar ratio of BF10 or its subcomponents, IL-10-Fc and BF10 both significantly suppressed tumor growth, and BF treatment performed best among all groups. In contrast, α-mCSF1R barely repressed tumors (Figure 3A). A trend of better survival was noted in the BF10 group compared with IL-10-Fc, α-mCSF-1R, or control IgG (Figure 3B). BF10 did not induce splenomegaly (Figure 3C) and caused no deleterious effect in liver compared to the elevated liver enzymes in mice receiving α-mCSF1R treatment (Figure 3D).

We next investigated the influence of different treatments on immune cells in the HNSCC model. Increased $CD8^+$ T cells and reduced $CD11b^+Ly6C^+$ monocyte numbers were noted in the peripheral blood of BF10-treated mice compared with control (Figure S4E). A significant enrichment in the infiltration of $CD8^+$ T cells was shown in the tumors after BF10 treatment (Figure S4F), especially in the central part of tumors (Figures 3E–3H and S4G). We further examined the tumor samples from four treatment groups (control, IL-10-Fc, α-mCSF-1R, BF10). BF10 treatment recruited more $CD8^+$ T cells to the central tumor part and reduced $F4/80^+$ TAM infiltration. α-mCSF-1R treatment reduced TAMs without significantly affecting intratumoral $CD8^+$ T cells. IL-10-Fc marginally increased intratumoral $CD8^+$ T cells without affecting TAMs (Figure S5A). Furthermore, we conducted an investigation into the influence of BF10 on various mouse models, including orthotopic and subcutaneous injection models, as the immune microenvironments in these models may differ. Orthotopic tumors exhibited a greater infiltration of $CD8^+$ T cells and macrophages compared with the control group. Notably, administration of BF10 significantly amplified the influx of $CD8^+$ T cells in tumors from both models, and this effect was more pronounced in the orthotopic model (Figure S5B).

We further examined whether both subcomponents of BF10 (IL-10 and α-mCSF1R) contributed to the antitumor efficacy through modulating their corresponding immune cells, i.e., $CD8^+$ T cells by IL-10 and TAMs by α-mCSF1R. We first depleted $CD8^+$ T cells in BF10-treated tumor-bearing mice (Figure 3I). Successful depletion of $CD8^+$ T cells was confirmed by analyzing the post-treatment blood and tumor samples (Figures S6A and S6B). Depletion of $CD8^+$ cells abrogated the antitumor effect of BF10 (Figures 3J, 3K, S6C, and S6D). We next depleted TAMs to confirm the antitumor role of the α-mCSF1R subcomponent in BF10 (Figure 3L). IL-10-Fc or BF10 monotherapy effectively suppressed tumor growth and possessed survival benefits as expected. Treatment with the macrophage-depleting agent clodronate-liposome suppressed tumor growth and improved survival marginally. Cotreatment with clodronate-liposome and IL-10-Fc showed a similar result to IL-10-Fc monotherapy. Importantly, cotreatment with clodronate-liposome and BF10 significantly attenuated the antitumor activity of BF10 (Figures 3M and 3N). Taken together, the above results indicate that BF10 provides potent antitumor effect and survival benefit and that both subcomponents of BF10 (IL-10 and α-mCSF1R) contributed to the antitumor efficacy.

BF10 modulates tumor-infiltrated immune cells and elicits immune activation

We next examined the impact of BF10 on the tumor-infiltrated immune cells in the murine orthotopic HNSCC model (Figure 4A). BF10 showed the most prominent increase in the infiltration of $CD8^+$ T cells. Reduction of TAMs was shown both in the αCSF-1R- and BF10-treated groups. A slightly increased trend in $CD4^+$ T cell was noted in the BF10-treated group compared with the control group (Figures 4B–4D). We further investigated the composition of infiltrated $CD8^+$ T cells (Figures 4E and 4F; the gating strategy of flow cytometry is in Figure S7). Both IL-10-Fc and BF10 treatments significantly increased the $GZMB^+$ cytotoxic population of $CD44^+CD8^+$ T cells within the TME (Figure 4E). In addition, different subsets of exhausted $CD8^+$ T cells responding to IL-10-Fc and BF10 was noted. BF10 treatment expanded the terminally exhausted $CD8^+$ T cells (Term Tex) population with a relatively mild increase in progenitor exhausted $CD8^+$ T cells (Prog Tex) (Figure 4F). In contrast, IL-10-Fc increased the Prog Tex population without significantly influencing the Term Tex population. We next applied the multicolor immunofluorescent (mIF) staining to examine the influence of BF10 treatment on infiltrated immune cells. Consistently, BF10 performed best at increasing the infiltrated $CD8^+$ T cells compared with its subcomponents (Figures 4G and 4H), and BF10 increased $GZMB^+$ cells as expected (Figure S8A). We examined the tdLNs and spleen from BF10-treated

Figure 4. BF10 repopulates infiltrated immune cells in tumor tissues and LNs

(A) Experimental scheme. The HNSC/Q1-2 tumor cells (5×10^5 cells) were orthotopically inoculated into mice, followed by administrations of the indicated components (Ctrl-IgG, IL-10-Fc, αCSF1R, and BF10) with a 3 day interval for 3 doses. Tumor-infiltrated $CD45^+$ immune cells were assessed by flow cytometry.
(B–D) Population of $CD8^+$ T cells (B), TAMs (C), and $CD4^+$ T cells (D) in tumors from the indicated groups.
(E) Representative plots (left) and population (right) of the granzyme B (GZMB)-producing tumor-infiltrating T cells from the indicated tumors.
(F) Representative population of the $TCF1^+$ $Tim3^-$ T cells (Prog Tex) and the $TCF1^-$ $Tim3^+$ T cells (Term Tex) among total tumor-infiltrating $CD44^+$ $PD\text{-}1^+$ $CD8^+$ T cells from the indicated mice.
(G–H) Representative images of $CD8^+$ T cell numbers from the indicated groups.
(G) Multiplexed immunofluorescence (mIF) staining performed with Opal 7-Color IHC kit (PerkinElmer) for CD4 (green), CD8 (sky blue), GZMB (red), and nuclei (hyacinth). Representative composite images obtained and quantified by the Vectra Polaris Imaging System and Inform software. Scale bar, 100 μm.
(H) Quantitative result of $CD8^+$ T cells.
(I–K) Representative image of Ki67 expression in tumor-draining LNs (tdLNs).
(I) The isolated tdLN examined by immunohistochemistry (IHC). The brown color indicates $Ki67^+$ cells. Scale bar, 300 μm.
(J) Representative mIF images of tdLN. The isolated tdLNs were stained with DAPI (blue), CD8 (green), PD-1 (yellow), FoxP3, CD19 (pink), and Ki67 (red). Whole-tissue composite images were captured and analyzed with the Vectra Polaris Imaging System and Inform software. Scale bar, 400 μm.
(K) Quantification of the cell number of $CD8^+PD1^+$, $CD8^+PD1^+Ki67^+$, and $CD19^+$ cells in the BF10-treated versus control group. The data were presented as mean ± SD, and the statistics were calculated using unpaired Student's t test. (two group comparison) and one-way ANOVA (more than 3 groups) with an appropriate test. $^{**}p < 0.01$.

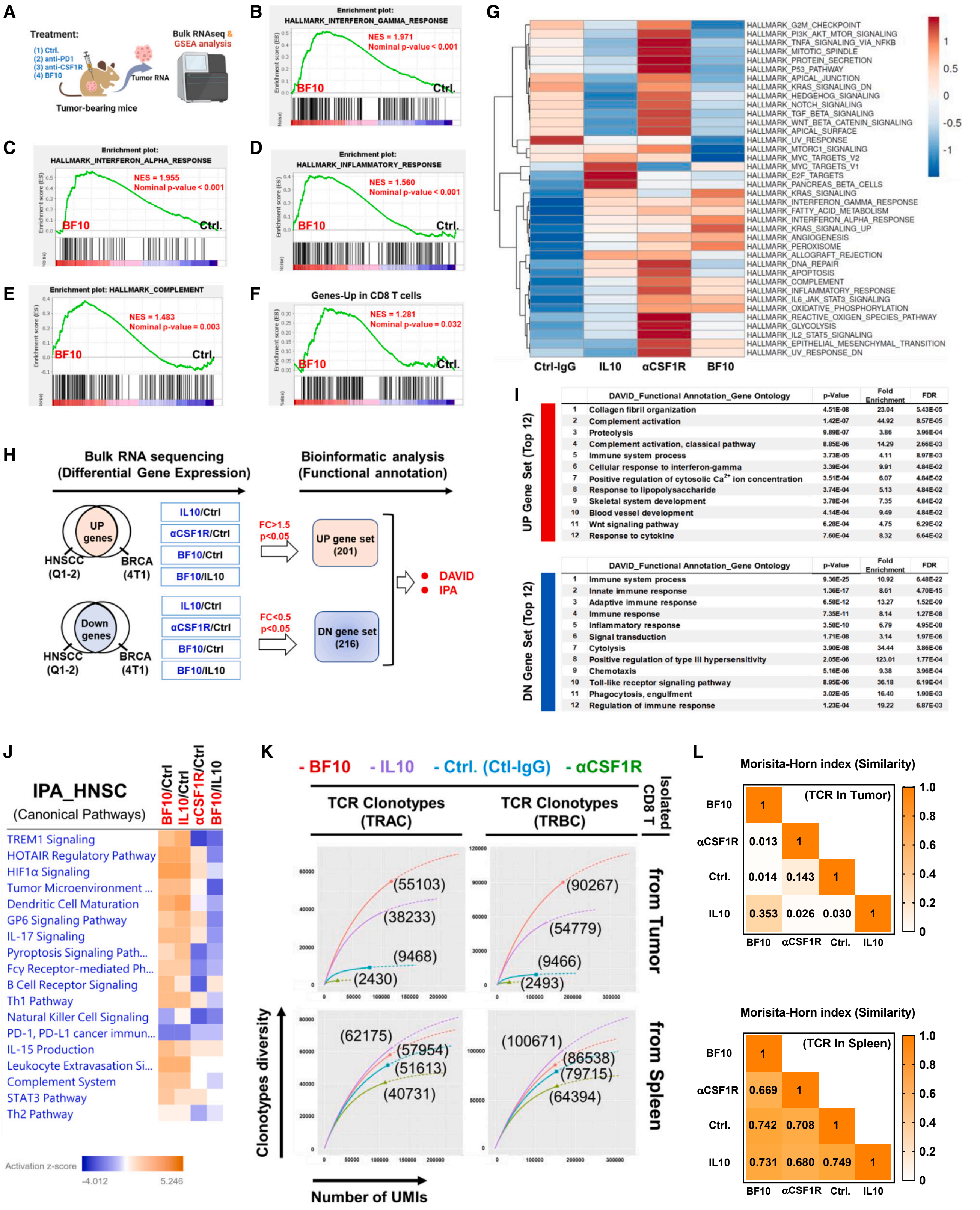

(legend on next page)

mice. Treatment with BF10 significantly boosted the proliferation of immune cells in the germinal centers of spleens and tdLNs (Figures 4I, S8D, and S8E). Further examination of tdLNs showed that BF10 treatment increased the numbers as well as the density of CD8$^+$PD-1$^+$, CD8$^+$PD-1$^+$Ki67$^+$, and CD19$^+$ cells (Figures 4J, 4K, S8C, S8D, and S8F). The above results indicate that BF10 enriches the number and activity of tumor-infiltrated T cells as well as induces immune activation.

BF10 activates multiple immune-related signaling pathways and expands the clonal diversity of tumor-infiltrated T cells

We compared the impact of BF10 treatment on the transcriptomic changes of tumors to the subcomponent treatment (IL-10-Fc and α-mCSF1R) (Figure 5A). A gene set enrichment analysis (GSEA) showed that BF10 treatment was associated with the signature of IFN-γ response (Figure 5B), IFN-α response (Figure 5C), inflammatory response (Figure 5D), complement (Figure 5E), and genes upregulated in CD8$^+$ T cells (GEO: GSE41867) (Figure 5F). Moreover, the BF10 treatment group revealed the most enriched expanded immune signature (Table S4)[42] compared with IL-10-Fc, αCSF1R, and control (Figure S9A). Single-sample GSEA (ssGSEA) revealed a gene expression profile of BF10-treated tumors compared with the treatment of subcomponents or control (Figure 5G).

Next, we investigated potential signaling pathways influenced by BF10. We enrolled two syngeneic murine tumor models (4T1/Balb-c BRCA model and MTCQ1-2/C57BL/6 HNSCC model) to reduce the influence of tumor-type-specific effect and treated the mice with control IgG, IL-10-Fc, αCSF-1R, or BF10. Tumors were obtained for RNA sequencing (RNA-seq) and for further analysis of the consistently upregulated/downregulated genes in both models (Figure 5H; Tables S5 and S6 for HNSCC and S7 and S8 for BRCA). A significant proportion of the top 12 upregulated/downregulated gene-related pathways were associated with immune responses (Figure 5I), which validated the immunomodulatory effects of these agents. We further focused on examining the genes/pathways influenced by different treatments in HNSCC through multiple comparisons. Compared with the control group, both BF10 and IL-10-Fc activated the expression of immune-related genes and pathways, effects that were mostly not observed in the αCSF-1R-treated group. For example, *Wnt7a*, *Mmp9*, *Stfa2*, *C1s2*, *Elane*, *Apo11a*, and *H2-Dmb2* were upregulated in IL-10-Fc- and BF10-treated samples but not in αCSF1R-treated tumors. In contrast, *Clec4a*, *Clec4b1*, *Dpep2*, *Slamf9*, and *Lilra5α* were downregulated in the αCSF1R group but not in the IL-10-Fc and BF10 groups (Figures S9B–S9D). Many pathways, such as TREM1 signaling, the TME pathway, DC maturation, Fcγ receptor-mediated phagocytosis, leukocyte extravasation signaling, and the complement system, were activated both in the IL-10-Fc- and BF10-treated samples but not in the αCSF1R-treated tumors (Figure 5J). Two pathways were specifically activated in the BF10-treated group compared with the IL-10-Fc group: B cell receptor signaling and IL-15 production (Figure 5J).

Recognition of tumor-associated antigen by T cell receptor (TCR) is a major determining factor for effective antitumor immune response. High diversity of TCR repertoire is associated with a better prognosis and with better responses to immune checkpoint blockade therapies.[43,44] Here, we analyzed the TCR repertoire of CD8$^+$ cells harvested from tumors, tdLNs, and spleens of mice receiving treatment of BF10 or subcomponents versus control (Figure S9E). Tumor-specific TCRα and TCRβ sequencing demonstrated that the CD8$^+$ cells from tumors or tdLNs of BF10-treated mice harbored the highest clonotype diversity compared with the other groups, whereas IL-10-Fc treatment increased the diversity of the TCR repertoire of CD8$^+$ cells from spleen (Figures 5K and S9F). The Morisita-Horn calculation index and the TCR clonotype report supported the fact that BF10 treatment induces the most clonotype diversity compared with the other treatments (Figures 5L and S9G; Table S9). In summary, the above results indicate that BF10 outperforms subcomponent treatments in the active immune signature of tumor samples and that BF10 increases TCR clonotype diversity in CD8$^+$ cells from tumors and tdLNs to the greatest extent compared with subcomponents or control.

BF10 repopulates tumor-infiltrated T cells by increasing Term Tex cells and reducing regulatory T cells

To further understand the impact of BF10 on tumor-infiltrated immune cells, single-cell RNA-seq (scRNA-seq) was performed in murine orthotopic HNSCC tumors treated with IL-10-Fc, αCSF1R, BF10, or a control IgG. CD45$^+$ cells were isolated from the tumor samples and analyzed using scRNA-seq, and CD45$^-$ cells were reserved for bulk RNA-seq analysis. The

Figure 5. Transcriptomic and TCR repertoire analysis of the samples from mice treated with BF10 versus its subcomponents

(A) An illustration of mice receiving the indicated treatment followed by tumor isolation for bulk RNA-seq and gene set enrichment analysis (GSEA).

(B–F) Enrichment plots of genes sets in BF10 versus control in mouse tumor samples. Significant pathways were identified by GSEA gene set of IFN-γ response (B), IFN-α response (C), inflammatory response (D), complement (E), and genes upregulated in CD8 T cells (GEO: GSE41867) (F).

(G) The heatmap of GSEA enriched pathways in treatment groups. The normalized enrichment score (NES) and p value are shown.

(H) Schema of bioinformatic strategy to study the effect of BF10 within tumor microenvironment. Tumors derived from two syngeneic tumor models (HNSC/Q1-2 and BRCA/4T1) treated with BF10, IL-10-Fc, αCSF-1R, or control were collected for bulk RNA-seq to investigate the differentially expressed genes (DEGs; >1.5-fold and $p < 0.05$ or <0.5-fold and $p < 0.05$). DEGs were analyzed using 2 modules: (1) Bioinformatics Database for Annotation, Visualization, and Integrated Discovery (DAVID; https://david.ncifcrf.gov/) and (2) Ingenuity Pathway Analysis (IPA).

(I) DAVID functional Gene Ontology analysis.

(J) IPA.

(K) Immune repertoire TCR sequencing of CD8$^+$ T cells. Tumor-bearing mice received treatments of Ctrl-IgG, IL-10, anti-CSF1R, or BF10 for 3 doses, followed by RNA extraction of isolated CD8$^+$ T cells for TCR immune repertoire analysis. The data are shown as clonotype diversity and distribution of both TRAC and TRBC CDR3 sequencing from tumor and spleen. The number of the bracket indicates observed diversity in the enrichment metrics of QIAseq-RNA Immune Repertoire Application.

(L) Examination of the TCR immune repertoire using Morisita-Horn index.

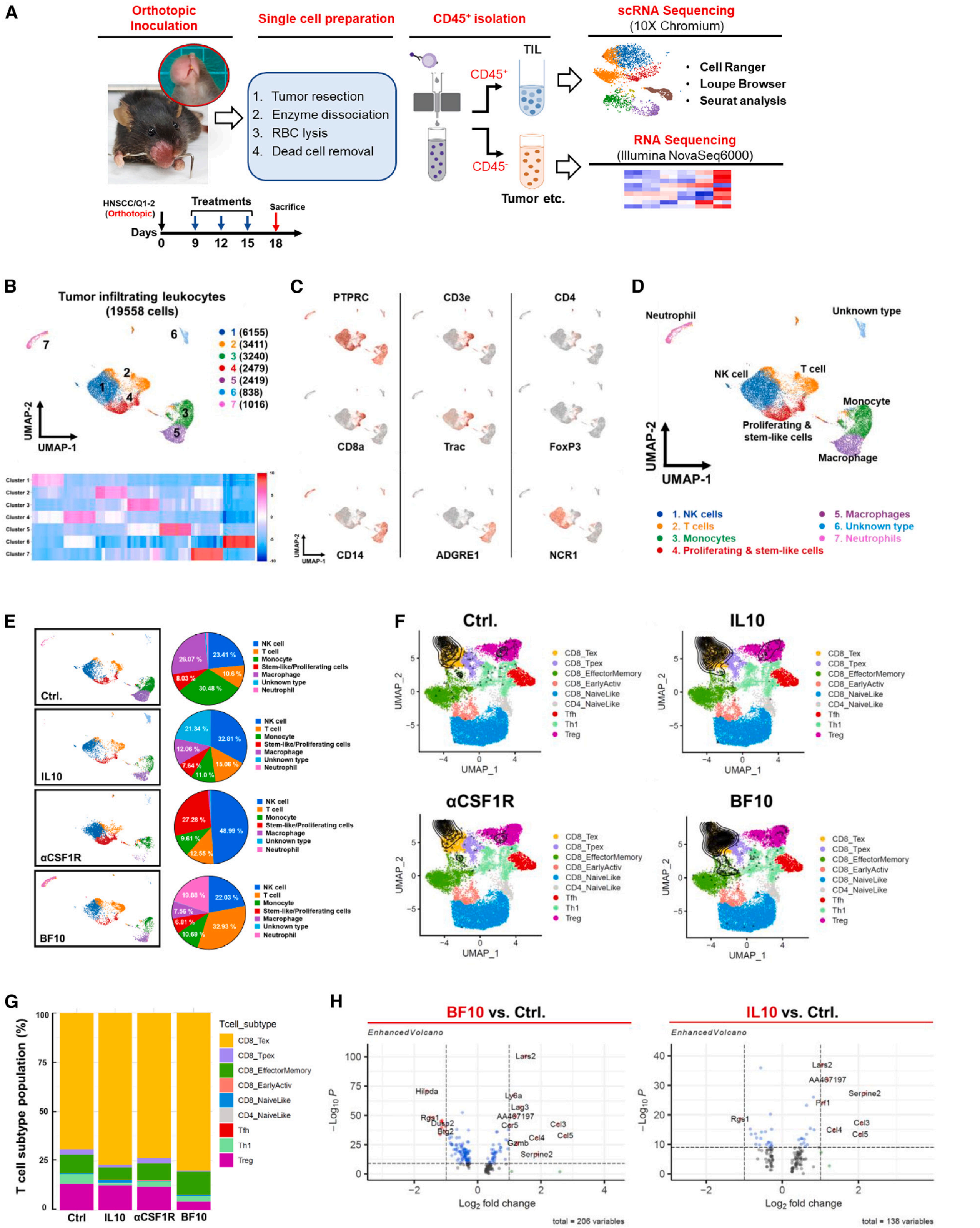

(legend on next page)

flowchart is illustrated in Figure 6A. Bulk RNA-seq of the $CD45^{-}$ cells showed that BF10 was associated with the reactive oxygen species (ROS) pathway, IFN-γ response, and inflammatory response as compared with the control treatment (Figure S10A). Compared with the IL-10-Fc treatment group, BF10 treatment was also more closely associated with the signatures of ROS, IFN-γ response, and inflammatory response (Figure S10B). For scRNA-seq of $CD45^{+}$ cells, sorted tumor-infiltrated leukocytes (19,558 cells) were clustered into 7 groups (Figure 6B). The expression of the immune markers in different clusters is shown in Figures 6C and 7, and the populations of immune cells were defined as shown in Figure 6D. Among the three treatment groups, T cells were mostly enriched in the BF10 group. TAMs were depleted in the αCSF-1R group and reduced in the BF10 group, as expected. Interestingly, monocytes were significantly reduced in all three treatment groups, and NK cells were most prominently enriched in the αCSF-1R group. An unexpected increase in neutrophils was noted in the BF10 group (Figure 6E). We further dissected the T cell subpopulations. By observing the relative percentages of subgroups, BF10 reduced the regulatory T cell (Treg) population and increased the Term Tex population, whereas the IL-10-Fc group also increased the Term Tex population, albeit to a lesser degree, without significantly influencing Tregs. αCSF1R treatment did not influence the T cell subpopulations (Figures 6F and 6G). BF10 upregulated the genes related to T cell exhaustion more significantly than IL-10-Fc (Figure 6H). To address the role of neutrophils in BF10-mediated antitumor effect, a neutrophil-depletion experiment was performed. Neutrophil depletion causes a minor effect on tumor growth and survival of the mice (Figures S10C–S10F). In summary, the results indicate that BF10 shapes T cell subpopulations by increasing $CD8^{+}$ Term Tex and reducing the percentage of Tregs, which may contribute to its antitumor effect.

BF10 elicits durable protective effect and potentiates anti-PD1 efficacy

We next investigated the effect of BF10 under tumor rechallenge. We treated HNSCC tumor-bearing mice with BF10 until total regression of the tumors was reached. The tumor-free (TF) mice were selected for subsequent tumor rechallenge experiments (Figure 7A). A significant and durable protective effect was demonstrated in the BF10-treated mice compared with the unexposed mice (Figures 7B and 7C). An *ex vivo* examination of the parental tumor cell line cocultured with CD8 T^{+} isolated cells from BF10-treated TF mice showed that $CD8^{+}$ T cells from BF10-treated mice were able to repress tumor cell growth (Figures S11A and S11B).

Next, we examined whether BF10 potentiates the antitumor activity of anti-PD1 (αPD1) because BF10 increased the diversity of the TCR repertoire of tumor-infiltrating lymphocytes (TILs) (Figures 5K–5L), which is associated with the response to immune checkpoint blockade.[43,44] Furthermore, BF10 increased the $CD8^{+}$ Term Tex population (Figures 4F, 6F, and 6G). Mice with HNSCC were subjected to the treatment of BF10, αPD1, or both in combination versus control (Figure 7D). BF10 revealed a significant antitumor effect consistent with previous data, whereas αPD1 treatment showed a moderate effect in murine HNSCC. The combination of BF10 with αPD1 showed nearly complete suppression of the tumors (Figures 7E, S11C, and S11D). The tumor volume changes and the endpoint tumor volume also supported a better antitumor efficacy in the combination treatment group compared with the single agents (Figures 7F and 7G). Treatment with BF10, αPD1, or the combination did not alter the body weight of the mice (Figure 7H). We collected the sera of the mice for cytokinome analysis (Figure S11E). Combination treatment harbored a higher effect in producing inflammatory cytokines (IFN-γ, IL-1β, IL-2, IL-6, IL-10, IL-12p40, and TNF-α) and certain chemokines (e.g., CCL-11, eotaxin-2, CCL-1, CCL-2, and CXCL-9) than single treatments (Figures 7I and 7J). The angiogenic factors were not significantly and consistently influenced by the combination treatment (Figure S11F). In summary, the combination of BF10 and αPD1 provided better efficacy against murine HNSCC. An increased induction of certain proinflammatory cytokines and chemokines is shown in the combination treatment group, which might contribute to the immune cell recruitment and antitumor activities induced by BF10 and αPD1.

DISCUSSION

In this study, we generated a proof-of-concept bifunctional antibody, BF10, by fusing an anti-CSF-1R antibody with IL-10. The goal of this design is to enhance the delivery of IL-10 to the TME and concurrently shape the immune environment. We selected CSF-1R as the target of the "basal" antibody based on the following. First, we validated the clinical relevance of *IL-10/IL-10R* and *CSF1R* in tumor samples. Second, there was a high proportion of TAMs in tumor-infiltrated immune cells of HNSCC. Third,

Figure 6. scRNA-seq analysis of syngeneic HNSCC treated with BF10 or its subcomponents

(A) Experimental scheme for scRNA-seq. TILs were isolated. Single-cell libraries were prepared using a Chromium NextGEM Single Cell 3′ Reagent Kit (v.3.1 kit) and sequenced with Illumina NovaSeq 6000. Data were processed and analyzed with Cell Ranger pipeline (v.5.0.1, 10× Genomics) and Loupe browser (v.5.0). A detailed analysis of T cell subtype was assessed with ProjecTILs (v.1.0). $CD45^{-}$ cells were harvested for bulk RNA-seq.

(B) Top, uniform manifold approximation and projection (UMAP) plot of the total cells colored by the 7 major cell lineages. The cell counts of each cluster are indicated in brackets. Bottom, a heatmap to represent the top 10 upregulated genes of each cluster.

(C) UMAP plots of markers for different types of immune cells.

(D) UMAP plot of color-coded immune cell types as indicated (reference: CellKb, https://www.cellkb.com/immune.).

(E) Left, UMAP plots of cell-type distributions in different treatment groups. Right, pie charts showing the immune cell compositions of different treatment groups.

(F) Projection of T cells into a TIL reference atlas using ProjecTILs in tumors of different treatment groups. Colored cells represent the reference states defined by previous literature[45]; black cells represent projected cells from the treatment groups, and black contour lines represent the density of projected cells over the atlas.

(G) T cell subtype composition and proportion of the four treatment conditions with subtypes defined by ProjecTILs classifier.

(H) DEGs in exhausted T cells (Texs) between pairs of treatments.

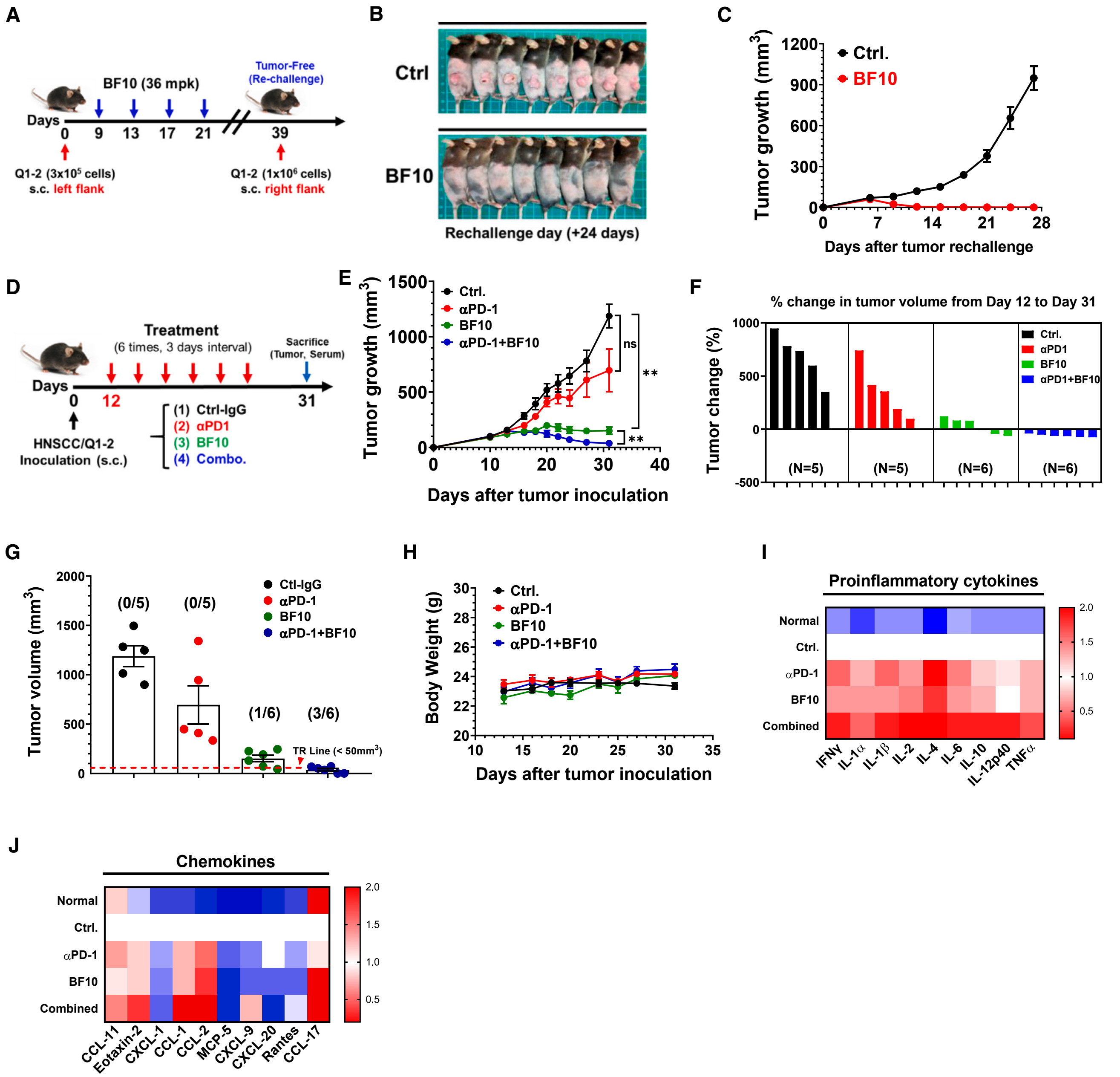

Figure 7. BF10 provides durable antitumor effect and potentiates anti-PD1 therapy

(A) Schema of the tumor rechallenge experiments. HNSCC/Q1-2 tumor cells (3×10^5 cells) were subcutaneously injected into left flank of mice followed by BF10 treatment until tumor regression. Tumor-free mice were selected and rechallenged with a secondary implantation of the tumor cells (1×10^6 cells) on the right flank. Tumor growth was recorded for 1 month after rechallenge.

(B) Representative photos of the mice that received tumor rechallenge.

(C) Tumor volume curve of the BF10-treated, tumor-free mice or control mice. The data are shown as means ± SEM (N = 10–16 per group).

(D–J) Tumor-bearing mice (Q1-2, subcutaneous) were treated with isotype control or anti-PD1 (200 μg) and/or BF10 (36 mg/kg) as indicated.

(D) Experimental scheme. Treatment started when tumor size reached 100 mm^3. The mice were sacrificed on day 31.

(E)Tumor growth curve. The data were presented as mean ± SD, and the statistics were calculated by one-way ANOVA with multiple comparisons test. **$p < 0.01$.

(F) Waterfall plot showed tumor volume percentage change of individual mice.

(G) The ratio of tumor-free mice over total mice in each group (N = 5–6 per group). Tumor-free volume was defined as <50 mm^3.

(H) The measurements of body weights in mice.

(I and J) The serum (N = 4 per group) from mice with indicated treatments was analyzed using the Mouse Cytokine Antibody Array (RayBiotech, QAM-CAA-4000 for 200 cytokines). The signals of dots are normalized to internal positive and negative controls on the same array. The calculated data values are shown as heatmaps of proinflammatory cytokines (I) and chemokines (J).

the importance of the physical interplay between APCs (especially TAMs) and T cells (especially exhausted T cells) within the TME has been gradually uncovered.[31,46] Fourth, the efficient delivery of macrophage-targeting agents to tumor sites by either peripheral macrophages or TAMs was demonstrated. The results of this study highlight the potential development of antibody-fused IL-10-Fc in the future. The selection of basal antibodies can be extended to target the proteins enriched in the TME with immunomodulatory functions.

In our study, significant antitumor activity was demonstrated in both BF10 and IL-10-Fc, and BF10 outperformed its subcomponents IL-10 and α-mCSF1R. The potential explanations include the following. First, the antitumor activity of BF10 was abolished by depletion of either $CD8^+$ T cells or TAMs, indicating that both subcomponents contribute to its therapeutic efficacy. Second, BF10 increased the greatest clonotype diversity of TCRs of $CD8^+$ T cells in tumors or tdLNs compared with its subcomponents. Third, BF10 has the best ability to reprogram the TME to an immune-active state, as proven by transcriptomic experiments. Interestingly, BF10 had a better ability to enrich Term Tex and reduce Tregs compared with IL-10-Fc. The BF10 biodistribution experiment indicates that BF10 accumulated not only in the tumor but also in the tdLN and spleen, indicating the potential extra-tumoral immunomodulatory effects of BF10.

Here, we selected HNSCC as the major target disease because of the abundance of TAMs in HNSCC in previous studies[47,48] and our analyses, and the accumulation of TAMs in the TME negatively correlated with the clinical outcomes of patients with HNSCC.[47] In recent years, the paradigm shift of the treatment in recurrent/metastatic (R/M) HNSCC has been due to the great success of immune checkpoint inhibitors (ICIs).[49–51] However, the relatively low response rate of ICI monotherapy reduces enthusiasm and indicates persistent unmet medical needs in R/M HNSCC. Recent studies have suggested that ICIs alone may not be sufficient because of the dominant role of the TME in governing tumor immune surveillance and cancer immune evasion.[52–54] Therefore, simultaneous activation of tumor-specific T cells and shaping of the TME serve as an opportunity to resolve the shortcomings of current therapies.

In summary, we present the antitumor efficacy of the bifunctional fusion protein BF10 in multiple murine models, especially HNSCC. BF10 enhances $CD8^+$ T cell recruitment to the central tumor region, induces proliferation and metabolic reprogramming of $CD8^+$ T cells, and reduces Tregs and TAMs. The combination of BF10 and αPD-1 further enhances antitumor activity and boosts proinflammatory cytokine production. Our results provide a promising drug design strategy for the future development of immuno-oncology agents.

Limitations of the study

A significant limitation of the current study is the lack of evaluation regarding the impact of BF10 on tumor-specific T cells in HNSCC. This is primarily due to the unavailability of a suitable model, such as Yumm1.7 for melanoma, to investigate tumor-specific T cells in HNSCC. We did not employ the Yumm1.7 model in this study due to our focus on HNSCC. Nonetheless, it is imperative to establish a tumor-specific T cell model to explore the antitumor response for future advancements in anti-HNSCC immunotherapy.

STAR★METHODS

Detailed methods are provided in the online version of this paper and include the following:

- KEY RESOURCES TABLE
- RESOURCE AVAILABILITY
  - Lead contact
  - Materials availability
  - Data and code availability
- EXPERIMENTAL MODEL AND SUBJECT DETAILS
  - Human HNSCC samples
  - Mice
  - Cell lines
- METHOD DETAILS
  - Bioinformatics analysis of The Cancer Genome Atlas (TCGA) and VGH_HNSCC patients
  - Digital spatial profiling (DSP) of RNA analysis in HNSCC samples
  - Tumor model and treatment
  - MuScreen examination of BF10 antitumor activity in syngeneic tumor models
  - Generation of bifunctional anti-CSF-1R/IL-10 fusion proteins
  - ELISA of BF10 binding activity and neutralization
  - Inhibition of CSF-1-dependent cell survival of macrophages by anti-CSF1R antibodies
  - IL10R-STAT3 reporter assay
  - $CD8^+$ T cell proliferation assay
  - Seahorse metabolomics analysis
  - Serum pharmacokinetics of BF10
  - Preparation of ^{111}In-labeled BF10 and the animal SPECT/CT imaging of ^{111}In-labeled BF10
  - Biodistribution studies of ^{111}In-labeled BF10 and the blood clearance kinetic of ^{111}In-labeled BF10
  - Activation of $CD8^+$ T cells by IL-10
  - Antibody labeling, biodistribution and *in vivo* imaging
  - Immunohistochemistry (IHC)
  - *In vivo* depletion of $CD8^+$ T cells or macrophages
  - Flow cytometry assays
  - Multiplex immunofluorescence staining of mouse samples
  - Bulk RNA sequencing and biologic inference
  - Generation of mouse TCR immune repertoire sequencing data
  - Single-cell RNA sequencing
  - Neutrophil depletion
  - Combination therapy of BF10 with anti-PD-1 blockade
  - Cytokine array assay
- QUANTIFICATION AND STATISTICAL ANALYSIS

SUPPLEMENTAL INFORMATION

Supplemental information can be found online at https://doi.org/10.1016/j.xcrm.2023.101154.

ACKNOWLEDGMENTS

We acknowledge the technical services of Wan-Chun Chang, Bingxiu Guo, Chih-Sin Hsu, Chien-Yi Tung, and Kate Hua provided by the Genomics Center for Clinical and Biotechnological Applications of the Cancer Progression Research Center (National Yang Ming Chiao Tung University), The National Core Facility for Biopharmaceuticals of National Science and Technology Council. We thank Dr. Kuo-Wei Chang (National Yang Ming Chiao Tung University) for providing the mouse head and neck cancer cell line MTC-Q1 and Dr. Szu-Ting Chen (National Yang Ming Chiao Tung University) for the experimental comments. All human tumor samples for this study were obtained from the Biobank of Taipei Veterans General Hospital. We thank Dr. Chih-Hung Chung, Mr. Yu-Hsien Chen, and Ms. Jia-Jia Yang (National Yang Ming Chiao Tung University) for their technical assistance. This work was supported by grants from the National Council for Science and Technology (NSTC 111-2321-B-A49-007; MOST 110-2740-B-A49A-501; MOST 109-2740-B-010-002; MOST 108-2319-B-010-001; MOST-108-2314-B-010-020-MY3; MOST 110-2320-B-A49A-542; MOST 111-2320-B-A49-007; and MOST 111-2314-B-A49-030-MY3 to M.-H.Y.); the National Health Research Institutes (NHRI-EX112-11215BI to M.-H.Y.); the Taipei Veterans General Hospital (V112C-130, V112E-002-2, V111C-013, and V111E-001-1 to M.-H.Y.); the Taipei Veterans General Hospital-National Taiwan University Hospital Joint Research Project (VN111-13 to M.-H.Y.); the Research Project for Industry-Academia Collaboration of National Yang Ming Chiao Tung University (YM108C084, YM108C084-1, and YC-Y21C041 to M.-H.Y.); the Cancer Progression Research Center (111W31101) and the Cancer and Immunology Research Center (112W31101) of Featured Areas Research Center Program within the framework of the Higher Education Sprout Project by the Ministry of Education (to M.-H.Y.); the Department of Health, Taipei City Government (no. 11201-62-042 to M.-H.Y.); Elixiron Immunotherapeutics (to P.-C.H.); a European Research Council Staring Grant (802773-MitoGuide to P.-C.H.); SNSF project grants (31003A_182470 to P.-C.H.); the Cancer Research Institute Lloyd J. Old STAR award (to P.-C.H.); and Ludwig Cancer Research (to P.-C.H.).

AUTHOR CONTRIBUTIONS

Conceptualization, Y.-W.C., C.-Y.W., C.-H.T., P.A.K., P.-C.H., H.-K.C., and M.-H.Y.; data curation, investigation, and formal analysis, Y.-W.C., H.-W.H., J.-P.C., H.-H.H., S.-F.T., S.J.C., and M.-T.S.; methodology, resources, software, and analysis, H.-W.H., J.-P.C., M.A., and G.D.C.; writing – original draft & editing, Y.-W.C., H.-W.H., P.A.K., and M.-H.Y.; supervision, writing – review & editing, and funding acquisition, P.-C.H., H.-K.C., and M.-H.Y.

DECLARATION OF INTERESTS

P.-C.H. is a scientific advisor for Elixiron Immunotherapeutics, Acepodia, and Novartis and is a cofounder of Pilatus Biosciences. P.-C.H. received research support from Elixiron Immunotherapeutics. P.-C.H. also received research support from Roche.

INCLUSION AND DIVERSITY

We support inclusive, diverse, and equitable conduct of research.

Received: November 3, 2022
Revised: June 4, 2023
Accepted: July 18, 2023
Published: August 15, 2023

REFERENCES

1. DeNardo, D.G., and Ruffell, B. (2019). Macrophages as regulators of tumour immunity and immunotherapy. Nat. Rev. Immunol. *19*, 369–382. https://doi.org/10.1038/s41577-019-0127-6.
2. Myers, K.V., Amend, S.R., and Pienta, K.J. (2019). Targeting Tyro3, Axl and MerTK (TAM receptors): implications for macrophages in the tumor microenvironment. Mol. Cancer *18*, 94. https://doi.org/10.1186/s12943-019-1022-2.
3. Lee, C.C., Lin, J.C., Hwang, W.L., Kuo, Y.J., Chen, H.K., Tai, S.K., Lin, C.C., and Yang, M.H. (2018). Macrophage-secreted interleukin-35 regulates cancer cell plasticity to facilitate metastatic colonization. Nat. Commun. *9*, 3763. https://doi.org/10.1038/s41467-018-06268-0.
4. Wei, C., Yang, C., Wang, S., Shi, D., Zhang, C., Lin, X., Liu, Q., Dou, R., and Xiong, B. (2019). Crosstalk between cancer cells and tumor associated macrophages is required for mesenchymal circulating tumor cell-mediated colorectal cancer metastasis. Mol. Cancer *18*, 64. https://doi.org/10.1186/s12943-019-0976-4.
5. Liang, Z.W., Ge, X.X., Xu, M.D., Qin, H., Wu, M.Y., Shen, M., Zhang, Y., Liu, X.M., Chen, K., Li, W., et al. (2021). Tumor-associated macrophages promote the metastasis and growth of non-small-cell lung cancer cells through NF-kappaB/PP2Ac-positive feedback loop. Cancer Sci. *112*, 2140–2157. https://doi.org/10.1111/cas.14863.
6. Feng, Q., Chang, W., Mao, Y., He, G., Zheng, P., Tang, W., Wei, Y., Ren, L., Zhu, D., Ji, M., et al. (2019). Tumor-associated macrophages as prognostic and predictive biomarkers for postoperative adjuvant chemotherapy in patients with stage II colon cancer. Clin. Cancer Res. *25*, 3896–3907. https://doi.org/10.1158/1078-0432.CCR-18-2076.
7. Yuan, X., Zhang, J., Li, D., Mao, Y., Mo, F., Du, W., and Ma, X. (2017). Prognostic significance of tumor-associated macrophages in ovarian cancer: a meta-analysis. Gynecol. Oncol. *147*, 181–187. https://doi.org/10.1016/j.ygyno.2017.07.007.
8. Forssell, J., Oberg, A., Henriksson, M.L., Stenling, R., Jung, A., and Palmqvist, R. (2007). High macrophage infiltration along the tumor front correlates with improved survival in colon cancer. Clin. Cancer Res. *13*, 1472–1479. https://doi.org/10.1158/1078-0432.CCR-06-2073.
9. Chen, Y., Jin, H., Song, Y., Huang, T., Cao, J., Tang, Q., and Zou, Z. (2021). Targeting tumor-associated macrophages: a potential treatment for solid tumors. J. Cell. Physiol. *236*, 3445–3465. https://doi.org/10.1002/jcp.30139.
10. Li, M., Li, M., Yang, Y., Liu, Y., Xie, H., Yu, Q., Tian, L., Tang, X., Ren, K., Li, J., et al. (2020). Remodeling tumor immune microenvironment via targeted blockade of PI3K-gamma and CSF-1/CSF-1R pathways in tumor associated macrophages for pancreatic cancer therapy. J. Contr. Release *321*, 23–35. https://doi.org/10.1016/j.jconrel.2020.02.011.
11. Akkari, L., Bowman, R.L., Tessier, J., Klemm, F., Handgraaf, S.M., de Groot, M., Quail, D.F., Tillard, L., Gadiot, J., Huse, J.T., et al. (2020). Dynamic changes in glioma macrophage populations after radiotherapy reveal CSF-1R inhibition as a strategy to overcome resistance. Sci. Transl. Med. *12*, eaaw7843. https://doi.org/10.1126/scitranslmed.aaw7843.
12. Han, S., Wang, W., Wang, S., Yang, T., Zhang, G., Wang, D., Ju, R., Lu, Y., Wang, H., and Wang, L. (2021). Tumor microenvironment remodeling and tumor therapy based on M2-like tumor associated macrophage-targeting nano-complexes. Theranostics *11*, 2892–2916. https://doi.org/10.7150/thno.50928.
13. Denny, W.A., and Flanagan, J.U. (2021). Small-molecule CSF1R kinase inhibitors; review of patents 2015-present. Expert Opin. Ther. Pat. *31*, 107–117. https://doi.org/10.1080/13543776.2021.1839414.
14. Ordentlich, P. (2021). Clinical evaluation of colony-stimulating factor 1 receptor inhibitors. Semin. Immunol. *54*, 101514. https://doi.org/10.1016/j.smim.2021.101514.
15. Ries, C.H., Cannarile, M.A., Hoves, S., Benz, J., Wartha, K., Runza, V., Rey-Giraud, F., Pradel, L.P., Feuerhake, F., Klaman, I., et al. (2014). Targeting tumor-associated macrophages with anti-CSF-1R antibody reveals a strategy for cancer therapy. Cancer Cell *25*, 846–859. https://doi.org/10.1016/j.ccr.2014.05.016.
16. Falchook, G.S., Peeters, M., Rottey, S., Dirix, L.Y., Obermannova, R., Cohen, J.E., Perets, R., Frommer, R.S., Bauer, T.M., Wang, J.S., et al. (2021). A phase 1a/1b trial of CSF-1R inhibitor LY3022855 in combination with durvalumab or tremelimumab in patients with advanced solid tumors. Invest. N. Drugs *39*, 1284–1297. https://doi.org/10.1007/s10637-021-01088-4.
17. Razak, A.R., Cleary, J.M., Moreno, V., Boyer, M., Calvo Aller, E., Edenfield, W., Tie, J., Harvey, R.D., Rutten, A., Shah, M.A., et al. (2020). Safety and efficacy of AMG 820, an anti-colony-stimulating factor 1 receptor antibody, in

CellPress
OPEN ACCESS

combination with pembrolizumab in adults with advanced solid tumors. J. Immunother. Cancer *8*, e001006. https://doi.org/10.1136/jitc-2020-001006.

18. Gomez-Roca, C.A., Italiano, A., Le Tourneau, C., Cassier, P.A., Toulmonde, M., D'Angelo, S.P., Campone, M., Weber, K.L., Loirat, D., Cannarile, M.A., et al. (2019). Phase I study of emactuzumab single agent or in combination with paclitaxel in patients with advanced/metastatic solid tumors reveals depletion of immunosuppressive M2-like macrophages. Ann. Oncol. *30*, 1381–1392. https://doi.org/10.1093/annonc/mdz163.

19. Fiorentino, D.F., Zlotnik, A., Vieira, P., Mosmann, T.R., Howard, M., Moore, K.W., and O'Garra, A. (1991). IL-10 acts on the antigen-presenting cell to inhibit cytokine production by Th1 cells. J. Immunol. *146*, 3444–3451.

20. Saraiva, M., Vieira, P., and O'Garra, A. (2020). Biology and therapeutic potential of interleukin-10. J. Exp. Med. *217*, e20190418. https://doi.org/10.1084/jem.20190418.

21. Rallis, K.S., Corrigan, A.E., Dadah, H., George, A.M., Keshwara, S.M., Sideris, M., and Szabados, B. (2021). Cytokine-based cancer immunotherapy: challenges and opportunities for IL-10. Anticancer Res. *41*, 3247–3252. https://doi.org/10.21873/anticanres.15110.

22. Oft, M. (2019). Immune regulation and cytotoxic T cell activation of IL-10 agonists - Preclinical and clinical experience. Semin. Immunol. *44*, 101325. https://doi.org/10.1016/j.smim.2019.101325.

23. Naing, A., Infante, J.R., Papadopoulos, K.P., Chan, I.H., Shen, C., Ratti, N.P., Rojo, B., Autio, K.A., Wong, D.J., Patel, M.R., et al. (2018). PEGylated IL-10 (pegilodecakin) induces systemic immune activation, CD8(+) T cell invigoration and polyclonal T cell expansion in cancer patients. Cancer Cell *34*, 775–791.e3. https://doi.org/10.1016/j.ccell.2018.10.007.

24. Zheng, L.M., Ojcius, D.M., Garaud, F., Roth, C., Maxwell, E., Li, Z., Rong, H., Chen, J., Wang, X.Y., Catino, J.J., and King, I. (1996). Interleukin-10 inhibits tumor metastasis through an NK cell-dependent mechanism. J. Exp. Med. *184*, 579–584. https://doi.org/10.1084/jem.184.2.579.

25. Emmerich, J., Mumm, J.B., Chan, I.H., LaFace, D., Truong, H., McClanahan, T., Gorman, D.M., and Oft, M. (2012). IL-10 directly activates and expands tumor-resident CD8(+) T cells without de novo infiltration from secondary lymphoid organs. Cancer Res. *72*, 3570–3581. https://doi.org/10.1158/0008-5472.CAN-12-0721.

26. Qiao, J., Liu, Z., Dong, C., Luan, Y., Zhang, A., Moore, C., Fu, K., Peng, J., Wang, Y., Ren, Z., et al. (2019). Targeting tumors with IL-10 prevents dendritic cell-mediated CD8(+) T cell apoptosis. Cancer Cell *35*, 901–915.e4. https://doi.org/10.1016/j.ccell.2019.05.005.

27. Ryan, D., and Frezza, C. (2021). IL-10-mediated refueling of exhausted T cell mitochondria boosts anti-tumour immunity. Immunometabolism *3*, e210030. https://doi.org/10.20900/immunometab20210030.

28. Guo, Y., Xie, Y.Q., Gao, M., Zhao, Y., Franco, F., Wenes, M., Siddiqui, I., Bevilacqua, A., Wang, H., Yang, H., et al. (2021). Metabolic reprogramming of terminally exhausted CD8(+) T cells by IL-10 enhances anti-tumor immunity. Nat. Immunol. *22*, 746–756. https://doi.org/10.1038/s41590-021-00940-2.

29. Mumm, J.B., Emmerich, J., Zhang, X., Chan, I., Wu, L., Mauze, S., Blaisdell, S., Basham, B., Dai, J., Grein, J., et al. (2011). IL-10 elicits IFN-gamma-dependent tumor immune surveillance. Cancer Cell *20*, 781–796. https://doi.org/10.1016/j.ccr.2011.11.003.

30. Duraiswamy, J., Turrini, R., Minasyan, A., Barras, D., Crespo, I., Grimm, A.J., Casado, J., Genolet, R., Benedetti, F., Wicky, A., et al. (2021). Myeloid antigen-presenting cell niches sustain antitumor T cells and license PD-1 blockade via CD28 costimulation. Cancer Cell *39*, 1623–1642.e20. https://doi.org/10.1016/j.ccell.2021.10.008.

31. Kersten, K., Hu, K.H., Combes, A.J., Samad, B., Harwin, T., Ray, A., Rao, A.A., Cai, E., Marchuk, K., Artichoker, J., et al. (2022). Spatiotemporal co-dependency between macrophages and exhausted CD8(+) T cells in cancer. Cancer Cell *40*, 624–638.e9. https://doi.org/10.1016/j.ccell.2022.05.004.

32. Muñoz-Garcia, J., Cochonneau, D., Télétchéa, S., Moranton, E., Lanoe, D., Brion, R., Lézot, F., Heymann, M.F., and Heymann, D. (2021). The twin cytokines interleukin-34 and CSF-1: masterful conductors of macrophage homeostasis. Theranostics *11*, 1568–1593. https://doi.org/10.7150/thno.50683.

33. Cramer, J.D., Burtness, B., Le, Q.T., and Ferris, R.L. (2019). The changing therapeutic landscape of head and neck cancer. Nat. Rev. Clin. Oncol. *16*, 669–683. https://doi.org/10.1038/s41571-019-0227-z.

34. Kok, V.C. (2020). Current understanding of the mechanisms underlying immune evasion from PD-1/PD-L1 immune checkpoint blockade in head and neck cancer. Front. Oncol. *10*, 268. https://doi.org/10.3389/fonc.2020.00268.

35. Johnson, D.E., Burtness, B., Leemans, C.R., Lui, V.W.Y., Bauman, J.E., and Grandis, J.R. (2020). Head and neck squamous cell carcinoma. Nat. Rev. Dis. Prim. *6*, 92. https://doi.org/10.1038/s41572-020-00224-3.

36. Tan, J.C., Indelicato, S.R., Narula, S.K., Zavodny, P.J., and Chou, C.C. (1993). Characterization of interleukin-10 receptors on human and mouse cells. J. Biol. Chem. *268*, 21053–21059.

37. Evrard, D., Szturz, P., Tijeras-Raballand, A., Astorgues-Xerri, L., Abitbol, C., Paradis, V., Raymond, E., Albert, S., Barry, B., and Faivre, S. (2019). Macrophages in the microenvironment of head and neck cancer: potential targets for cancer therapy. Oral Oncol. *88*, 29–38. https://doi.org/10.1016/j.oraloncology.2018.10.040.

38. Troiano, G., Caponio, V.C.A., Adipietro, I., Tepedino, M., Santoro, R., Laino, L., Lo Russo, L., Cirillo, N., and Lo Muzio, L. (2019). Prognostic significance of CD68(+) and CD163(+) tumor associated macrophages in head and neck squamous cell carcinoma: A systematic review and meta-analysis. Oral Oncol. *93*, 66–75. https://doi.org/10.1016/j.oraloncology.2019.04.019.

39. Kumar, A.T., Knops, A., Swendseid, B., Martinez-Outschoom, U., Harshyne, L., Philp, N., Rodeck, U., Luginbuhl, A., Cognetti, D., Johnson, J., and Curry, J. (2019). Prognostic significance of tumor-associated macrophage content in head and neck squamous cell carcinoma: A meta-analysis. Front. Oncol. *9*, 656. https://doi.org/10.3389/fonc.2019.00656.

40. Watermann, C., Pasternack, H., Idel, C., Ribbat-Idel, J., Brägelmann, J., Kuppler, P., Offermann, A., Jonigk, D., Kühnel, M.P., Schröck, A., et al. (2021). Recurrent HNSCC harbor an immunosuppressive tumor immune microenvironment suggesting successful tumor immune evasion. Clin. Cancer Res. *27*, 632–644. https://doi.org/10.1158/1078-0432.CCR-20-0197.

41. Cruz, I., Meijer, C.J., Walboomers, J.M., Snijders, P.J., and Van der Waal, I. (1999). Lack of MHC class I surface expression on neoplastic cells and poor activation of the secretory pathway of cytotoxic cells in oral squamous cell carcinomas. Br. J. Cancer *81*, 881–889. https://doi.org/10.1038/sj.bjc.6690780.

42. Ayers, M., Lunceford, J., Nebozhyn, M., Murphy, E., Loboda, A., Kaufman, D.R., Albright, A., Cheng, J.D., Kang, S.P., Shankaran, V., et al. (2017). IFN-gamma-related mRNA profile predicts clinical response to PD-1 blockade. J. Clin. Invest. *127*, 2930–2940. https://doi.org/10.1172/JCI91190.

43. Valpione, S., Mundra, P.A., Galvani, E., Campana, L.G., Lorigan, P., De Rosa, F., Gupta, A., Weightman, J., Mills, S., Dhomen, N., and Marais, R. (2021). The T cell receptor repertoire of tumor infiltrating T cells is predictive and prognostic for cancer survival. Nat. Commun. *12*, 4098. https://doi.org/10.1038/s41467-021-24343-x.

44. Postow, M.A., Manuel, M., Wong, P., Yuan, J., Dong, Z., Liu, C., Perez, S., Tanneau, I., Noel, M., Courtier, A., et al. (2015). Peripheral T cell receptor diversity is associated with clinical outcomes following ipilimumab treatment in metastatic melanoma. J. Immunother. Cancer *3*, 23. https://doi.org/10.1186/s40425-015-0070-4.

45. Andreatta, M., and Carmona, S.J. (2021). UCell: Robust and scalable single-cell gene signature scoring. Comput. Struct. Biotechnol. J. *19*, 3796–3798. https://doi.org/10.1016/j.csbj.2021.06.043.

46. Wang, L.X., Talebian, F., Liu, J.Q., Khattabi, M., Yu, L., and Bai, X.F. (2012). IL-10 contributes to the suppressive function of tumour-associated myeloid cells and enhances myeloid cell accumulation in tumours. Scand.

J. Immunol. *75*, 273–281. https://doi.org/10.1111/j.1365-3083.2011.02662.x.

47. Li, B., Ren, M., Zhou, X., Han, Q., and Cheng, L. (2020). Targeting tumor-associated macrophages in head and neck squamous cell carcinoma. Oral Oncol. *106*, 104723. https://doi.org/10.1016/j.oraloncology.2020.104723.
48. Peltanova, B., Raudenska, M., and Masarik, M. (2019). Effect of tumor microenvironment on pathogenesis of the head and neck squamous cell carcinoma: a systematic review. Mol. Cancer *18*, 63. https://doi.org/10.1186/s12943-019-0983-5.
49. Cohen, E.E.W., Soulières, D., Le Tourneau, C., Dinis, J., Licitra, L., Ahn, M.J., Soria, A., Machiels, J.P., Mach, N., Mehra, R., et al. (2019). Pembrolizumab versus methotrexate, docetaxel, or cetuximab for recurrent or metastatic head-and-neck squamous cell carcinoma (KEYNOTE-040): a randomised, open-label, phase 3 study. Lancet *393*, 156–167. https://doi.org/10.1016/S0140-6736(18)31999-8.
50. Burtness, B., Harrington, K.J., Greil, R., Soulières, D., Tahara, M., de Castro, G., Jr., Psyrri, A., Basté, N., Neupane, P., Bratland, Å., et al. (2019). Pembrolizumab alone or with chemotherapy versus cetuximab with chemotherapy for recurrent or metastatic squamous cell carcinoma of the head and neck (KEYNOTE-048): a randomised, open-label, phase 3 study. Lancet *394*, 1915–1928. https://doi.org/10.1016/S0140-6736(19)32591-7.
51. Ferris, R.L., Blumenschein, G., Jr., Fayette, J., Guigay, J., Colevas, A.D., Licitra, L., Harrington, K., Kasper, S., Vokes, E.E., Even, C., et al. (2016). Nivolumab for recurrent squamous-cell carcinoma of the head and neck. N. Engl. J. Med. *375*, 1856–1867. https://doi.org/10.1056/NEJMoa1602252.
52. Gkretsi, V., Stylianou, A., Papageorgis, P., Polydorou, C., and Stylianopoulos, T. (2015). Remodeling components of the tumor microenvironment to enhance cancer therapy. Front. Oncol. *5*, 214. https://doi.org/10.3389/fonc.2015.00214.
53. Khalaf, K., Hana, D., Chou, J.T.T., Singh, C., Mackiewicz, A., and Kaczmarek, M. (2021). Aspects of the tumor microenvironment involved in immune resistance and drug resistance. Front. Immunol. *12*, 656364. https://doi.org/10.3389/fimmu.2021.656364.
54. Tang, T., Huang, X., Zhang, G., Hong, Z., Bai, X., and Liang, T. (2021). Advantages of targeting the tumor immune microenvironment over blocking immune checkpoint in cancer immunotherapy. Signal Transduct. Targeted Ther. *6*, 72. https://doi.org/10.1038/s41392-020-00449-4.
55. Newman, A.M., Liu, C.L., Green, M.R., Gentles, A.J., Feng, W., Xu, Y., Hoang, C.D., Diehn, M., and Alizadeh, A.A. (2015). Robust enumeration of cell subsets from tissue expression profiles. Nat. Methods *12*, 453–457. https://doi.org/10.1038/nmeth.3337.
56. Huang da, W., Sherman, B.T., and Lempicki, R.A. (2009). Systematic and integrative analysis of large gene lists using DAVID bioinformatics resources. Nat. Protoc. *4*, 44–57. https://doi.org/10.1038/nprot.2008.211.
57. Subramanian, A., Tamayo, P., Mootha, V.K., Mukherjee, S., Ebert, B.L., Gillette, M.A., Paulovich, A., Pomeroy, S.L., Golub, T.R., Lander, E.S., and Mesirov, J.P. (2005). Gene set enrichment analysis: a knowledge-based approach for interpreting genome-wide expression profiles. Proc. Natl. Acad. Sci. USA *102*, 15545–15550. https://doi.org/10.1073/pnas.0506580102.
58. Cassetta, L., Fragkogianni, S., Sims, A.H., Swierczak, A., Forrester, L.M., Zhang, H., Soong, D.Y.H., Cotechini, T., Anur, P., Lin, E.Y., et al. (2019). Human tumor-associated macrophage and monocyte transcriptional landscapes reveal cancer-specific reprogramming, biomarkers, and therapeutic targets. Cancer Cell *35*, 588–602.e10. https://doi.org/10.1016/j.ccell.2019.02.009.
59. Charoentong, P., Finotello, F., Angelova, M., Mayer, C., Efremova, M., Rieder, D., Hackl, H., and Trajanoski, Z. (2017). Pan-cancer immunogenomic analyses reveal genotype-immunophenotype relationships and predictors of response to checkpoint blockade. Cell Rep. *18*, 248–262. https://doi.org/10.1016/j.celrep.2016.12.019.
60. Kuo, W.Y., Lin, J.J., Hsu, H.J., Chen, H.S., Yang, A.S., and Wu, C.Y. (2018). Noninvasive assessment of characteristics of novel anti-HER2 antibodies by molecular imaging in a human gastric cancer xenograft-bearing mouse model. Sci. Rep. *8*, 13735. https://doi.org/10.1038/s41598-018-32094-x.
61. Kuchenbecker, L., Nienen, M., Hecht, J., Neumann, A.U., Babel, N., Reinert, K., and Robinson, P.N. (2015). IMSEQ–a fast and error aware approach to immunogenetic sequence analysis. Bioinformatics *31*, 2963–2971. https://doi.org/10.1093/bioinformatics/btv309.
62. Andreatta, M., Corria-Osorio, J., Müller, S., Cubas, R., Coukos, G., and Carmona, S.J. (2021). Interpretation of T cell states from single-cell transcriptomics data using reference atlases. Nat. Commun. *12*, 2965. https://doi.org/10.1038/s41467-021-23324-4.
63. Goldman, M.J., Craft, B., Hastie, M., Repečka, K., McDade, F., Kamath, A., Banerjee, A., Luo, Y., Rogers, D., Brooks, A.N., et al. (2020). Visualizing and interpreting cancer genomics data via the Xena platform. Nat. Biotechnol. *38*, 675–678. https://doi.org/10.1038/s41587-020-0546-8.
64. Cerami, E., Gao, J., Dogrusoz, U., Gross, B.E., Sumer, S.O., Aksoy, B.A., Jacobsen, A., Byrne, C.J., Heuer, M.L., Larsson, E., et al. (2012). The cBio cancer genomics portal: an open platform for exploring multidimensional cancer genomics data. Cancer Discov. *2*, 401–404. https://doi.org/10.1158/2159-8290.CD-12-0095.

STAR★METHODS

KEY RESOURCES TABLE

REAGENT or RESOURCE	SOURCE	IDENTIFIER
Antibodies		
Pacific Blue^{TM} anti-mouse CD45 Antibody (30-F11)	BioLegend	Cat#103126; RRID: AB_493535
APC anti-mouse CD3 Antibody (17A2)	BioLegend	Cat#100236; RRID: AB_2561456
APC anti-mouse/human CD11b Antibody (M1/70)	BioLegend	Cat#101212; RRID: AB_312795
FITC anti-mouse Ly-6C Antibody (HK1.4)	BioLegend	Cat#128006; RRID: AB_1186135
PerCP anti-mouse CD4 Antibody (RM4-5)	BioLegend	Cat#100432; RRID: AB_893323
PE anti-mouse CD8a Antibody (53–6.7)	BioLegend	Cat#100708; RRID: AB_312747
PerCP anti-mouse F4/80 Antibody (BM8)	BioLegend	Cat#123126; RRID: AB_893483
FITC anti-mouse CD279 (PD-1) Antibody (29F.1A12)	BioLegend	Cat#135214; RRID: AB_10680238
APC/Cy7 anti-mouse IFN-r Antibody (XMG1.2)	BioLegend	Cat#505850; RRID: AB_2616698
PE anti-mouse F4/80 Antibody (BM8)	BioLegend	Cat#123110; RRID: AB_893486
BV570 anti-mouse CD45 Antibody (30-F11)	BioLegend	Cat#103136; RRID: AB_2562612
BB515 anti-mouse/human CD11b Antibody (M1/70),	BD Biosciences	Cat#564454; RRID:AB_2665392
APC/Cy7 anti-mouse CD19 Antibody (6D5)	BioLegend	Cat#115530; RRID: AB_830707
APC/Cy7 anti-mouse CD3e Antibody (145-2C11)	BD Biosciences	Cat#557596; RRID: AB_396759
APC/Cy7 anti-mouse-NK1.1 Antibody (PK136)	BioLegend	Cat#108724; RRID: AB_830871
BV650 anti-mouse Ly6G Antibody (1A8)	BD Biosciences	Cat#740554; RRID: AB_2740255
BB700 anti-mouse F4/80 Antibody (T45-2342)	BD Biosciences	Cat#746070; RRID: AB_2743450
PE anti-mouse CD206 Antibody (C068C2)	BioLegend	Cat#141706; RRID: AB_10895754
BV605 anti-mouse I-A/I-E Antibody (M5/114.15.2)	BioLegend	Cat#107639; RRID: AB_2565894
BV421 anti-mouse CD11c Antibody (N418)	BD Biosciences	Cat#565452; RRID: AB_2744278
BV480 anti-mouse NK1.1 Antibody (PK136)	BD Biosciences	Cat#746265; RRID: AB_2743597
BV650 anti-mouse CD3e Antibody (145-2C11)	BD Biosciences	Cat#564378; RRID: AB_2738779
R718 Anti-mouse CD4 Antibody (RM4-5)	BD Biosciences	Cat#566939; RRID: AB_2869957
PE-/Dazzle594 anti-mouse CD25 Antibody (PC61)	Biolegend	Cat#102048; RRID: AB_2564124
AF647 anti-mouse FoxP3 Antibody (MF23)	BD Biosciences	Cat#560401; RRID: AB_1645201
BB700 anti-mouse CD44 Antibody (IM7)	BD Biosciences	Cat#566506; RRID: AB_2744396
BV605 anti CD8a Antibody (53–6.7)	Biolegend	Cat#100744; RRID: AB_2562609
PE-Cy7 CD279/PD-1 Antibody (RMB1-30)	BD Biosciences	Cat#748265; RRID: AB_2872693
BV711 anti-mouse CD366 Antibody (5D12)	BD Biosciences	Cat#747622; RRID: AB_2744188
BV421 anti-Granzyme B Antibody (GB11)	BD Biosciences	Cat#563389; RRID: AB_2738175
PE anti-mouse TCF1/TCF7 (C63D9) Rabbit mAb	Cell Signaling	Cat#14456; RRID: AB_2798483
Ki-67 antibody (D3B5)	Cell Signaling	Cat#9129; RRID: AB_2687446
CD45 (D3F8Q) Rabbit mAb	Cell Signaling	Cat#70527; RRID: AB_2799780
CD4 (D7D2Z) Rabbit mAb	Cell Signaling	Cat#25229; RRID: AB_2798898
CD8α (D4W2Z) Rabbit mAb	Cell Signaling	Cat#98941; RRID: AB_2756376
F4/80 (D2S9R) Rabbit mAb	Cell Signaling	Cat#70076; RRID: AB_2799771
Granzyme B (D6E9W) Rabbit mAb	Cell Signaling	Cat#46890; RRID: AB_2799313
PD-1 (D7D5W) XPR Rabbit mAb	Cell Signaling	Cat#84651; RRID: AB_2800041
FoxP3 (D6O8R) Rabbit mAb	Cell Signaling	Cat#12653; RRID: AB_2797979
CD19 (D4V4B) Rabbit mAb	Cell Signaling	Cat#90176; RRID: AB_2800152
TIM-3 (D3M9R) Rabbit mAb	Cell Signaling	Cat#83882; RRID: AB_2800033
In VivoMab anti-mouse CD8α (2.43)	BioXCell	BE0061; RRID: AB_1125541
In VivoMab rat IgG2b isotype control (LTF-2)	BioXCell	BE0090; RRID: AB_1107780

(*Continued on next page*)

Continued		
REAGENT or RESOURCE	SOURCE	IDENTIFIER
In VivoMab anti-mouse PD1 (RMP1-14)	BioXCell	BE0146; RRID: AB_10949053
In VivoMab rat IgG2a isotype control (2A3)	BioXCell	BE0089; RRID: AB_1107769
In VivoMab anti-mouse Ly6G/Ly6C (NIMP-R14)	BioXCell	BE0320; RRID: AB_2819047
Biological samples		
14 slide specimens form 6 HNSCC patients for Digital spatial profiling of RNA analysis (Figures 1K–1M, 1N, and S1J; Tables S3A and S3B)	Taipei Veterans General Hospital	TVGH-IRB certificate No.2018-06-001BC
44 tumor samples with 21 normal counterparts from 21 HNSCC patients for RNA-seq analysis (Figures S1C–S1H; Table S2)	Taipei Veterans General Hospital	TVGH-IRB certificate No.2017-05-013AC
Mouse tumor biopsies for flow cytometry analysis (Figure 4)	This paper	NYCU, IACUC No. 1081014rr, 1081205, 1100713, 1110915
Mouse tumor biopsies for Bulk RNA sequencing (Figures 5A–5G)	This paper	NYCU, IACUC No. 1081014rr, 1081205, 1100713, 1110915
Mouse tumor biopsies for single cell RNA sequencing (Figure 6)	This paper	NYCU, IACUC No. 1081014rr, 1081205, 1100713, 1110915
Mouse tumor biopsies for T cell receptor (TCR) sequencing (Figures 5K and S5F)	This paper	NYCU, IACUC No. 1081014rr, 1081205, 1100713, 1110915
Mouse serum for cytokine array (Figures 7I, 7J, S9E, and S9F)	This paper	NYCU, IACUC No. 1081014rr,1081205, 1100713, 1110915
Chemicals, peptides, and recombinant proteins		
Recombinant human IgG1 Fc (Human Fc-G1)	BioXCell	BE0096
Recombinant human IL10-Fc	Elixiron Immunotherapeutics Inc.	Customized
anti-mouse CSF1R (AFS98)	Elixiron Immunotherapeutics Inc.	Customized
IL10/anti-mCSF1R fusion protein (BF10)	Elixiron Immunotherapeutics Inc.	Customized
IL10/anti-hCSF1R fusion protein (huAb#1,2)	Elixiron Immunotherapeutics Inc.	Customized
Recombinant human IL10-Histag	Elixiron Immunotherapeutics Inc.	Customized
Critical commercial assays		
VivoTag 680XL Protein labeling Kit	Perkin Elmer	NEV11118
LEGEND MAX Human IFN-γ ELISA Kit	BioLegend	430107
LEGEND MAX Human Granzyme B ELISA Kit	BioLegend	439207
BD Cytofix/cytoperm	BD Biosciences	554714
Fc Receptor binding reagent	ebioscience	16-9161-73
Novolink Max Polymer Detection Systems	Leica Biosystems	RE7280-K
Opal 7 Color Manual IHC Kit	Akoya Biosciences	NEL811001KT
Opal 480 Reagent Pack	Akoya Biosciences	FP1500001KT
Opal 780 Reagent Pack	Akoya Biosciences	FP1501001KT
Opal Polymer anti-Rabbit HRP Kit	Akoya Biosciences	ARR1001KT
Tumor Dissociation Kit, mouse	Miltenyi Biotec	130-096-730
Dead Cell Removal Kit	Miltenyi Biotec	130-090-101
Quant-iT RNA BR Assay Kit	Thermo Fisher	Q10213
TruSeq Stranded mRNA Library Prep Kit	Illumina Inc	RS-122-2101
QIAseq Immune Repertoire RNA Library Kit	QIAGEN	333705
Chromium Next GEM Single Cell 3′ Reagent Kit (v3.1)	10x Genomics	PN-1000128
CD8a+ T cell isolation kit, mouse	Miltenyi Biotec	130-104-075
CD8a (Ly-2) MicroBeads, mouse, positive selection	Miltenyi Biotec	130-177-044

(*Continued on next page*)

Continued

REAGENT or RESOURCE	SOURCE	IDENTIFIER
CD45 MicroBeads, mouse	Miltenyi Biotec	130-052-301
XFp Cell Mito Stress Test Kit	Seahorse Bioscience	103010–100
Real-Time ATP Rate Assay Kit	Seahorse Bioscience	103591–100
XFp FluxPak	Seahorse Bioscience	103022–100
Chromium Next GEM Single Cell 3′ Reagent Kit (v3.1)	10x Genomics	PN-1000128
Deposited data		
TCGA RNA sequence data (HNSCC patients)	This paper	https://portal.gdc.cancer.gov/projects/TCGA-HNSC
TCGA RNA sequence data (BRCA patients)	This paper	https://portal.gdc.cancer.gov/projects/TCGA-BRCA
TCGA RNA sequence data (COAD patients)	This paper	https://portal.gdc.cancer.gov/projects/TCGA-COAD
Cohort analysis of patient survival data (TCGA-HNSC, TCGA-BRCA, and TCGA-COAD); Figures 1C, 1O, and S1K	This paper	https://xenabrowser.net/
TVGH RNA sequence data (HNSCC patients), 44 tumor samples with 21 normal counterparts from 21 HNSCC patients	This paper	GEO: GSE178537 (Figures 1K, 1L, 1M, 1N, S1C–S1H, and S1J; Tables S2 and S3)
Mouse RNA sequence data (Tumor tissue; HNSCC/Q1-2 mixed)	This paper	GEO: GSE193054 (Figures 5B–5F; Tables S5 and S6)
Mouse RNA sequence data (Tumor tissue; BRCA/4T1)	This paper	GEO: GSE193045 (Figure 5H; Tables S7 and S8)
Mouse RNA sequence data (Tumor tissue; HNSCC/Q1-2, 3 triplex)	This paper	GEO: GSE193051 (Figure S5A)
CD45-nagative cells sequence data (Tumor tissue; HNSCC/Q1-2)	This paper	GEO: GSE193050 (Figures S6A and S6B)
Single cell sequence data of CD45^{+} cells of Ctrl, IL10, antiCSF1R and BF10-treated mouse model (Tumor tissue; HNSCC/Q1-2, Orthotopic)	This paper	GEO: GSE190111 (Figure 6)
TCR repertoire sequence data of Ctrl, IL10, antiCSF1R and BF10-treated mouse model (Tumor, Spleen and tdLN tissues; HNSCC/Q1-2)	This paper	GEO: GSE216119 (Figures 5K, 5L, S5F, and S5G; Table S9)
Experimental models: Cell lines		
Mouse: MTC-Q1 (HNSC/Q1-2_Luc)	This paper	N/A
Mouse: 4T1 (4T1-Luc2)	ATCC	CRL-2539
Experimental models: Organisms/strains		
Mouse: C57BL/6JNarl	National Laboratory Animal Center, Taipei, Taiwan	N/A
Mouse: BALB/cByJNarl	National Laboratory Animal Center, Taipei, Taiwan	N/A
MuScreen^{TM} efficacy assay	CrownBio Inc.	N/A
Software and algorithms		
CIBERSORT	Alizadeh Lab (Newman et al. 2015)[55]	https://cibersort.stanford.edu/
GraphPad Prism 8	GraphPad Software, San Diego, CA	https://www.graphpad.com/
DAVID Bioinformatics Resources 6.8	LHRI (Huang et al., 2009)[56]	https://david.ncifcrf.gov/
Ingenuity Pathway Analysis (IPA)	QIAGEN	https://digitalinsights.qiagen.com/
Gene Set Enrichment Analysis (GSEA)	UC San Diego and Broad Institute (Subramanian et al., 2005)[57]	https://www.gsea-msigdb.org/gsea/index.jsp
ImmGen	ImmGen Groups	http://www.immgen.org/

(*Continued on next page*)

Continued

REAGENT or RESOURCE	SOURCE	IDENTIFIER
CellKb	Combinatics Inc.	https://www.cellkb.com/
Cell Ranger v3.0	10x Genomics Inc.	https://www.10xgenomics.com/
Loupe Browser 5.0	10x Genomics Inc.	https://www.10xgenomics.com/
Rstudio	N/A	https://rstudio.com/
Seurat v3.0	N/A	https://satijalab.org/seurat/
ProjecTILs v1.0	N/A	https://rdrr.io/github/carmonalab/ProjecTILs
Phenochart	Akoya Biosciences	https://www.akoyabio.com/phenoptics/software/
inForm Tissue Analysi Software	Akoya Biosciences	https://www.akoyabio.com/phenoptics/software/

RESOURCE AVAILABILITY

Lead contact

Further information and requests for resources, reagents, and samples should be directed to the Lead Contact, Muh-Hwa Yang (mhyang2@nycu.edu.tw).

Materials availability

The materials and reagents used in this study are listed in the key resources table. Reagents generated in our laboratory in this study or previous studies are available upon request.

Data and code availability

- Bulk RNA-seq and single-cell RNA-seq data have been deposited at GEO and are publicly available under accession numbers (GEO: GSE193051, GSE193045, GSE193054, GSE190111, GSE193050, and GSE216119). The survival and gene expression data of TCGA HNSCC cohort were based on UCSC Xena dataset[63] (https://portal.gdc.cancer.gov/projects/TCGA-HNSC) and cBioPortal (https://xenabrowser.net/).[64]
- This paper does not report original code.
- Any additional information required to reanalyze the data reported in this work paper is available from the lead contact upon request.

EXPERIMENTAL MODEL AND SUBJECT DETAILS

Human HNSCC samples

The clinical samples and studies conducted in this research received approval from the Institutional Review Board (IRB) of Taipei Veterans General Hospital (TVGH IRB No. 2014-03-004AC, 2017-05-013AC, 2018-06-00). Informed consent was obtained from all HNSCC patients before sample collection. Individual information was de-identified according to IRB-approved protocols. A total of 65 samples were collected from 21 patients and subjected to bulk RNA sequencing. The patient cohort consisted of 21 male individuals with ages ranging from 35 to 75 years. All patients had a pathological diagnosis of squamous cell carcinoma of the head and neck and had not undergone any prior anticancer treatment. Detailed clinical characteristics of the 21 HNSCC patients is presented in Table S2.

Mice

C57BL/6J and BALB/c mice (6–8 weeks old) were purchased from the National Laboratory Animal Center (Taipei, Taiwan) and were housed in a pathogen-free environment (50% humidity and 22°C). Animal experiments were conducted according to the Guide for the Care and Use of Laboratory Animals and were approved by the Institutional Animal Care and Utilization Committee of National Yang Ming Chiao Tung University (IACUC certificate No. 1081014rr, 1081205, 1100713 and 1110915). All mice were acclimated for 3 to 7 days before the experiment. We employed a mouse orthotopic model using oral cancer cells (MTCQ1-2 abbreviated as Q1-2) derived from tongue carcinogenesis. To ensure compliance with the principles of the 3Rs (Replacement, Reduction, and Refinement) in animal research, we made a deliberate decision to administer the orthotopic tumor inoculation via buccal mucosa injection. This choice was guided by two primary considerations. Firstly, direct inoculation in the tongue was observed to significantly disrupt the feeding behavior of the mice, which could potentially introduce confounding factors into the study. Secondly, by opting for buccal

CellPress
OPEN ACCESS

mucosa injection, we aimed to create a microenvironment that more closely resembled the natural conditions of the tongue. This approach allowed us to conduct experiments with greater feasibility while still preserving the essential characteristics of the oral cavity.

Cell lines

Mouse breast cancer cell lines (4T1, CRL-2539) were purchased from the American Type Culture Collection (ATCC). HNSCC/Q1-$2^{Lucferiease}$ is a luciferase-expressing subline from parental MTC-Q1 (mouse oral cancer cell line), which was kindly provided by Dr. Kuo-Wei Chang (Department of Dentistry, National Yang Ming Chiao Tung University of Taiwan). All cell lines were cultured in Dulbecco's modified Eagle's medium (DMEM) supplemented with 10% FBS (HyClone) and penicillin/streptomycin (1% v/v, Gibco) at 37°C in 5% CO_2.

METHOD DETAILS

Bioinformatics analysis of The Cancer Genome Atlas (TCGA) and VGH_HNSCC patients

The TCGA RNA sequencing (RNA-Seq) data and clinical characteristics of patients were downloaded from the Genomic Data Commons (GDC) Portal (https://portal.gdc.cancer.gov) and UCSC Xena database (https://xenabrowser.net/). The analysis contains nine different cancer types, including head and neck squamous carcinoma (HNSCC), breast cancer (BRCA), colon adenocarcinoma (COAD), lung adenocarcinoma (LUAD), lung squamous cell carcinoma (LUSC), prostate adenocarcinoma (PRAD), liver hepatocellular carcinoma (LIHC), skin cutaneous melanoma (SKCM), and kidney renal clear cell carcinoma (KIRC). Differential gene expression (DEG) was normalized to FPKM or log2 (norm_count+1). The immune cell composition was estimated by gene expression data using CIBERSORT analysis, which quantifies the proportions of immune cells among different cancer types (https://cibersort.stanford.edu/). For comparison with TCGA_HNSCC data, we also enrolled sequencing data from Taipei Veterans General Hospital (VGH_HNSCC; 65 primary or metastatic tumors from 21 patients; the characteristics of the patients are shown in Table S2). For survival analysis, we categorized TCGA_HNSCC patients into high- and low-level groups according to the expression levels of the highest and lowest quartiles of the candidate genes. Individual information for the patients was de-identified under Institutional Review Board (IRB)-approved protocols of Taipei Veterans General Hospital (VGH-IRB certificate No. 2014-03-004AC; No. 2017-05-013AC; No. 2018-06-001BC). The immune cell type and gene list have been described in detail[58,59] and were used for the cell score correlation of CSF-1R and IL-10/IL10-RA axis.

Digital spatial profiling (DSP) of RNA analysis in HNSCC samples

The FFPE slides (5 μm) from HNSCC specimens were used for GeoMx DSP analysis. All protocols were performed with instrument manual (NanoString, Seattle WA) and as described (Merritt et al., 2020). Briefly, fresh-cut tissue sections were stained with multiplex cocktail oligonucleotides probes (UV-photocleavable) and four fluorescent markers against epithelial cells (Pan-cytokeratin), T cells (CD3e), macrophages (CD68), and cell nuclei (DNA GeoMx Nuclear Stain) to visualize tumor tissue architecture and cell morphology, followed by scanning with GeoMx DSP instrument. Spatial images and segmented regions of interest (ROIs) were generated from a total of 171 ROIs, each with a diameter of 500 μm, across 14 slides obtained from six patients with HNSCC. The accuracy and validity of these segmented ROIs were confirmed through careful assessment by a pathologist. Next, selected ROIs were UV-illuminated and released indexing oligonucleotides were collected for NanoString optical barcodes hybridization and nCounter sequencer analysis. The Cancer Transcriptome Atlas (CTA) which targets ∼1800 genes across tumor biology and immune response, was enrolled for selected ROIs analysis. The outlier probes were dropped from downstream data analysis. Then, the data were processed by Q3 normalization. Individual counts were normalized against the 75th percentile of the signal from their own ROI. The clinical characteristics of the 6 patients for DSP study is shown in Table S3A. The RNA values of the ROIs are listed in Table S3B.

Tumor model and treatment

In tumor growth studies, PBS-resuspended cancer cells (5×10^5 of Q1-2 or 5×10^4 of 4T1) were mixed 1:1 (v/v) with BD Matrigel (#354234) and were inoculated into mice by orthotopic or subcutaneous injection as indicated in the figures. Tumor growth was measured twice a week using a digital caliper, and the treatment started when the tumor size reached 100 mm^3. Tumor-bearing mice received intraperitoneal treatment with IL-10-Fc (20 mg/kg), anti-CSF-1R (30 mg/kg), BF10 (36 mg/kg) or control IgG (30 mg/kg) for a total of 4–6 doses in different experiments. All mice received the equivalent dose treatment according to different molecular weights of antibody components. The molecular weights of IL10-Fc, anti-CSF-1R and anti-CSF1R-IL10 (BF10) were 92 kDa, 150 kDa, and 185.5kDa, respectively. After tumor injection (20–36 days), the mice were sacrificed, and tumor samples were collected for flow cytometry assays, immunohistochemistry, immunofluorescence, and RNA sequencing. The survival analysis was defined as death spontaneously within one month or a tumor burden size reaching 1500 mm^3 or length >1.5 cm.

MuScreen examination of BF10 antitumor activity in syngeneic tumor models

The CrownBio MuScreen platform was utilized to examine the antitumor activity of BF10 in other syngeneic mouse models of different cancer types. Briefly, BALB/c mice (6–8 weeks old, female) were implanted subcutaneously with 5×10^5 CT26 (ATCC CRL-2638), 5×10^5 EMT6 (ATCC CRL-2755), 1×10^6 H22 (RRID:CVCL_H613), or 1×10^6 Renca cells (ATCC CRL-2947). C57BL/6 mice (6–8 weeks old, female) were implanted subcutaneously with 3×10^5 LL2 (ATCC CRL-1642), 1×10^6 MC38 (RRID:CVCL_B288), or 3×10^6 Pan02 cells (RRID:CVCL_D627). Mice were randomized into treatment groups when the tumor volume reached 50–100 mm^3. Mice were then

injected intraperitoneally twice weekly with 36 mg/kg anti-CSF1R/IL-10 fusion protein. Tumor volume was measured twice per week by caliper measurements until the end of the study. The percentage of tumor growth inhibition (TGI %) was calculated from the ratio of volume change in treatment and control between Day 0 and the end after final dosing.

Generation of bifunctional anti-CSF-1R/IL-10 fusion proteins

The bifunctional anti-CSF-1R/IL-10 fusion proteins were designed with the goal of delivering IL-10 to TAM-rich tumors while blocking CSF-1R signaling and boosting the antitumor $CD8^+$ T cell response. For mouse experiments, recombinant anti-CSF-1R/IL-10 fusion protein (BF10) was designed by genetically fusing human IL-10 polypeptide to the C-terminus of CSF-1R antibody (clone AFS98) of mouse IgG2a, which was separated by a 14-amino-acid linker. The human recombinant bifunctional fusion protein was designed by genetically fusing human IL-10 to the N-terminus of human IgG1 Fc fragments separated by a 14-amino-acid linker. The desired gene segments, preceded by an IL-2 secretion sequence required for secretion of recombinant proteins, were obtained using the Thermo gene synthesis service and cloned into a mammalian expression vector. A bifunctional antibody was generated by transient transfection of the expression plasmids into ExpiCHO-S cells (Thermo Scientific), and the supernatant containing the fusion protein was purified using Protein A Sepharose Beads (GE Healthcare) according to the manufacturer's protocol. The quality of the purified antibodies was examined by SDS–PAGE in the presence and absence of a reducing agent and stained with Coomassie blue.

ELISA of BF10 binding activity and neutralization

Recombinant human CSF1R-His (Biolegend), human CSF1R-Fc, mouse CSF1R-Fc (Sino Biological), or mouse/human IL-10Ra fusion protein (R&D Systems) were immobilized on 96-well microtiter plates overnight at 4°C. The wells were washed with wash solution (0.05% Tween 20 in imidazole-buffered saline) and blocked with 1% BSA. Serial dilutions of the antibodies or Ab/IL-10 fusion proteins were added to the wells and incubated at 37°C for 1 h. For binding ELISA, peroxidase-conjugated goat anti-human kappa light chain antibody (Sigma) was applied for 1 h at 37°C. For competition ELISA, biotinylated CSF1-His (Biolegend) was applied for 1 h at 37°C, and then streptavidin-HP (Jackson ImmunoResearch) was applied for 1 h at RT. After washing, the wells were developed with TMB substrate, and the reaction was stopped with 1 N HCl. Thereafter, the absorbance was measured at 450 nm and 650 nm. The EC50 and IC50 values were calculated using GraphPad Prism 8.

Inhibition of CSF-1-dependent cell survival of macrophages by anti-CSF1R antibodies

Human peripheral blood was obtained from healthy donors. Peripheral blood mononuclear cells (PBMCs) were immediately isolated by density gradient centrifugation using Ficoll-Paque Plus (GE Healthcare). $CD14^+$ monocytes were isolated by using anti-human CD14 conjugated magnetic beads (Miltenyi Biotec). To generate monocyte-derived macrophages, human $CD14^+$ monocytes (2×10^6 cells/mL) were cultured in culture medium (RPMI-1640 containing 10% FBS) supplemented with 100 ng/mL CSF-1 for 6 days. In the proliferation assay, differentiated macrophages ($2{\times}10^4$ per well) were incubated with serial dilutions of antibodies in culture medium in 96-well plates. CSF-1 (10 ng/mL) was then added to the cells. Cell survival was measured 72 h after stimulation using a CellTiter-Glo assay (Promega).

IL10R-STAT3 reporter assay

The biological activity of the bifunctional BF10 protein was determined by the STAT3 reporter assay. HeLa cells overexpressing IL-10RA were seeded into a 96-well plate at a concentration of 8000 cells/well in 0.1 mL complete medium (DMEM containing 10% FBS) at 37°C for 4 h. Serial dilutions of the indicated components of IL-10-Histag, IL10-Fc, anti-CSF1R antibody, and BF10 were added to the cells and cultured for 18 h. Luciferase activity was measured using the ONE-Glo Luciferase Assay (Promega) according to the manufacturer's protocol. The calculated curve of one representative data of three independent experiments was presented as mean ± S.D. All data analyses were performed using GraphPad Prism (San Diego, CA).

$CD8^+$ T cell proliferation assay

A macrophage-mediated suppression assay on $CD8^+$ T cell proliferation was performed as described previously with modifications (27). $CD8^+$ T cells were isolated from the spleens of C57BL/6/J mice using a negative selection Mojosort kit (BioLegend) and labeled with 5 mg/mL CFSE (Life Technology) for 10 min at 37°C protected from light. Cells were washed twice and resuspended in medium containing 10 ng/mL murine IL-2 (Peprotech) and 10 ng/mL murine CSF-1 (Peprotech). Then, cells were seeded on wells coated with anti-CD3 and anti-CD28 antibodies (2 mg/mL) to allow activation alone or at a ratio of 2:1 (2-CD8T:1-BMDM) with BMDMs previously treated for 24 h with 10 ng/mL IL-4 (Peprotech). During coculture, either CSF-1R Ab (CD115, Invitrogen), rIL-10-Fc or BF10 was added at a concentration of 50 ng/mL. After 72 h, CD8 T cells were collected, stained and analyzed by flow cytometry.

Seahorse metabolomics analysis

The metabolic effects of BF10-treated $CD8^+$ T cells were examined by using a Seahorse XF Cell Mito stress Kit (Agilent Technologies) according to the manufacturer's instructions. In brief, isolated splenic $CD8^+$ T cells from tumor-bearing mice were seeded in pre-coated XF24 culture plate, followed by incubation with XF RPMI medium containing pyruvate (1 mM), glutamine (2 mM), and glucose (5 mM). After 37°C incubation (30 min, non-CO2 condition), The assay plate with $CD8^+$ T cells was treated with BF10 (0.05 mg/mL) for

30 min, then sequentially addition of the seahorse assay reagents of Oligomycin (1 μM), FCCP (0.5 μM) and Rot/antimycin A (1 μM) to examine the oxygen consumption rate (OCR) and extracellular acidification rate (ECAR) of T cells.

Serum pharmacokinetics of BF10

The *in vivo* retention time curve of BF10, IL10-Fc and IL10-tag were assessed using a single intravenous bolus injection in mice. Mice were received the equivalent dose treatments of BF10 (20 mg/kg), IL10-Fc (10 mg/kg) or IL10-tag (5 mg/kg) using intravenous administration, then followed by collecting peripheral blood (10 μL blood) at the indicated time (0.25, 1, 2, 4, 8, 24, 48, 72 h). The collected bloods were immediately diluted with 1X PBS (1:9), then centrifuged (300g, 5min) and store the supernatant until IL10 ELISA assay.

Preparation of ^{111}In-labeled BF10 and the animal SPECT/CT imaging of ^{111}In-labeled BF10

The diethylenetriaminepentaacetic acid (DTPA)-modified antibodies were prepared as previously reported.[60] Briefly, a 20-fold molar excess of *p*-SCN-Bn-DTPA dissolved in bicarbonate buffer (0.05M, pH = 9.2) was added to a reaction vial containing the antibodies. The mixture was reacted at 37°C for 2 h. After the reaction, the crude product was loaded onto a 10-kDa membrane column and centrifuged at 10,000 g for 10 min to remove the unreacted chelate. The centrifugation step was repeated twice for complete removal. For radiolabeling, ^{111}In-InCl$_3$ (37 MBq) was loaded to a vial containing 40 μL of sodium citrate buffer (0.1 M, pH = 5.0) and then DTPA-conjugated antibodies (0.1 mg) were added. The reaction mixture was kept at 40°C for 20 min. The radiolabeling efficiencies and radiochemical purities of the ^{111}In-labeled BF10 were determined by radio-thin layer chromatography (radioTLC), which was performed on an instant TLC plate (ITLC, Merck, NewJersy, USA) using sodium citrate buffer (0.5 M, pH = 5.5) as the mobile phase, by using a scanner (AR2000, Bioscan, Washington, USA). The animal SPECT/CT images were acquired using the nanoSPECT/CT imaging modality (Mediso, Hungary) at Cheng Gung Memorial Hospital, Taoyuan City, Taiwan. Static imaging was performed for 30 min at 24 h after intravenous injection of 18.5 MBq of ^{111}In-DTPA-BF10. Standard uptake values (SUVs) of tumor and muscle were calculated using the AMIDE software (Version 1.0.5). The tumor-to-muscle ratio (*T/M*) was applied to eliminate the individual difference.

Biodistribution studies of ^{111}In-labeled BF10 and the blood clearance kinetic of ^{111}In-labeled BF10

The tumor-bearing mice were sacrificed at 24 h post-injection of 18.5 MBq of ^{111}In-labeled BF10 (with carrier; total 500 μg). Samples of blood, heart, lung, liver, stomach, intestines, spleen, kidney, lymph node, muscle, and tumor were excised, cleaned, and weighed. The radioactivity of samples was determined by a gamma counter (Wizard 2, PerkinElmer Inc., USA). The tissue uptake was expressed as a percentage of the injected dose per gram of sample (%ID/g). The 1.85–3.7 MBq of ^{111}In-labeled BF10 (in 150 μL normal saline) were injected via orbital sinus and the blood samples were collected from the tail vein with 1 μL of micro-capillary tubes at 0.17, 0.5, 1, 2, 20, 24, 48, and 72 h post-administration. The radioactivity of blood samples was measured by a 2470 Wizard Gamma counter (PerkinElmer, Waltham, MA, USA). Data were expressed as the percentage injected dose per milliliter (%ID/mL). The area under the curve (AUC) and half-life ($T_{1/2}$) of the curve were estimated by Prism (version 9.2.0).

Activation of CD8$^+$ T cells by IL-10

Human CD8$^+$ T cells were isolated from PBMCs using CD8 magnetic beads (Miltenyi Biotec). Human tumor-infiltrating lymphocytes (TILs) were isolated from TNBC patient biopsy samples (National Taiwan University Hospital, IRB No: 2014121193RINC). Isolated CD8$^+$ T cells and TILs were activated with T cell TransAct (Miltenyi Biotec) for 3 days. Following activation, T cells were treated with Ab/IL-10 fusion proteins for 3 days and restimulated with anti-CD3 (Biolegend) for 4 h. Concentrations of IFN-γ and granzyme B in cell culture media were measured by ELISA (Biolegend) according to the manufacturer's instructions.

Antibody labeling, biodistribution and *in vivo* imaging

Purified BF10 fusion proteins were labeled using a near-infrared (NIR) fluorochome labeling Kit (VivoTag 680XL, PerkinElmer) according to the manufacturer's instructions. HNSC/Q1-2^{Lucferiease} tumor cells were subcutaneously inoculated into syngeneic mice (C56BL/6) for tumor growth. After a 2-week growth period, HNSC/Q1-2^{Lucferiease} tumor-bearing mice were intravenously injected with VivoTag680-labeled BF10 (150 μg) for 16 h followed by *in vivo* bioluminescence and fluorescence detection using a Xenogen IVIS 100 imaging system. For the biodistribution of BF10, mouse tissues with VivoTag680 signals, including tumor, spleen and tumor-driving lymphoid node (tdLN), were isolated to confirm the existence of BF10 by fluorescence examination with a Vectra Polaris Imaging system (Akoya Biosciences).

Immunohistochemistry (IHC)

The IHC experiments were performed using a Novolink polymer detection system kit (# RE7150-K, Lecia Biosystem) according to the manufacturer's protocol. Briefly, formalin-fixed, paraffin-embedded (FFPE) tumor sections (5 μm thick) were deparaffinized and rehydrated, followed by antigen retrieval with Tris-EDTA solution (pH 9.0). After washing with PBS-T (0.05% Tween 20) thee times, sample slides were treated with Peroxidase Block and Protein Blocking reagents (Lecia Biosystem) before overnight primary antibody incubation (1:250 for CD8 #98941; 1:1000 for Ki-67 #9129, both from Cell Signaling Technology). Next, the slides were incubated with horseradish peroxidase (HP)-conjugated polymer (30 min, RT), followed by diaminobenzidene (DAB) development and counterstaining with Mayer hematoxylin. The serial slices were also stained with hematoxylin and eosin for histologic examination. Images were captured at 20X magnification using an Olympus microscope system (Olympus B×51; Olympus Corp., Japan).

In vivo depletion of CD8$^+$ T cells or macrophages

For CD8$^+$ T cell depletion, 200 μg anti-mouse CD8α antibody (clone 2.43, BioXcell) or isotype control (rat IgG2b, BioXcell) was intraperitoneally (i.p.) injected into mice every three days four times in the presence or absence of BF10 cotreatment. The depletion efficacy of CD8 T cells was examined by flow cytometry analysis of mouse peripheral blood samples (pre-bleed and post-bleed) and IHC as indicated. The macrophage depletion was accomplished by intraperitoneal administration of Clodronate-Liposome (Liposoma BV, Netherlands) as our previous study.[3] Briefly, Tumor-bearing mice were received with Clodronate- and/or PBS- liposome solution (200μL contains 1mg clodronate for 20g mice) at the indicated time, followed by co-treatment of IL-10-Fc or BF10 with a three-day interval for tumor growth analysis.

Flow cytometry assays

Mouse samples of tumor-infiltrating leukocytes (TILs) and peripheral blood cells were collected to analyze immune cell composition by flow cytometric analysis. For TILs, tumors tissues were minced into small pieces and incubated in RPMI containing 2% FBS, DNase I (1 μg mL^{-1}; Sigma-Aldrich), and collagenase (0.5 mg mL^{-1}; Sigma-Aldrich) for 30 min at 37 °C. After cells were filtered with a 70-μm cell strainer, filtered cells were incubated with RBC Lysing Buffer (Biolegend) to lyse red blood cells and then washed with FACS buffer (phosphate-buffered saline with heat-inactivated 2% FBS and 0.1% sodium azide). TILs were further enriched by Percoll density gradient centrifugation at 800*g* for 30 min. Fc receptors on cells were blocked with anti-CD16/32 antibody on ice for 15 min before staining. Viable cells were determined using a BD Horizon Fixable Viability Stain 450 reagent (BD Biosciences) at room temperature for 15 min. Cells were processed for surface marker staining and then intracellular molecule staining. Samples were analyzed on Cytek Aurora or LSRII flow cytometers (BD Biosciences), and data were analyzed with FlowJo v10. The following antibodies were used for flow cytometry: anti-CD3ε (17A2), anti-CD4 (RM4-5), anti-CD8a (53.6.7), anti-CD11b (M1/70), anti-CD11c (N418), anti-CD19 (6D5), anti-CD45 (30-F11), anti-CD103 (2E7), anti-Ly6G (1A8), anti-MHC class II I-Ab/I-E (M5/114.15.2), anti-FoxP3 (MF-14), anti-NK1.1 (HP-3G10), anti-F4/80 (T45-2342), anti-CD44 (IM7), anti-Tim3 (RMT3-23), anti-PD-1 (RMP1-30), and TCF1(C63D9). Besides anti-TCF1 (C63D9; Cell Signaling Technology), other antibodies were purchased from Biolegend or BD Biosciences. For peripheral blood samples, blood was drawn and mixed with ACK RBC lysis buffer (1:9) for 3 min of incubation. After centrifugation, cell pellets were washed, blocked with Fc blocker and resuspended in FACS buffer for surface marker staining (30 min on ice), then followed by flow cytometers analysis.

Multiplex immunofluorescence staining of mouse samples

FFPE sections of mouse tissues (tumor, spleen and tdLN) were processed as described in IHC analysis. For multiple marker staining, samples were analyzed using the Opal 7-Color manual IHC kit (NEL811001KT, Akoya Biosciences, Waltham, MA) according to the manufacturer's recommendations. The dilutions of primary antibodies were preoptimized by the signal intensity of IHC assays and then applied to the Opal IHC multiplex platform. These antibodies included CD4 (1:20, #25229), CD8a (1:250, #98941), F4/80 (1:250, #70076), Ly6G (1:500, #MBS-2556115), PD-1 (1:100, #846541), CD19 (1:1000, #90176), granzyme B (GZMB, 1:250, #46890), FoxP3 (1:200, #12653) and Ki-67 (1:1000, #9129). Briefly, epitope-retrieval tissue slides were washed twice with TBS-T, followed by blocking with a blocking/antibody diluent solution (10 min, RT, Akoya #ARD1001EA). Then, slides were incubated with primary antibody (4°C, overnight), followed by HP-conjugated polymer secondary for 10 min at RT. After washing with TBS-T twice, a single Opal fluorophore working solution (Opal 480, 520, 540, 570, 620 and 690 stock reagents) was prepared and further incubated with the slides for an additional 10 min for first-round Opal signal generation. Then, the primary antibody-HP polymer-Opal complex was removed by HIER treatment as described above for secondary antibody binding. The repeated staining steps and antibody-Opal complex removal were terminated until all Opal fluorophores were used. Finally, the tissue slides were mounted with Fluoroshield medium with DAPI (Sigma–Aldrich, #F6057). Images were acquired and processed with the Vectra Polaris Automated Quantitative Pathology Imaging System and inform tissue analysis software (Akoya Biosciences). All comparative images were obtained using identical area and camera settings. Detailed information on the antibodies used in the experiments is listed in Table S9.

Bulk RNA sequencing and biologic inference

To examine the effects of BF10 on the TME, tumor tissues were collected from murine breast cancer (4T1) and HNSCC (Q1-2) models treated with 4 doses of IL-10-Fc, anti-CSF-1R antibody, BF10 or control IgG for RNA sequencing. Briefly, total RNA was extracted from resected tumor tissues using the TRIzol protocol and quantified with a Quant-iT RNA BR assay kit (Thermo Fisher). RNA-Seq libraries were prepared using the TruSeq Stranded mRNA Library Prep Kit, followed by PCR with KAPA HiFi Polymerase according to the manufacturer's instructions. Libraries were sequenced using an Illumina NextSeq 550 system to generate 33–40 million reads per sample with a read length of 75 bp. Sequenced data were aligned to the mouse reference genome mm10 (GRCm38.101). Sequence reads were normalized to 10 million reads per sample and log2 transformed with the formula log2(((expression gene × ÷ library size)106)+1), where the library size was the sum of all expression values per sample. Gene expression differences with an FDR value of 0.05 or smaller and an expression difference ≥50% were considered statistically significant and determined as differentially expressed genes (DEGs). To clarify the possible biological pathways influenced by BF10, an online bioinformatics database (DAVID Bioinformatics Resources 6.8) was applied to analyze significant changes in DEGs (FC > 1.5 and $p < 0.05$; FC < 0.5 and $p < 0.05$). Ingenuity pathway analysis software (IPA, QIAGEN) was used to examine the functional connectivity of genes related to pathways in HNSC/Q-1 tumor samples between groups. Gene set enrichment analysis (GSEA) was performed to determine whether there were significant differences in the defined gene set between the 2 different treatment groups. The expanded immune gene signature was described as previously reported,[42]

and the expression score in this study was defined as the average expression level of *Cd3d, Ido1, Ciita, Cd3e, ccl5, Gzmk, Cd2, Cxcl13, Il2rg, Nkg7, Cxcr6, Lag3, Tagap, Cxcl10, Stat1* and *Gzmb*.

Generation of mouse TCR immune repertoire sequencing data

Total RNA of $CD8^+$ T cells was extracted as described in method of bulk RNA sequencing. The same amount RNA (100 ng and RIN >8) of indicated group was applied to construct RNA-seq libraries using QIAseq Mouse TCR Sequencing kit (#333705, Qiagen). Briefly, cDNA synthesis was completed with TCR-specific RT primers, which recognize the constant region of T cell receptor alpha, beta, gamma, and delta. Next, the cDNA molecule was assigned with a unique molecular index (UMI) by ligating a 12-base oligo adapter with sample index. Following UMI assignment and reaction cleanup, TCR target enrichment was performed by single primer extension, followed by processing with sample indexing primer and universal primer for libraries amplification. Finally, TCR libraries were sequenced using an Illumina NextSeq 550 system for CDR3 region. All protocols and steps were performed according to the manufacturer's instructions, reference report.[61] Samples were processed by the assistance of Cancer Progression Research Center (National Yang Ming Chiao Tung University) and analyzed data were completed by QIAseq Immune Repertoire RNA Library Kit Data Analysis Portal (https://geneglobe.qiagen.com/us/analyze). The relative clonotype information and diversity results from the QIAseq Immune Repertoire report were also evaluated using the Morisita-Horn index. This index was utilized to compare the similarity of treatment-induced differences across all groups and different tissues.

Single-cell RNA sequencing

The schematic protocol of scRNA-seq is shown in Figure 6A. Tumor tissues were harvested from the $HNSC/Q1\text{-}2^{Lucferiease}$ orthotopic cancer model with four doses of control-IgG or IL-10 or anti-CSF-1R and BF10. Single-cell suspensions were isolated with kits (mouse dissociation kit, RBC lysis solution, dead cell removal kit) and a gentleMACS dissociator. All of the above reagents/instruments were obtained from Miltenyi Biotech and followed the manufacturer's protocols. Next, $CD45^+$ tumor-infiltrating lymphocytes (TILs) and $CD45^-$ cells were separated using $CD45^+$ Microbeads and MACS LS columns (Miltenyi Biotech). The separated cells were washed twice with 1X PBS and resuspended in DMEM containing 5% FBS at a cell density of 1000 cells/μl. Cell suspensions were processed by drop-based scRNAseq using a Chomium Next GEM Single Cell 3′ Reagent Kit (v3.1 kit, 10x Genomics), followed by a reverse transcription reaction within GEM droplets containing one cell, one gel bead and reverse transcript reagents for cDNA library preparation. Single-cell libraries were sequenced using an Illumina NovaSeq 6000 System (Illumina Inc.). The sequenced reads were processed with the Cell Ranger pipeline (v5.01, 10x Genomics) and then mapped to the mouse genome (GENCODE vM23/Ensembl 98) using Cell Ranger software (10x Genomics) and further analyzed by Loupe Browser (10x Genomics). Low-quality cells and potential doublets were ruled out by mitochondrial counts (>10%) and feature values per gene (greater than 95% of the total samples or cells expressed fewer than 1000 genes). Dimensionality reduction and visualization were analyzed by the UMAP algorithm with the default settings of Loupe Browser. For T cell subsets, we evaluated the expression of T cell marker genes using the UCell package for gene signature scoring.[45] T cell subtype annotation was performed with ProjecTILs v1.0[62] using default parameters. Differential expression analysis for cells of a specific subtype was performed using the FindMarkers function of Seurat with default parameters; the results were displayed using the EnhancedVolcano package (https://github.com/kevinblighe/EnhancedVolcano) using a log2(fold-change) threshold of 1.

Neutrophil depletion

To deplete neutrophils, we administered 200μg of anti-mouse Ly6G/Ly6C antibody (clone NIMP-R14, BioXcell) intraperitoneally (i.p.). The effectiveness of neutrophil depletion was assessed through flow cytometry analysis of mouse peripheral blood samples (prebleed and post-bleed) and immunohistochemistry (IHC), as indicated. The experimental scheme and protocols were presented in Figure S10C. In brief, mice received either Ly6G/6C or Mock (Rat-IgG2b) treatment one day before the experiment, followed by co-treatment of PBS or BF10 with a three-day interval for tumor growth analysis.

Combination therapy of BF10 with anti-PD-1 blockade

A subcutaneous $HNSC/Q1\text{-}2^{Lucferiease}$ mouse model was used for the combination therapy. Tumor-bearing mice received an isotype control or anti-PD-1 (200 μg) and/or BF10 (36 mg/kg) when the average tumor size reached 150 mm^3. The antibody treatments of BF10 or isotype control (clone 2A3, BioXcell) or anti-mouse PD-1 (clone. RMP1-14, BioXcell) were intraperitoneally injected at 3-day intervals for a total of six doses. Tumor size was measured and recorded using a digital caliper.

Cytokine array assay

Mouse serum samples were analyzed using a Quantibody Mouse Cytokine Array 4000 Kit (QAM-CAA-4000, RayBiotech) to examine the expression levels of various cytokines according to the manufacturer's instructions. This combinational assay kit contains five glass array slides (Q4, Q5, Q6, Q7 and Q8) to quantitatively detect 200 mouse cytokines using a multiplexed sandwich ELISA platform. Briefly, serum samples were diluted 2-fold with PBS into wells and then incubated overnight at 4°C. Next, the slides were washed and incubated with a biotinylated antibody cocktail mixture at room temperature for 2 h, followed by further incubation with Cy3 dye-streptavidin reagent for 1 h. After 5 washes, the slides were detected with a laser scanner imaging system using the Cy3 channel (Axon Instruments, GenePix). The signal values of the spots were processed with RayBio analysis software, and each array contained internal controls to normalize the data.

QUANTIFICATION AND STATISTICAL ANALYSIS

All experiments were conducted with a minimum of two independent replicates, each consisting of more than four technical repeats. The data are presented as the mean ± standard deviation (S.D.), unless otherwise specified with standard error of the mean (S.E.M.). Statistical analysis for comparing differences between two groups was performed using a two-sided unpaired Student's t-test. Functional assays among the four treatment groups were evaluated using one-way ANOVA with post-hoc Tukey test. Pearson's correlation test was employed to analyze the correlation between two factors. Survival rates were assessed using the Kaplan-Meier curve and log rank test. All statistical analyses were conducted using GraphPad Prism (v8). Statistical significance was denoted as follows: * for $p \leq 0.05$, ** for $p \leq 0.01$, and *** for $p \leq 0.001$.

Cell Reports Medicine, Volume *4*

Supplemental information

A CSF-1R-blocking antibody/IL-10 fusion protein increases anti-tumor immunity by effectuating tumor-resident $CD8^+$ T cells

Yao-Wen Chang, Huey-Wen Hsiao, Ju-Pei Chen, Sheue-Fen Tzeng, Chin-Hsien Tsai, Chun-Yi Wu, Hsin-Hua Hsieh, Santiago J. Carmona, Massimo Andreatta, Giusy Di Conza, Mei-Tzu Su, Pandelakis A. Koni, Ping-Chih Ho, Hung-Kai Chen, and Muh-Hwa Yang

Supplemental information

The file includes:

-Supplementary Figure S1 to S11 with legends

A CSF-1R-blocking antibody/IL-10 fusion protein increases anti-tumor immunity by effectuating tumor-resident CD8^{+} T cells

Yao-Wen Chang, Huey-Wen Hsiao, Ju-Pei Chen, Sheue-Fen Tzeng, Chin-Hsien Tsai, Chun-Yi Wu, Hsin-Hua Hsieh, Santiago J. Carmona, Massimo Andreatta, Giusy Di Conza, Mei-Tzu Su, Pandelakis A. Koni, Ping-Chih Ho, Hung-Kai Chen, and Muh-Hwa Yang

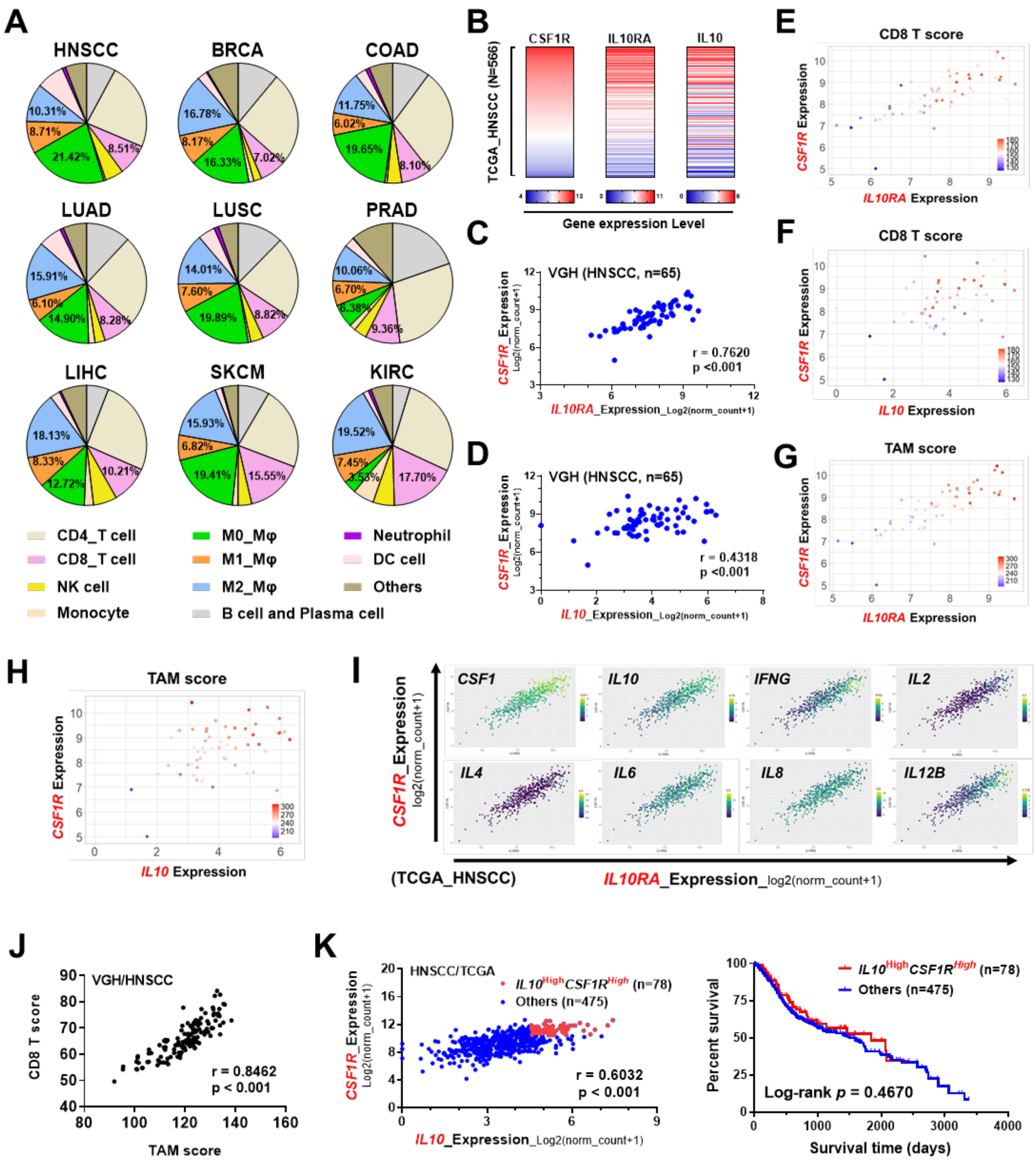

Figure S1. The immune cell composition in different cancers and the relationship between CSF-1R and IL10/IL10-RA axis and immune signature in HNSCC, related to Figure 1. (A) The proportion of infiltrated immune cells within tumor microenvironment. Bulk RNA-sequencing analysis from 9 cancer types of TCGA database. Relative abundance of immune cell types among different cancer types was analyzed by CIBERSORTx with LM22 signature. head and neck squamous cell carcinoma (HNSCC); breast cancer (BRCA); colon adenocarcinoma (COAD); lung adenocarcinoma (LUAD); lung squamous cell carcinoma (LUSC); prostate adenocarcinoma (PRAD); liver cancer (LIHC); skin cutaneous melanoma (SKCM); kidney renal clear cell carcinoma (KIRC). **(B)** The gene expression panels of CSF1R, IL-10RA and IL-10 of the individuals of TCGA HNSCC patients (n= 566). **(C),(D)** The correlation analysis of RNA expression level between IL-10/IL-10-RA axis and CSF-1R of head and neck squamous carcinoma (HNSCC) in Taipei Veterans General Hospital (VGH) database (n=65). The correlation was determined by

Pearson correlation (r). **(E)-(H)** The 3D scatter plots for analyzing the expression of CD8 T score and tumor -associated macrophages (TAM) score related to IL10/IL10RA axis and CSF1R in VGH-HNSC. X-axis represents IL10 or IL10-RA expression. Y-axis means CSF1R expression. CD8 T or TAM scores were expressed from blue to red gradient color dots (low to high). The value expressed by RNA level (log2(norm_count+1)). **(I)** The 3D scatter plots for analyzing the expression of different cytokine genes related to IL10-RA and CSF1R in TCGA-HNSC. **(J)** The correlation analysis of the gene signature scores between CD8 T and TAM of VGH/HNSCC DSP dataset. RNA expression level from 134 ROIs of 6 patients were assessed and Region of interests (ROIs) were categorized as peri-normal tissue (Normal), primary tumor (inner tumor and outer tumor) and metastatic lymph node (LN). The correlation was determined by Pearson correlation (r). **(K)** Left panel: a scatter plot of IL10 and CSF1R gene expression level in TCGA HNSCC patients. Red dots indicate the patients with the top 50% level of IL10 (IL-10^{High}) and top 50% of CSF1R (CSF1R^{High}) gene expression level. Right panel: survival analysis between groups of IL10^{High}CSF1R^{High} (red line) and others (blue line). P value was estimated by log-rank test.

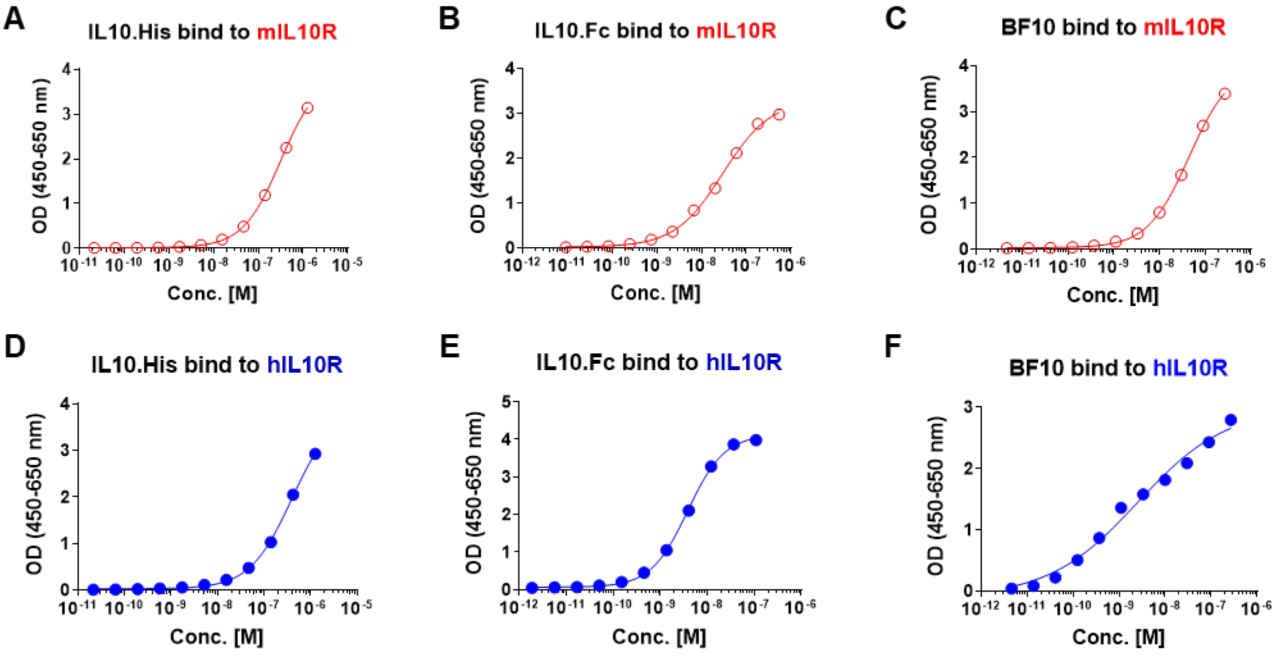

G

ELISA Bnding Assay (EC50 = M)			
Type	IL10	IL10.Fc	BF10
Bind to human IL-10RA	$3.95x10^{-7}$	$3.84x10^{-9}$	$2.55x10^{-9}$
Bind to mouse IL-10RA	$3.16x10^{-7}$	$3.00x10^{-8}$	$4.47x10^{-8}$

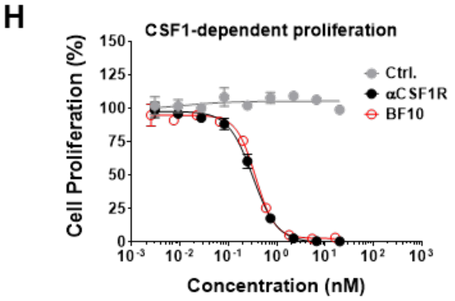

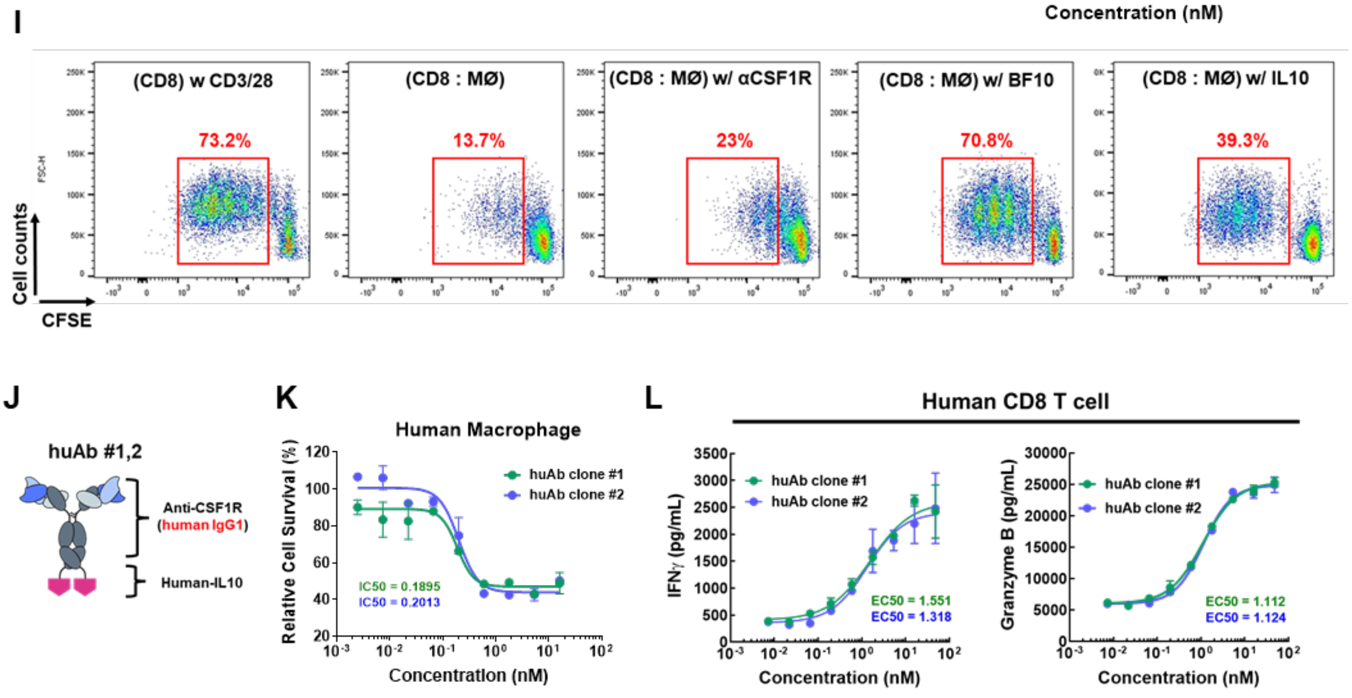

Figure S2. In vitro characterization of the α-CSF1R-IL10 fusion proteins, related to Figure 2. (A)-(F) Dose-response binding curve of IL-10-Histag, IL10-Fc and BF10 to recombinant mouse or human IL-10 receptor (a-c for mouse and d-f for human, respectively). The binding assay were performed by ELISA and data are calculated by OD values. **(G)** A table for summarizing the calculated EC50 values of BF10 compared to IL10-histag and IL10-Fc. EC50 values were calculated from three independent experiments. **(H)** Proliferation assay of M-NFS-60 murine myelogenous leukemia cells incubated with CSF-1 together with anti-mCSF-1R or BF10 or control for 3 days. Cell proliferation was measured using CellTiter-Glo assay (Promega). **(I)** Effects of

BF10 on T cell proliferation. CFSE-labeled $CD8^+$ T cells were activated with anti-CD3 and anti-CD28 in absence or presence of IL4-primed BMDMs. The activated cells were treated with 50 ng/ml of anti-CSF-1R, IL-10 or BF10 for 72 hours, and collected for flow cytometry analysis. The number indicates proliferating CD8 T cells percent in the red bracket. **(J)** Schematic presentation of the anti-human CSF-1R-IL10 fusion proteins (named as huAb # clone 1 and 2). **(K)** Cell viability assay**.** Human macrophages were derived from $CD14^+$ monocytes and then incubated with CSF-1 with different concentrations of anti-hCSF-1R-IL-10 fusion proteins for 3 days. Cell survival was measured using CellTiter-Glo assay (Promega). **(L)** Assay for the production of IFN-γ and granzyme B from human activated $CD8^+$ T cells. CD 8^+ T cells were isolated from PBMC and activated with anti-CD3 plus anti-CD 28 (T Cell TransAct Miltenyi Biotec) for 3 days and then treated for 3 days with anti CSF-1R/IL-10 fusion proteins and triggered with anti-CD3 (Biolegend) for 4 hours. The level of IFN-γ and granzyme B were measured by ELISA (Biolegend). The calculated curve of one representative data of three independent experiments was presented as mean ± S.D. and was shown in indicated assays in j and k, respectively.

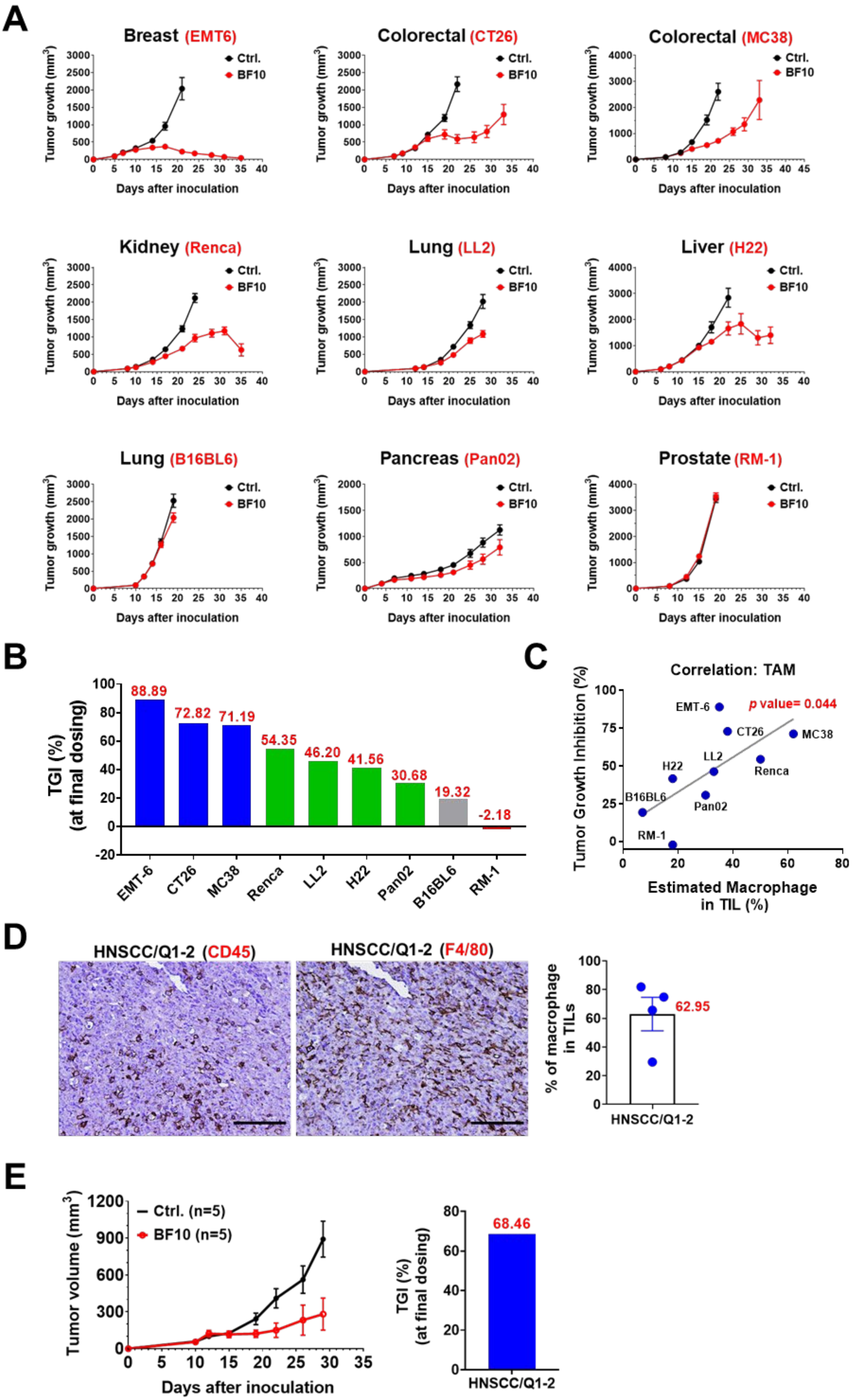
A
Breast (EMT6)
Colorectal (CT26)
Colorectal (MC38)
Kidney (Renca)
Lung (LL2)
Liver (H22)
Lung (B16BL6)
Pancreas (Pan02)
Prostate (RM-1)
Ctrl.
BF10
Tumor growth (mm3)
Days after inoculation
B
TGI (%) (at final dosing)
88.89
72.82
71.19
54.35
46.20
41.56
30.68
19.32
-2.18
EMT-6
CT26
MC38
Renca
LL2
H22
Pan02
B16BL6
RM-1
C
Correlation: TAM
Tumor Growth Inhibition (%)
p value= 0.044
Estimated Macrophage in TIL (%)
D
HNSCC/Q1-2 (CD45)
HNSCC/Q1-2 (F4/80)
% of macrophage in TILs
62.95
HNSCC/Q1-2
E
Tumor volume (mm3)
Ctrl. (n=5)
BF10 (n=5)
Days after inoculation
TGI (%) (at final dosing)
68.46
HNSCC/Q1-2

Figure S3. The antitumor activities of BF10 in mouse syngeneic tumor models, related to Figure 3. (A) The anti-tumor activity of BF10 was further assessed among night cancer cell lines for seven cancer types. The syngeneic mouse cancer platform (MuScreen^{TM}, CrownBio Inc.) was applied and analyzed with their responding macrophage proportion in the same model (CrownBio Inc.). Tumor-bearing mice were received the treatment with either isotype control or BF10 (36 mg/kg) for a total of six doses. All mice received the equivalent dose treatment as described in methods. The tumor growth was measured twice per week until end of the study. **(B)** The tumor growth inhibition (TGI) values (presented in %) among different cancer types. TGI was calculated from the ratio of volume change in treatment and control between Day 0 and the end of the experiment (Day 33). **(C)** The correlation analysis of TGI versus estimated macrophages numbers in tumor-infiltrated lymphocytes (TILs). The relationship was evaluated by linear regression model and shown as p-value. **(D)-(E)** The anti-tumor activity of BF10 for mouse HNSCC syngeneic model. HNSCC/Q1-2 tumor-bearing mice were received treatments of Ctrl-IgG or BF10 for six doses, and then tumor samples were harvested and subjected to determine the infiltration of $CD45^+$ and $F4/80^+$ cells by immunohistochemical staining. Representative images of the $CD45^+$ cells or $F4/80^+$ cells from the control group and BF10 group were calculated for macrophage proportion in TIL. The TGI value was calculated from the ratio of volume change in treatment and control between Day 0 and the end of the experiment (Day 29).

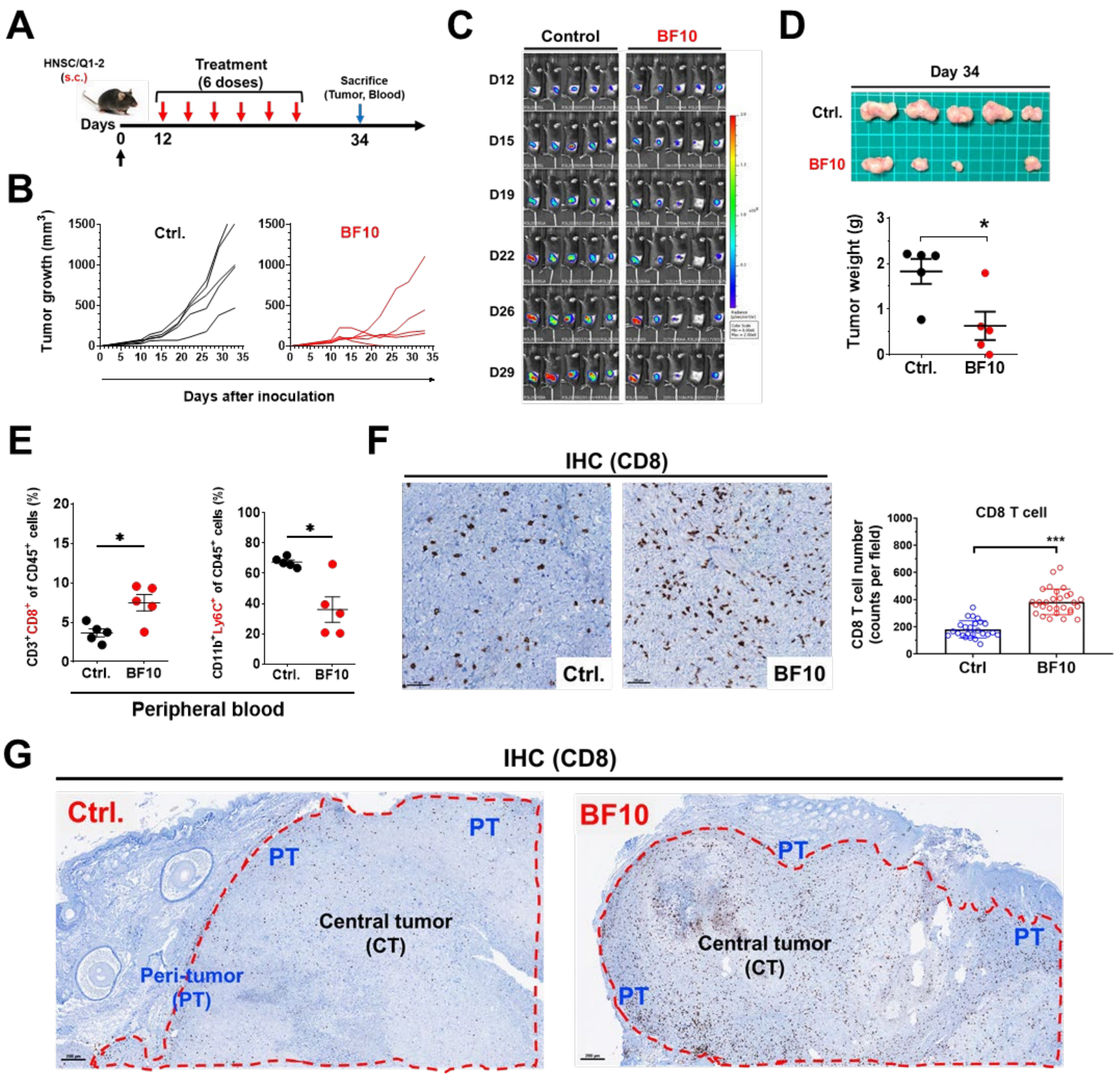

Figure S4. The antitumor activities of BF10 in mouse syngeneic tumor models, related to Figure 3. (A)-(F) Tumor-bearing mice (HNSCC/Q-2^{lucferiease}, subcutaneous) received control IgG (30 mg/kg) or BF10 (an equivalent dose of 30 mg/kg IgG) twice per week for a total of six doses when tumor size reach to 100 mm^3 (Day 12). At endpoint of treatment (Day 34), tumor tissues and serum samples were collected for analysis. **(A)** Experimental scheme and treatment protocol of mouse model of HNSCC cancer cells. **(B)** Tumor volume curves of individual mice. **(C)** Representative bioluminescence images for tumor growth. The bioluminescence intensity of tumor growth (Day 12, 15, 19, 22 26 and Day 29) was measured using Xenogen IVIS 100 imaging system. n=5 for each group. **(D)** Upper: representative photos of the collected tumors. Lower: quantification of tumor weights after treatment of BF10 at Day 34. *P < 0.05. **(E)** Flow cytometric analysis of the immune cells in peripheral blood of the tumor-bearing mice after last treatment (n = 5 per group). **(F)** The representative image of the tumor-infiltrated CD8$^+$ T cells with immunohistochemical (IHC) examination (Upper). Images were captured by a microscope system (Olympus BX51; Olympus Corp.). Scale bar= 50 µm. (Lower) The quantification of the CD8$^+$ T cells per HPF (20X magnification). Images from indicated groups (n= 3 to 4 mice, total images > 25 HPF) were quantified. Data were presented as mean ± S.D. **(G)** Representative images

of the distribution of tumor-infiltrated $CD8^+$ T cells. Tumor tissues (orthotopic murine HNSCC model) from the indicated treatments were examined by using IHC staining. Tumor tissue was separated into outer and inner regions which define as peri-tumor (PT, blue color) and central-tumor (CT, black color). Scale bar, 200 μm.

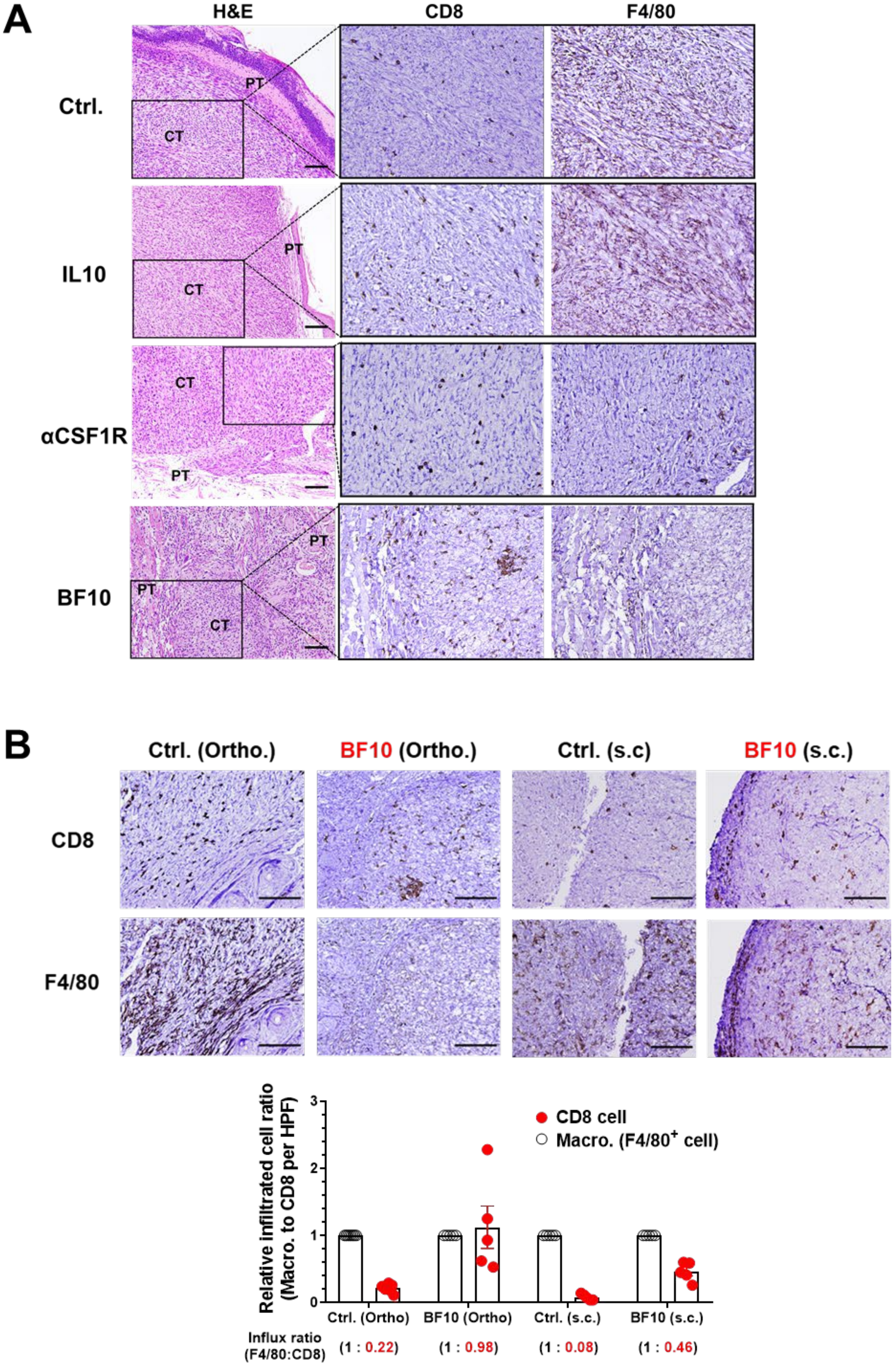

Figure S5. The antitumor activities of BF10 in mouse syngeneic tumor models, related to Figure 3. (A) Tumor-bearing mice (HNSCC/Q-2^{lucferiease}) received treatments of Ctrl-IgG (30 mg/kg), IL10-Fc (20 mg/kg), αCSF1R (30 mg/kg) or BF10 (36 mg/kg)

for six doses. Collected samples were assayed using immunohistochemical staining for CD8 T cells and tumor-resident macrophages. Representative images of H&E and IHC staining of $CD8^+$ T cells or $F4/80^+$ cells in tumor tissues from the indicated groups. n= 3 per group. Scale bar= 100 μm. **(B)** The ratio comparison of $CD8^+$: $F4/80^+$ cells between orthotopic and subcutaneous models of HNSCC/Q1-2 tumor cells. Representative image is one of at least two independent experiments. The quantification of the CD8+ T cells or macropahges per HPF (40X magnification). Images from indicated groups (n= 3 to 4 mice) were quantified. Data were presented as mean ± S.D. Scale bar = 100 μm

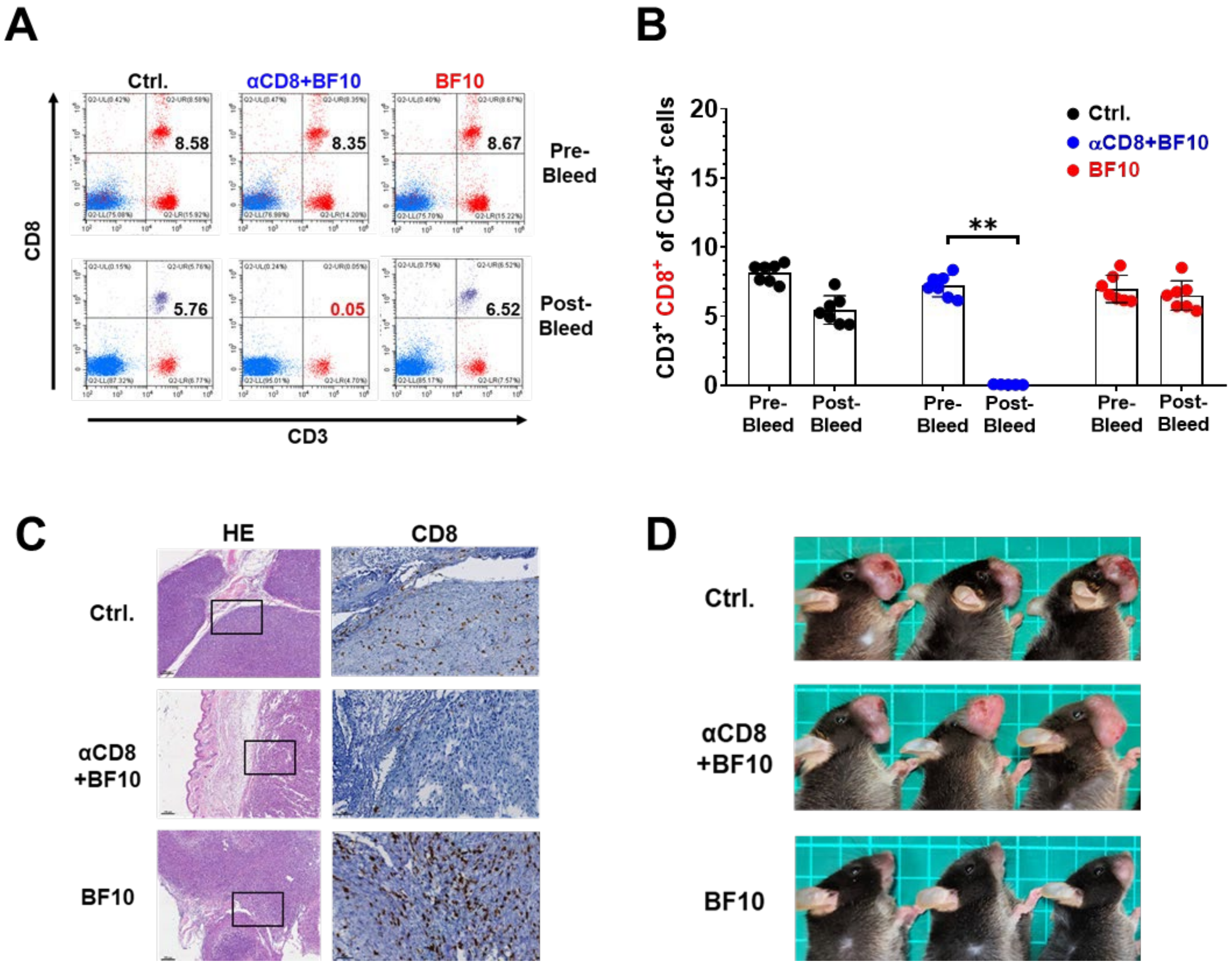

Figure S6. The antitumor activities of BF10 in mouse syngeneic tumor models, related to Figure 3. (A)-(B) $CD8^{+}$ T cells of peripheral blood of pre-bleed and post-bleed were assessed by flow cytometric analysis. The $CD8^{+}$ T cell depletion was carried out using 200 μg anti-CD8 antibody (i.p. every 3 days) simultaneously with or without BF10 treatment in tumor-bearing mice and protocols were described in Fig. 3I. **(C)** Representative images of H&E and IHC staining of $CD8^{+}$ T cells in tumor tissues from the indicated groups. n= 6-7 per group, **(D)** Representative photos of the orthotopic tumor from the indicated groups.

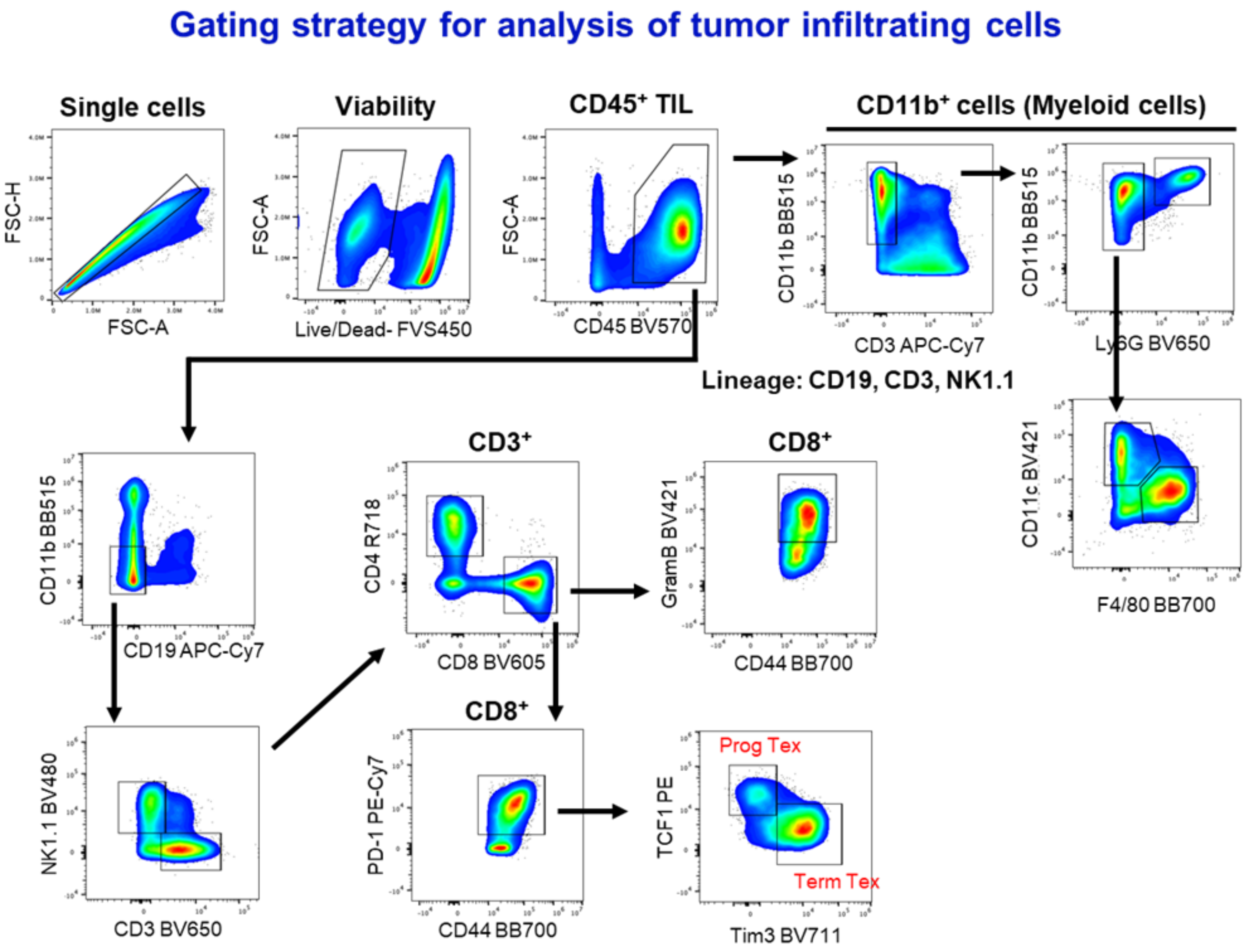

Figure S7. BF10 modulates immune cells in tumor and lymphatic organs, related to Figure 4. Gating strategies to assess immune compartmentalization of T cell subsets within tumor microenvironment**.** FACS gating strategy for immune cell profiling among tumors. The population and activity of infiltrated immune cells in the tumor microenvironment were determined according to the gating strategy.

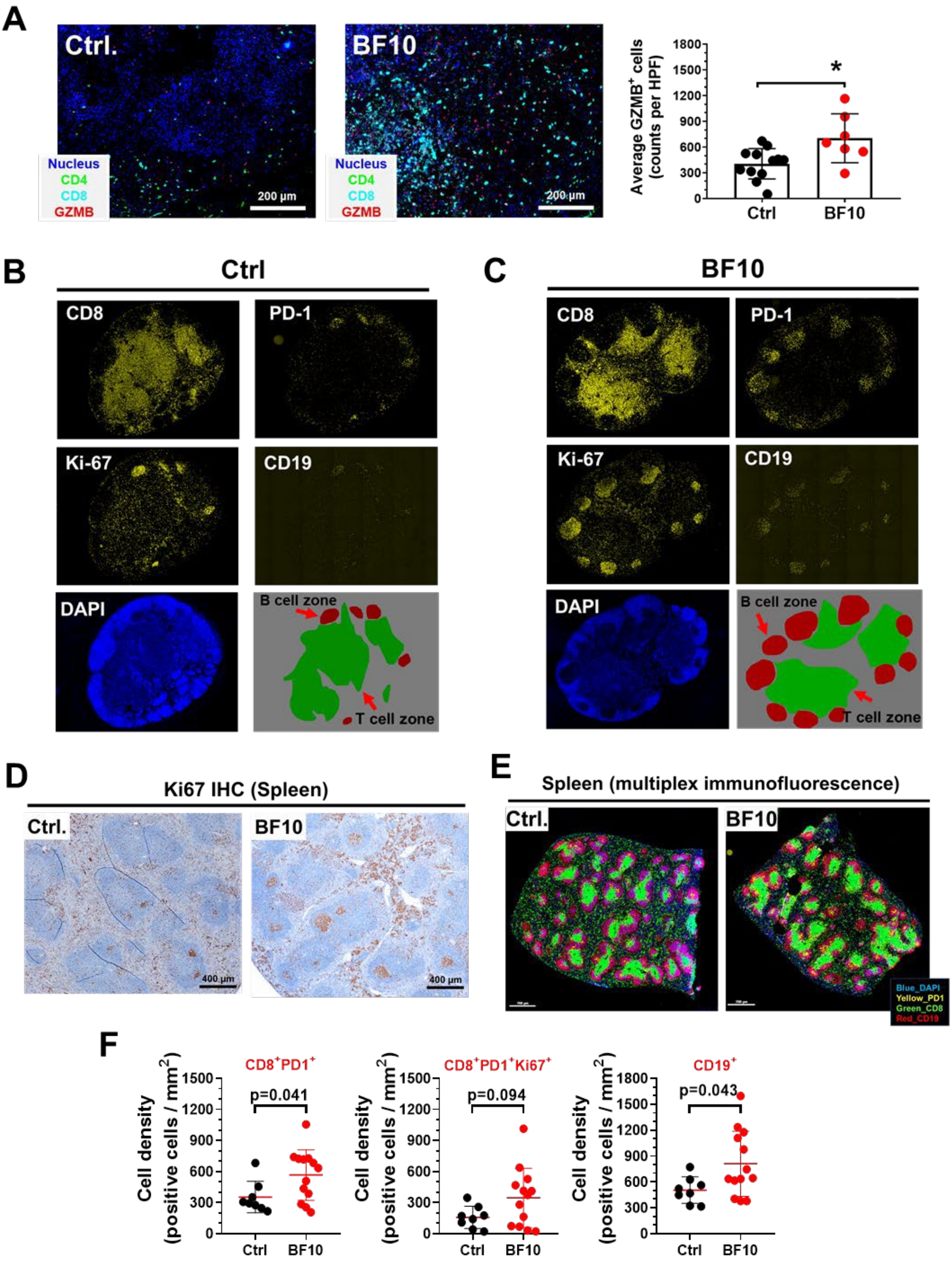
A
Ctrl.
BF10
Nucleus
CD4
CD8
GZMB
200 μm
Average GZMB+ cells (counts per HPF)
Ctrl
BF10
B
C
Ctrl
BF10
CD8
PD-1
Ki-67
CD19
DAPI
B cell zone
T cell zone
D
Ki67 IHC (Spleen)
400 μm
E
Spleen (multiplex immunofluorescence)
Blue_DAPI
Yellow_PD1
Green_CD8
Red_CD19
F
CD8+PD1+
CD8+PD1+Ki67+
CD19+
p=0.041
p=0.094
p=0.043
Cell density (positive cells / mm2)

Figure S8. BF10 modulates immune cells in tumor and lymphatic organs, related to Figure 4. (A) Representative immunofluorescence images of CD4 (Green), CD8 (sky blue) and $GZMB^+$ cells (Red) within central tumor regions. All images were captured and analyzed by Vectra Polaris Imaging system and Inform AI software. The average numbers of infiltrated $GZMB^+$ T cells were counted using Inform software analysis, Scale bar, 200 µm. **(B)-(C)** Representative multiplex immunofluorescence (mIF) images of tdLN stained with immune cells (see Figure 4J). The separated color for each marker was shown as indicated. **(D)** Representative IHC staining images of Ki67-expressing cells in spleens of indicated groups. Scale bar, 400 µm. **(E)** Representative multiplex immunofluorescence (mIF) images of spleens stained with immune cells. The assayed were performed by using Opal-dye staining kit (Perkin Elmer) which contains DAPI (blue), CD8 (green), PD-1 (yellow), and CD19 (Red). Scale bar, 700 µm. **(F)** Quantification of the immune cell density of tdLN in the BF10-treated vs. control group. Images and cell density were captured and analyzed with Vectra Polaris Imaging system and Inform software. *$P < 0.05$ and **$P < 0.01$. Unpaired student's t test.

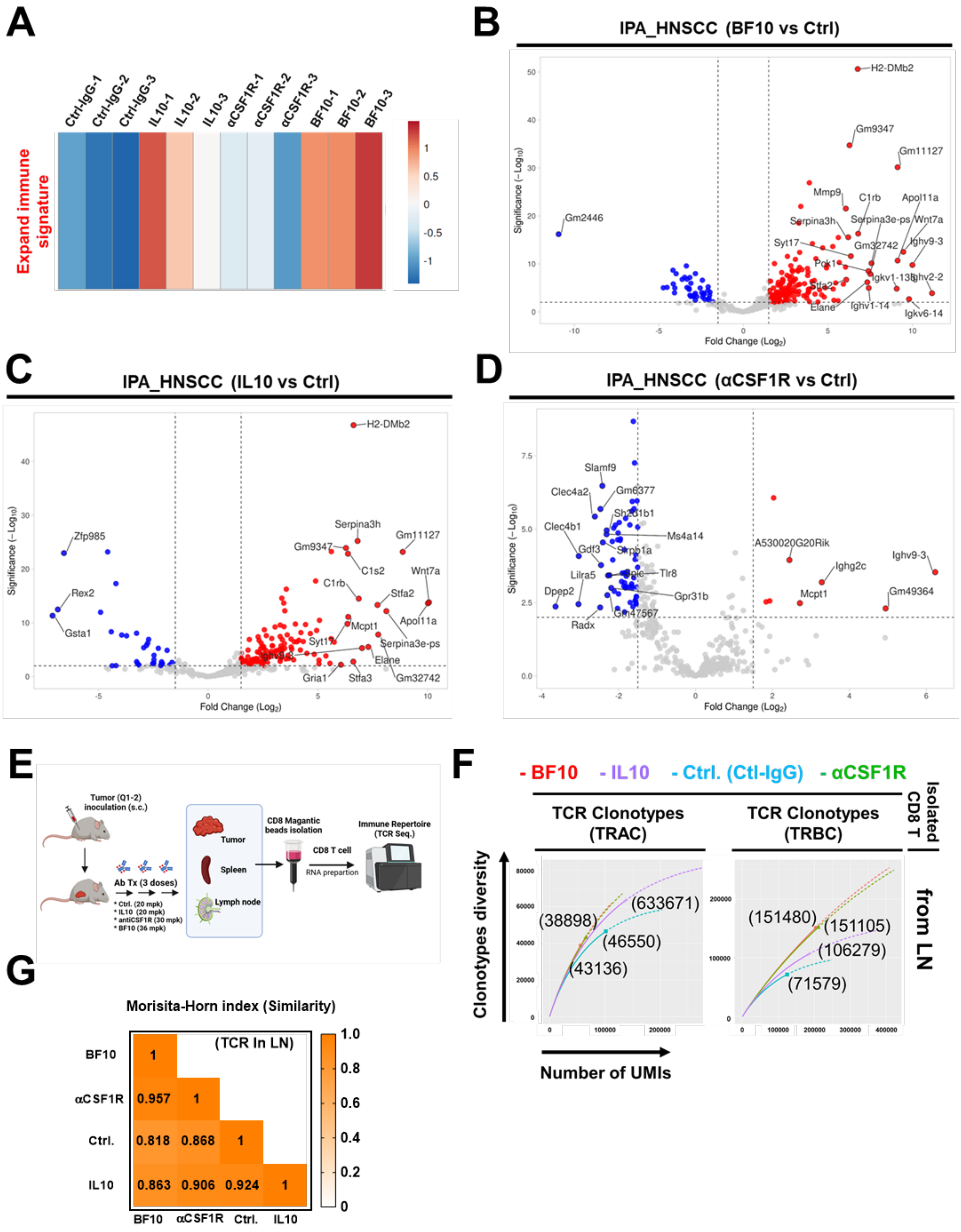

Figure S9. Transcriptomic and TCR repertoire analysis of the tumors or tdLNs treated with BF10, IL10-Fc, *α*CSF-1R, or control, related to Figure 5. (A) The heatmap of the expanded immune signatures in HNSCC/Q1-2 samples. Isolated tumor tissues of indicated treatment were applied to RNA extraction, followed by bulk RNA-Sequencing for gene expression profiles.

The signatures of expanded immune signature (18 genes) were analyzed by ssGSEA of gene set enrichment score for RNA-seq data (N=3 per group). **(B)-(D)** Volcano plot analysis of differential expressed genes (minus-log p-value) versus magnitude of expression change (log2 Fold Change) between pair comparison of treatments. BF10 vs. Ctrl-IgG **(B)**, IL-10 vs. Ctrl **(C)** and anti-CSF-1R vs. Ctrl **(D)**. **(E)** An illustration of mice received indicated components, followed by RNA extraction from $CD8^{+}$ T cells for T cell receptor (TCR) immune repertoire analysis. **(F)** T cell receptor (TCR) immune repertoire analysis of tumor-driving lymph nodes (tdLN). The clonotype diversity and distribution of both TRAC and TRBC CDR3 sequencing were assessed**.** The number of the bracket indicates observed diversity in the enrichment metrics of QIAseq-RNA Immune Repertoire Application. **(G)** Examination of the treatment-induced difference in TCR immune repertoire using Morista-Horn index.

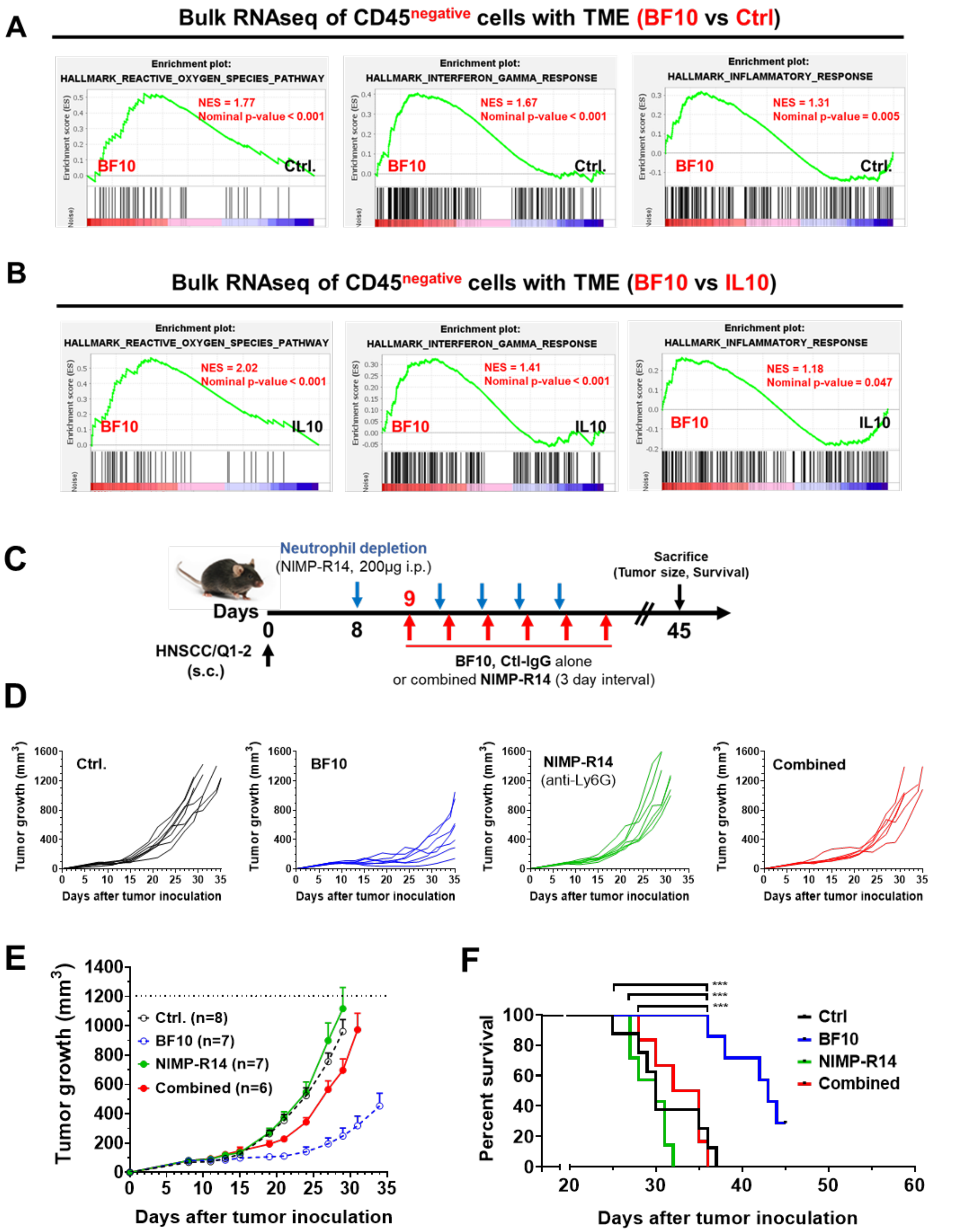

Figure S10. Gene set enrichment analysis of the bulk RNA-seq data of the CD45$^-$ cells from the four groups of mice tumors and examination of the impact of neutrophil depletion on BF10 treatment, related to Figure 6. (A)-(B). GSEA plots of BF10 versus Ctrl **(A)** and BF10 versus IL-10 **(B).** Related pathways were assessed by GSEA gene set of reactive oxygen species pathway, interferon-alpha response, interferon-gamma response, and inflammatory response signaling. The normalized enrichment

score (NES) and p-value are shown in each panel. **(C)** Experimental scheme and treatment protocol of HNSCC/Q1-2 tumor-bearing mice with neutrophil depletion. **(D)** Tumor volume curves of individual mice treated with Ctrl-IgG, BF10, NIMP-R14 or BF10 combined NIMP-R14. **(E)** The monitoring of tumor volume change in HNSCC/Q1-2 tumor-bearing mice treated with Ctrl-IgG, BF10, NIMP-R14 and BF10 combined NIMP-R14. (**F).** Survival analysis between groups of Ctrl-IgG-, BF10-, NIMP-R14- and BF10 combined NIMP-R14-treated HNSCC/Q1-2 tumor-bearing mice. P value was estimated by log-rank test.

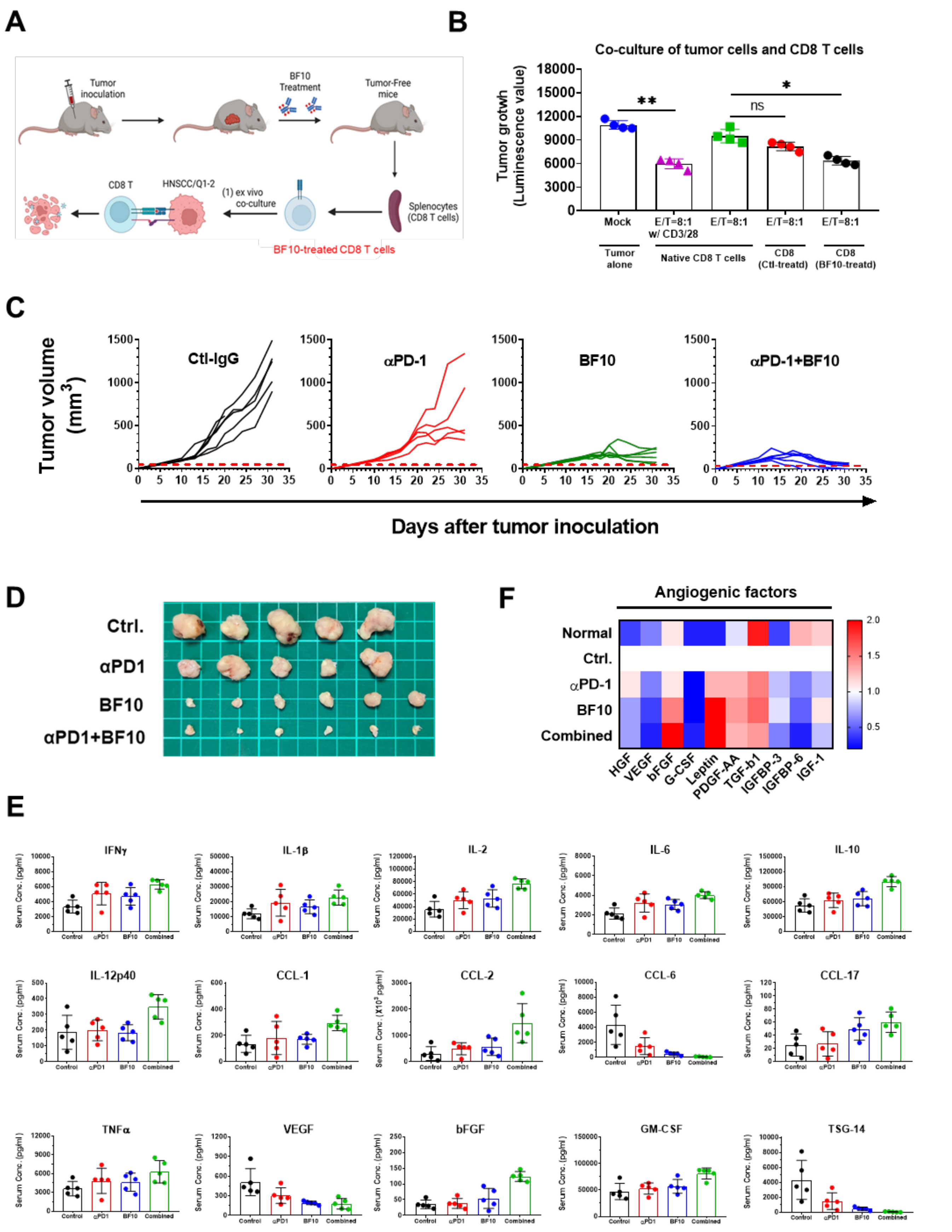

Figure S11. BF10 elicits durable protect effect and potentiates anti-PD1 efficacy, related to Figure 7. (A)-(B) Schema of the co-culture assay of CD8 T cells and tumor cells (a). HNSC/Q1-2 tumor cells (3x10^5 cells) were inoculated subcutaneously into mice, followed by BF10 treatment until tumor regression. CD8 T cells were isolated and co-cultured with tumor cells (E:T ratio = 8:1) in the presence or absence of CD3/CD28 stimulation. After 72 hours, the photos of tumor cell growth were recorded. The quantification of tumor growth inhibition of indicated groups. **(C)-(D)** The combinational efficacy of BF10 with anti-PD1

treatment in HNSC mouse model. Tumor-bearing mice (Q1-2, subcutaneous) received isotype control or anti-PD1 (200 μg) and/or BF10 (36 mg/kg) as indicated (n=5 to 6 per group). Tumor volume curves of individual mice **(C)**. Representative photos on day 31 after tumor inoculations **(D). (E)-(F)** Mouse serum from indicated treatments were analyzed by the Mouse Cytokine Antibody Array. n=4 for each group. Serum concentration of the analyzed cytokines **(E)**. A heatmap for summarizing the level of angiogenic factors **(F)**.
